# Convalescent plasma for adults with acute COVID-19 respiratory illness (CONCOR-1): study protocol for an international, multicentre, randomized, open-label trial

**DOI:** 10.1186/s13063-021-05235-3

**Published:** 2021-05-04

**Authors:** Philippe Bégin, Jeannie Callum, Nancy M. Heddle, Richard Cook, Michelle P. Zeller, Alan Tinmouth, Dean A. Fergusson, Melissa M. Cushing, Marshall J. Glesby, Michaël Chassé, Dana V. Devine, Nancy Robitalle, Renée Bazin, Nadine Shehata, Andrés Finzi, Allison McGeer, Damon C. Scales, Lisa Schwartz, Alexis F. Turgeon, Ryan Zarychanski, Nick Daneman, Richard Carl, Luiz Amorim, Caroline Gabe, Martin Ellis, Bruce S. Sachais, Kent Cadogan Loftsgard, Erin Jamula, Julie Carruthers, Joanne Duncan, Kayla Lucier, Na Li, Yang Liu, Chantal Armali, Amie Kron, Dimpy Modi, Marie-Christine Auclair, Sabrina Cerro, Meda Avram, Donald M. Arnold

**Affiliations:** 1 Section of Allergy, Immunology and Rheumatology, Department of Pediatrics, CHU Sainte-Justine, Montreal, Quebec Canada; 2 Department of Medicine, CHUM, Université de Montréal, Montreal, Quebec Canada; 3 Department of Laboratory Medicine and Molecular Diagnostics, Sunnybrook Health Sciences Centre, Toronto, Ontario Canada; 4 Department of Laboratory Medicine and Pathobiology, University of Toronto, Toronto, Ontario Canada; 5 Department of Medicine, McMaster University, Hamilton, Ontario Canada; 6 McMaster Centre for Transfusion Research, McMaster University, Hamilton, Ontario Canada; 7 Department of Statistics and Actuarial Science, University of Waterloo, Waterloo, Ontario Canada; 8 Department of Medicine, Ottawa Hospital, University of Ottawa, Ottawa, Ontario Canada; 9 Ottawa Hospital Centre for Transfusion Research, Ottawa Hospital Research Institute, Ottawa, Ontario Canada; 10 Canadian Blood Services, Ottawa, Ontario Canada; 11 Clinical Epidemiology Program, Ottawa Hospital Research Institute, Ottawa, Ontario Canada; 12 Department of Medicine, University of Ottawa, Ottawa, Ontario Canada; 13 Adjunct Scientist, Canadian Blood Services, Ottawa, Ontario Canada; 14 Transfusion Medicine and Cellular Therapy, NewYork-Presbyterian, New York, NY USA; 15 Department of Pathology and Laboratory Medicine, Weill Cornell Medicine, New York, NY USA; 16 Division of Infectious Diseases, Weill Cornell Medical College, Weill Cornell Medicine, New York, NY USA; 17 Department of Medicine (Critical Care), University of Montreal Health Centre (CHUM), Montreal, Quebec Canada; 18 Department of Medicine, University of Montreal, Montreal, Quebec Canada; 19 Department of Pathology and Laboratory Medicine, University of British Columbia, Ottawa, Ontario Canada; 20 Héma-Québec, Saint-Laurent, Montreal, Canada; 21 Division of Hematology and Oncology, Department of Pediatrics, CHU Sainte-Justine, Université de Montréal, Ottawa, Ontario Canada; 22 Medical Affairs and Innovation, Héma-Québec, Saint-Laurent, Montreal, Canada; 23 Departments of Medicine, Laboratory Medicine and Pathobiology, Institute of Health Policy Management and Evaluation, University of Toronto, Toronto, Ontario Canada; 24 Division of Hematology, Mount Sinai Hospital, Toronto, Ontario Canada; 25 Canadian Blood Services, Toronto, Ontario Canada; 26 Département de Microbiologie, Infectiologie et Immunologie, Université de Montréal, Montreal, Quebec Canada; 27 CHUM Research Center, Montreal, Quebec Canada; 28 Department of Microbiology, Sinai Health System, Toronto, Ontario Canada; 29 Department of Laboratory Medicine and Pathobiology and Dalla Lana School of Public Health, University of Toronto, Toronto, Ontario Canada; 30 Department of Critical Care Medicine, Sunnybrook Health Sciences Centre, Toronto, Ontario Canada; 31 Department of Medicine, Interdepartmental Division of Critical Care, University of Toronto, Toronto, Ontario Canada; 32 Department of Health Research Methods, Evidence & Impact, Faculty of Health Sciences, McMaster University, Hamilton, Ontario Canada; 33 Department of Anesthesiology and Critical Care Medicine, Division of Critical Care Medicine, Faculty of Medicine, Université Laval, Quebec, Quebec Canada; 34 CHU de Québec – Université Laval Research Centre, Population Health and Optimal Health Practices Research Unit, Trauma - Emergency - Critical Care Medicine, Université Laval, Quebec, Quebec Canada; 35 Department of Internal Medicine, Sections of Hematology/Medical Oncology and Critical Care, University of Manitoba, Winnipeg, Manitoba Canada; 36 Department of Medicine, Division of Infectious Diseases, Sunnybrook Health Sciences Centre, University of Toronto, Toronto, Ontario Canada; 37 Hemorio, Rio de Janeiro, Brazil; 38 Hematology Institute and Blood Bank, Meir Medical Center, Tel Aviv, Israël; 39 Sackler School of Medicine, Tel Aviv University, Tel Aviv, Israël; 40 New York Blood Center Enterprises, New York, NY USA; 41 Department of Computing and Software, McMaster University, Hamilton, Ontario Canada; 42 Department of Community Health Sciences, University of Calgary, Hamilton, Ontario Canada; 43 Clinical Research Department, Centre de recherche du CHU Sainte-Justine, Centre Hospitalier Universitaire Sainte-Justine Centre, Montreal, Canada

**Keywords:** Convalescent plasma, SARS-CoV-2, Coronavirus, COVID-19, Randomized controlled trial

## Abstract

**Background:**

Convalescent plasma has been used for numerous viral diseases including influenza, severe acute respiratory syndrome, Middle East respiratory syndrome and Ebola virus; however, evidence to support its use is weak. SARS-CoV-2 is a novel coronavirus responsible for the 2019 global pandemic of COVID-19 community acquired pneumonia. We have undertaken a randomized controlled trial to assess the efficacy and safety of COVID-19 convalescent plasma (CCP) in patients with SARS-CoV-2 infection.

**Methods:**

CONCOR-1 is an open-label, multicentre, randomized trial. Inclusion criteria include the following: patients > 16 years, admitted to hospital with COVID-19 infection, receiving supplemental oxygen for respiratory complications of COVID-19, and availability of blood group compatible CCP. Exclusion criteria are : onset of respiratory symptoms more than 12 days prior to randomization, intubated or imminent plan for intubation, and previous severe reactions to plasma. Consenting patients are randomized 2:1 to receive either approximately 500 mL of CCP or standard of care. CCP is collected from donors who have recovered from COVID-19 and who have detectable anti-SARS-CoV-2 antibodies quantified serologically. The primary outcome is intubation or death at day 30. Secondary outcomes include ventilator-free days, length of stay in intensive care or hospital, transfusion reactions, serious adverse events, and reduction in SARS-CoV-2 viral load. Exploratory analyses include patients who received CCP containing high titre antibodies. A sample size of 1200 patients gives 80% power to detect a 25% relative risk reduction assuming a 30% baseline risk of intubation or death at 30 days (two-sided test; *α* = 0.05). An interim analysis and sample size re-estimation will be done by an unblinded independent biostatistician after primary outcome data are available for 50% of the target recruitment (*n* = 600).

**Discussion:**

This trial will determine whether CCP will reduce intubation or death non-intubated adults with COVID-19. The trial will also provide information on the role of and thresholds for SARS-CoV-2 antibody titres and neutralization assays for donor qualification.

**Trial registration:**

Clinicaltrials.govNCT04348656. Registered on 16 April 2020.

## Background

COVID-19 is an acute viral illness caused by the novel SARS-CoV-2 coronavirus. With a high number of infections , therapeutic options are urgently needed to mitigate morbidity and mortality [1]. One potential therapeutic option is convalescent plasma collected from individuals who have recovered from COVID-19 [2]. COVID-19 convalescent plasma (CCP) contains anti-SARS-CoV-2 antibodies, which can neutralize the virus, reduce viral load, and potentially improve clinical outcomes [3].

Convalescent plasma has been used during other pandemics. During the severe acute respiratory syndrome (SARS) epidemic, a non-randomized study from Hong Kong (*n* = 80) showed a reduction in mortality with convalescent plasma compared with historical controls (13% vs. 17% respectively) [4]. For influenza, an observational study (*n* = 93) of convalescent plasma showed a reduction in mortality, with an odds ratio (OR) of 0.20 [95% confidence interval (CI), 0.06–0.69] [5], and a phase 3 randomized trial (*n* = 200) using hyperimmune plasma did not show an improvement in clinical status on day 7 [6]. In Ebola, improved survival was observed in a non-randomized study of convalescent whole blood [7], but not with convalescent plasma [8]. A systematic review of convalescent plasma for viral pneumonias caused by severe influenza or coronavirus infection (SARS-CoV-1 or Middle East respiratory syndrome (MERS)) included 32 non-randomized studies (*n* = 1327). The conclusion based on very low-quality evidence was that convalescent plasma may be associated with a reduction in mortality, length of stay, and duration of ventilation and that well-designed clinical trials are needed [9]. A recent systematic review that included four randomized controlled trials (RCTs) and two non-randomized studies of convalescent plasma or hyperimmune products showed low-quality evidence for lack of harm and insufficient evidence of efficacy [10].

Case series of CCP use in patients with COVID-19 from China reported positive results with CCP administered before 22 days of symptom onset [3, 11, 12]. Conversely, other studies reported no benefit when CCP was administered later in the course of disease (> 21 days) [13]. A randomized trial from China (*n* = 103) reported no improvement in outcomes with CCP although the direction of the effect suggested benefit for patients with severe but not life-threatening disease [14]. Another RCT (*n* = 86) from the Netherlands reported no improvement with CCP but the trial was stopped early when high titre anti-SARS-CoV2 antibodies were detected in patients at baseline [15]. An RCT from India (*n* = 464) showed no difference in mortality or disease progression, but CCP donors were not screened for antibody titres [16]. An RCT from Argentina (n= 228) showed no benefit of CCP compared with placebo for improvement in clinical status by day 30 (**Please insert this new reference: Siminovich et al PMID: 33232588**), while another plaebo-controlled RCT from Argentina (n= 160) showed a reduction in the development of severe respiratory disease when high-titre CCP was given early in the disease course (within 72 hours of symptom onset) (*** Please insert this new reference: Libster et al PMID:33406353**)  On March 25, 2020, the US FDA launched an Expanded Access Program for CCP [17]. An observational study of 35,322 patients who received CCP under that program suggested that CCP was most effective when given early in the disease course and with high titre anti-SARS-CoV2 antibodies [18]. A previous report of 20,000 transfused patients showed few adverse events with CCP (< 1%) such as allergic reactions, transfusion-associated circulatory overload (TACO), transfusion-related acute lung injury (TRALI), thromboembolic events, hypotension, or cardiac events [19, 20]. A theoretical risk of CCP is antibody-dependent enhancement of infection [2, 21].

## Methods/design

### Objective

CCP remains an unproven treatment for COVID-19. CONCOR-1 (CONvalescent Plasma for Hospitalized Adults with Acute COVID-19 Respiratory Illness) is a multicentre, open-label superiority trial designed to answer whether CCP, collected from individuals who have recovered from COVID-19 infection that contains anti-SARS-CoV-2 antibodies, decreases the risk of intubation or death at day 30 in hospitalized adult patients with acute COVID-19 respiratory illness compared to standard of care. We hypothesize that CCP will reduce the risk of intubation and death at day 30 and improve other outcomes including the need for critical care support, duration of hospitalization, and quality of life. Results of serological testing on the CCP will be correlated with patient outcomes.

### Trial design

**Fig. 1:**
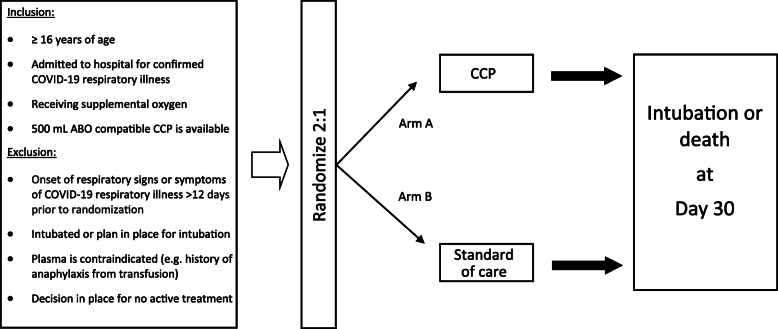
Trial overview

CONCOR-1 is a parallel arm, multicentre, open-label, superiority randomized controlled trial comparing CCP to standard of care (Fig. 1). Centres in Canada, the United States of America (USA), and Brazil  are invited to participate. Eligible patients are  randomized using a 2:1 allocation to receive either CCP or standard of care. The protocol was approved by all Research Ethics Boards at participating sites, the blood collection facilities, Health Canada (Control # 238201), the United States Food and Drug Administration (IND 22075), and the National Research Ethics Commission (Comissão Nacional de Ética em Pesquisa) in Brazil (approval 4.305.792). The trial was registered at clinicaltrials.gov (NCT04348656).

### Study setting

The trial will take place at approximately 80 academic and community hospitals in Canada, USA, and Brazil. A complete list of active study sites can be found in the trial record at clinicaltrials.gov.

### Eligible patients

Patients are eligible for the trial if (1) age > 16 years; (2) hospitalized with confirmed SARS-CoV-2 infection; (3) receiving supplemental oxygen for COVID-19 respiratory illness; (4) ABO-compatible CCP is available. Exclusion criteria are : (1) onset of respiratory signs or symptoms > 12 days before randomization; (2) currently intubated or a plan is in place for intubation; (3) a plan is in place for no active treatment; or (4) contraindication to plasma transfusion (e.g. history of anaphylaxis, refusal of blood products). These criteria are meant to capture patients who are early in the disease course and at risk of worsening respiratory illness. Co-enrolment in other interventional trials is permitted, with the exception of other CCP trials. We will collect data on all co-interventions and co-enrolment.

### Recruitment

Sites work with their local infectious disease team to determine a strategy for identifying admitted patients who test positive for COVID-19 and to communicate these results to the research team. Infectious disease physicians act as site co-investigators wherever possible to facilitate this process. Sites submit screening data on a weekly basis to the methods centre, which include reasons for screen failures. Screening logs are  reviewed regularly by the trial Executive Committee to identify common exclusions and strategies to optimize recruitment.

### Informed consent

Informed consent is  obtained from each participant by authorized study personnel prior to any study procedures taking place. Informed consent may be written or verbal. Obtaining verbal informed consent remotely is permitted to avoid exposing research staff to COVID-19 and may be facilitated by telephone or video-assisted consent tools. Where required by the local research ethics board, written informed consent is obtained from the patient or legally authorized representative who provided verbal consent, once safe to do so. Participants provide consent for use of blood samples for study testing as described in the protocol and for publication of the results using aggregate data. No additional biobanking is planned.

### Randomization

Allocation sequence generation is  performed by an independent biostatistician using a computer-generated algorithm and stratified by centre and age (< 60 and ≥ 60 years). Age is included as a stratum variable due to known increased mortality with age. The randomization sequence is generated using a secure, concealed, computer-generated system and blocked with random block sizes to ensure concealment. The allocation sequence is maintained securely within the electronic data capture software system and accessible only to the independent, unblinded statistician. Participants are randomized by authorized study personnel at each site who will assign participants to interventions using a centralized website. Access to this website is restricted to trained study individuals via a unique username and password.

### Intervention

The experimental intervention is a single dose of approximately 500 mL of CCP. Apheresis CCP is will be collected by Canadian Blood Services (CBS), Héma-Québec (HQ), the New York Blood Center (NYBC), and Hemorio (Brazil). CCP donors must meet all donor eligibility criteria for routine apheresis plasma donation plus (1) confirmed COVID-19 infection by nasal swab or by antibody testing; (2) complete resolution of symptoms for at least 14 days; (3) male donors, or female with no pregnancy history or with a negative HLA antibody test result; and (4) ≥ 6 days since last plasma donation. CCP will be compatible for ABO blood group or have evidence of low anti-A or anti-B titres (< 1:50). CCP is e transfused by nursing staff as a single dose of approximately 500 mL from one donor, or two doses of 250 mL from one or two donors, administered within 24 h of randomization. The 500 mL dose of CCP is  infused over 4 h, and patients are  monitored for adverse events during the infusion. This dose of CCP is consistent with previous CCP studies [3, 5, 22].

**Fig. 2:**
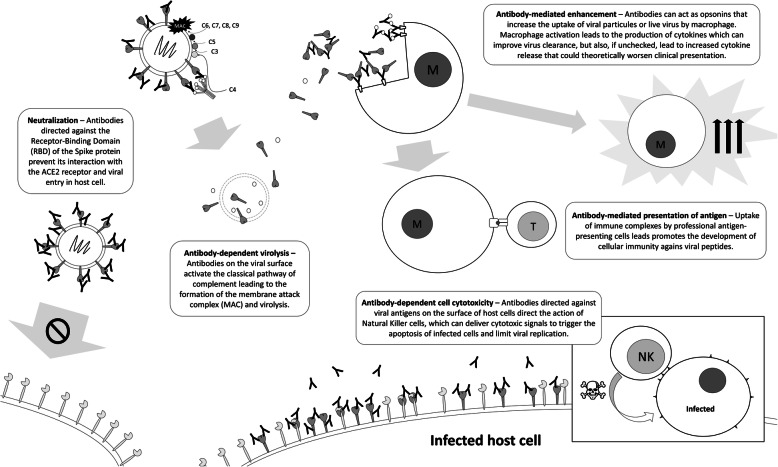
Possible mechanisms of action of passively transferred antibodies in COVID-19. These include viral neutralization, complement-mediated antibody-dependent virolysis, antibody-dependent enhancement of immune response, antibody-mediated presentation of antigen, and antibody-dependent cell cytotoxicity. Of these, only viral neutralization is measured by neutralization assays currently used to qualify convalescent plasma

Anti-SARS-CoV-2 antibodies in donor plasma is measured  by ELISA or cyclic enhanced immunofluorescence assay with a minimum titre of 1:100, or by neutralization assay with a minimum titre of 1:160. As there are no scientific data to support one qualifying antibody test over another, the blood suppliers were permitted to select the qualifying titre and methodology independently based on availability. The FDA has recommended the use of minimal titre of neutralizing antibodies of 1:160 (or 1:80 when no compatible units are available), recognizing that these thresholds lack validation and other biomarkers may be important (e.g. antibody-dependent cell cytotoxicity, complement-mediated virolysis, or antibody-dependent presentation of antigens) (Fig. 2) [6, 23, 24]. At the end of the trial, all CPP units will be tested for neutralizing antibodies to correlate with clinical outcomes. Information collected on CCP donors includes donor age, date of onset and severity of COVID-19 symptoms, and SARS-CoV-2 antibody titres.

The control arm of the trial is standard of care. Standard of care treatment was not prescribed in the protocol. It consistsof supportive care treatments for respiratory illness, and specific treatments for COVID-19 available [25]. An open-label design was justified since the primary outcomes (intubation or death) are objective in nature. In addition, masking procedures such as plasma bag covers and additional labeling of plasma units would have imposed significant challenges to blood bank personnel during the pandemic, which would have have made the trial infeasible in many centres. The use of standard plasma as the control was not felt to be justified because of the potential harm with no anticipated benefit. The 2:1 allocation is anticipated to maximize acceptability by patients and local investigators and will improve power for sub-analyses of antibody titre.

Modifications to the intervention are not expected. Adherence to this one-time intervention will be monitored and is expected to be high; strategies to improve adherence will be developed if needed. The CCP infusion should bewill be stopped if an adverse event occurs that is deemed to be related to the CCP or at the patient’s request. Discontinuation practices align with local clinical policies regarding management of transfusion reactions. Participants will receive routine clinical follow-up care as required for any harm caused by the trial.

All participants receive treatment as per local standard practice. In the rare event that a trial participant requires standard plasma for a non-study reason, local transfusion medicine labs will provide plasma with the longest storage duration (plasma is routinely stored for up to 1 year) to reduce of contamination with anti-SARS-CoV-2 antibodies. All blood products received during the trial will be documented.

### Outcomes

The primary outcome is a composite of intubation or death at day 30. The primary outcome was chosen due to its clinical relevance and objective assessment. Secondary outcomes are time to intubation or death, ventilator-free days, in-hospital death by day 90, time to in-hospital death, death by day 30, ICU length of stay, hospital length of stay, need for extracorporeal membrane oxygenation (ECMO), need for renal replacement therapy, myocarditis, patient-reported outcome as measured by EQ-5D-5 L, incremental cost per quality adjusted year, CCP transfusion-related adverse events and serious adverse events. Viral load from blood samples collected before and 48 h after randomization will be measured to determine the impact of CCP on viral clearance.

### Adverse event reporting and harms

All transfusion-associated adverse events and adverse events with a severity of grade 3 or higher are  captured. Adverse events are  classified using the Medical Dictionary for Regulatory Activities (MedDRA) [26] and graded by the Common Terminology Criteria for Adverse Events (CTCAE) version 4.0 criteria [27]. Terminology and grading from the International Society on Blood Transfusion are  recorded for transfusion-associated adverse events [28].

### Events requiring expedited reporting

Suspected unexpected serious adverse reactions (SUSAR) are  reported to the sponsor within 24 h. These are events that have a reasonable causal relationship to the CCP transfusion, are considered unexpected, and meet any of the following criteria for a serious adverse event:

1. Results in death;
2. Is life-threatening; this means that the subject is at risk of death at the time of the event; it does not mean that the event hypothetically might have caused death if it was more severe;
3. Requires hospitalization (overnight or longer) or prolongation of existing hospitalization or invasive procedure;
4. Results in persistent or significant disability or incapacity;
5. Results in congenital anomaly or birth defect;
6. Is not be immediately life-threatening or result in death or hospitalization but may jeopardize the subject or require intervention to prevent one of the above outcomes.

Any TRALI or TACO event is  reported to the sponsor within 24 h.

**Fig. 3:**
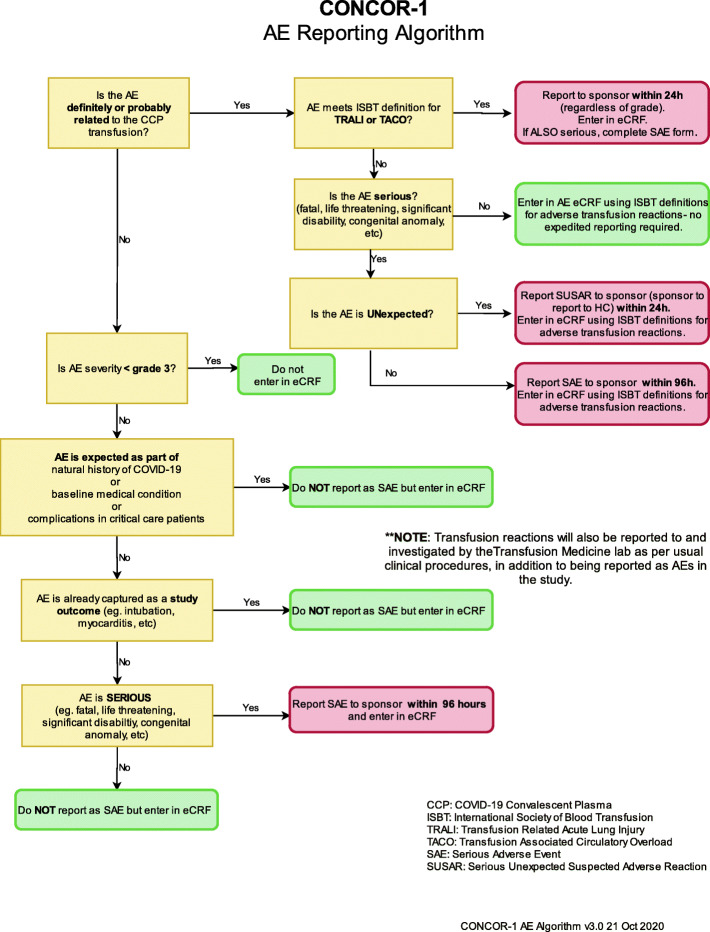
Flow chart for AE and SAE reporting in the trial

Other unexpected serious adverse events which are not related to CCP are reported within 96 h, if they meet reporting criteria. Due to the critical nature of illness of the study population, a revised definition of a reportable serious adverse event (SAE) was developed for use in this trial based off of guidelines used in critical care trials [29]. Therefore, the following events are not considered reportable SAEs for the purposes of the trial: AEs that are part of the natural history of the primary disease process or expected complications of critical illness; AEs expected in the context of a baseline medical condition; and AEs that are already captured as study outcomes. Figure 3 shows the flow diagram for adverse event reporting.

### Participant timeline

Patients admitted to hospital with a positive COVID-19 test result are assessed for eligibility. Eligible patients who have consented are  randomized as soon as possible (day of randomization is considered day 1). The assessment schedule for study patients is summarized in Table 1.

**Table 1:** Schedule of patient assessments

			Follow-up	
Visit	Screening	Baseline (D1)	D2–D30^(1)^ (or until discharge)	D30^(2)^ (if patients discharged^)(10)^
Time window (days)				+/− 3 days
Informed consent/assent	X			
Verify eligibility criteria	X			
Randomization		X		
CCP administration ^(3)^		X		
Demographics		X		
Comorbidities		X		
COVID-19 history^(4)^		X		
Height and weight		X		
Vital signs		X		
SpO_2_		X	X	
FiO_2_		X	X	
COVID treatments		X	X	X
Hospitalization daily assessment ^(5)^		X	X	
Results of blood tests (done as per clinical need)		X	X^(6)^	
EQ-5D^(7)^		X		X
Vital status (dead/ alive)			X	X
Adverse event review		X	X	X
Discharge status ^(8)^				X
Mechanical ventilation since discharge				X
Viral load, cytokines and anti-CoV-2 titres^(9)^		X	X	

^(1)^ Only collected if still hospitalized

^(2)^ Phone call if subject is already discharged from the hospital

^(3)^ Only for subject randomized to the CCP treatment

^(4)^ Includes date of first symptoms; description of symptoms and date of diagnostic test

^(5)^ Mechanical ventilation, ECMO, renal replacement therapy, or myocarditis

^(6)^ Daily from day 1 to 7, then day 14 ± 3 days

^(7)^ EQ-5D to be collected on day 30 regardless of hospitalization status

^(8)^ Discharge or hospital status (admitted, home, local hospital, long-term care facility)

^(9)^ To be performed using frozen leftover sample or extra tube collected once at baseline and once during follow-up between D2 and D6

^(10)^ For patients still hospitalized at day 30, chart review will be conducted at day 90 to determine whether the patient was discharged or died in hospital between day 30 and day 90 or is still in hospital at day 90

### Blinding

CONCOR-1 is an open-label trial. The use of objective outcomes of death and intubation will mitigate bias as a result of the open-label design. The statistician performing the final analysis will remain blinded throughout the trial. Blinded safety reports will be generated at regular intervals for the Executive Committee, Independent Data Safety Monitoring Committee (IDSMC), and Steering Committee.

### Data collection

Baseline, daily physiologic and laboratory data, and outcome data are  collected from randomization to day 30 (Table 1). Data are entered into an electronic data capture system (EDC) by site personnel. The only required blood samples for the trial are the ABO blood group for determination of CCP compatibility and two samples for testing of viral load and antibody levels, which are  sent to a central laboratory. Other laboratory tests are captured if ordered as part of routine patient care. All laboratory tests have been validated and undergo routine quality control as part of the clinical laboratories’ quality management program.

Samples from all blood donors are  tested for antibodies against SARS-CoV2 using quantitative and functional assays at centralized laboratories. No additional tests are planned for this trial.

Quality of life assessment is performed using the 5-level EQ-5D instrument at baseline and at day 30 [30]. Data capture from electronic clinical charts and data entry are performed remotely wherever possible to mitigate infection risk to research personnel. Standardized training is provided to all site staff performing data collection and entry prior to site activation in the form of training videos, with proof of completion required to obtain a training certificate. An Operations Manual is provided to sites with detailed instructions for data collection and use of the forms within the EDC.

Participant retention and complete follow-up is not anticipated to be problematic as beyond day 1 (CCP infusion) and a follow-up contact at day 30, participants are not required to perform any additional trial-specific activities. The trial was designed to reduce burden on participants and promote retention. Participants who discontinue or deviate from the study protocol will still have all study data collected, unless they withdraw consent for study participation.

### Data management

The CONCOR-1 database and randomization platform utilizes the REDCap (Research Electronic Data Capture) software, located on a secure authenticated server at the CONCOR-1 methods centre, McMaster University Medical Centre, Hamilton, Ontario, Canada [31, 32]. REDCap is a secure, web-based software platform designed to support data capture for research studies. Study patient data may be initially collected on paper CRFs (Additional file 1) and consent and eligibility confirmed before being entered into the eCRF. Upwards of 60 hospitals (or hospital groups) may participate, each able to input and access their own site data. The responsibilities of the sites for collection and inputting of data, timeliness, and responding to data queries are outlined in the Operations Manual. A comprehensive description of the longitudinal design of the trial, site eCRF training, electronic query/resolution workflow for ongoing quality review (based on documented data cleaning rules and validation rules), and access to data are documented in the CONCOR-1 Data Management Plan. All paper CRFs are found in the REDCap database file repository folder for easy download by sites, along with a link to all trial procedures and protocols.

### Sample size estimation

We estimate that 1200 patients will be required to achieve 80% power to detect a relative risk reduction of 25% with CCP (2-tailed test at level α = 0.05 and 2:1 allocation), assuming a 30% baseline risk of intubation or death at day 30 with standard of care. This standard of care event rate estimate was derived from Canadian data where the risk of ICU admission among hospitalized patients was 23% [33], and data from New York City where up to 41.5% of hospitalized patients required mechanical ventilation [34]. Because of uncertainty of baseline event rate, a sample size re-estimation is planned for  when the primary outcome is available for 50% of the target sample. Sample size can  be adjusted upwards if the observed event rate suggests that the trial is underpowered for the test based on the primary outcome.

### Statistical analysis plan

The statistical analysis plan is provided in Additional file 2. A single interim analysis is planned when data on the primary outcome are available for 50% of the target sample. The Lan-DeMets spending function [35] analog of the O’Brien-Fleming stopping rule [36] will be used to monitor the primary endpoint to guide the IDSMC. Conditional power will be presented for futility analysis as additional information for the IDSMC. A recommendation for premature trial termination may be made if there is reasonable cause, including but not limited to (1) a concern regarding an unacceptable risk to subjects, (2) results of the interim analysis demonstrating superiority, (3) results of the interim analysis for the revised sample size making the resultant trial infeasible due to the large number of patients required, (4) evidence from the futility analysis is compelling, (5) overall compliance with the protocol is poor, (6) data that are not sufficiently complete and/or are not evaluable, (7) regulatory authorities recommend termination of the trial, or (8) novel scientific data on the efficacy or safety of CCP becomes available raising concerns about the safety of CCP.

The primary outcome will be analysed using a 2-sided Wald test of the null hypothesis that probability of intubation or death at day 30 is the same among individuals receiving CCP or standard of care with a relative risk and an associated 95% confidence interval presented. A secondary analysis of time to event responses will be modeled using cause-specific Cox regression models accommodating censoring at the time of study withdrawal or upon the occurrence of competing events.

For secondary outcomes the restricted mean number of ventilator-free days will be compared using a nonparametric analysis. The survival status at day 30 and day 90 will be compared based on a comparison of binomial proportions, and the proportion of patients needing ECMO, needing renal replacement therapy, and/or with myocarditis will be compared between the two arms. Subgroup analyses will include:

- Co-enrolment in other therapeutic trials
- Age (> 60)
- Sex
- Ethnicity
- Obesity
- Medical comorbidities (diabetes, cardiac, respiratory)
- Smoking status (> 15 pack year history; never/ever/current smoker)
- Number of CCP donors (one vs. two)
- Severity of illness in donors (hospitalized vs. not)
- Onset of any symptom (> 12 days vs. ≤ 12 days)
- Timing of administration of CCP from diagnosis (≤ 72 h vs. > 72 h)
- ABO blood type of recipient
- Detectable vs non-detectable viral plasma viral load at baseline
- Anti-SARS-CoV-2 seropositivity at baseline
- Oxygen status at baseline (≤ 2 L vs > 2 L)

We expect to have complete data on individuals for the primary and secondary outcomes and we will make all efforts to minimize loss to follow-up. If an individual withdraws from the trial early, their time to event outcomes will be censored at the time of withdrawal. For binary outcomes where data are missing, the first analysis will be based on a completed cases (modified intention to treat) sample. A second analysis will deal with potential bias from using a complete case analysis by imputing missing data via imputation models fitted to the data from individuals providing complete information. Major protocol deviations and violations will be entered in the EDC in real time and monitored monthly throughout the trial by a subgroup of individuals from the Executive Committee and corrective action taken whenever possible. The Statistical Analysis Plan provides details related to handling of missing data and non-adherence to the protocol.

### Confidentiality

No identifying information is  collected until patients consent  to trial participation. If allowed by local ethics boards, screening logs may contain patient initials but this information remains at the study site. For enrolled participants, case report forms (both paper and electronic) are identified by a unique study identification number. The study key containing the study identification number and patient identifiers will be securely stored at each study site. Any source documentation required to be sent off-site will be de-identified prior to transmission. Sites will store all study records securely, with access restricted to authorized individuals, for the duration of the trial and required retention period.

### Data monitoring

The IDSMC is comprised of six individuals with expertise in biostatistics, transfusion medicine, infectious disease, and clinical research methodology (see IDSMC charter- Additional file 3). The first official meeting is planned for when the day 30 follow-up is completed for the first 20 randomized patients. Report formats will be reviewed and approved by the IDSMC. After the initial meeting, the IDSMC will meet when complete data is available for every 100 patients enrolled to review operational and safety data (see “Adverse event reporting and harms” section). The IDSMC will also receive monthly safety reports. The IDSMC will report to the study sponsor and the study Executive Committee, which includes the co-principal investigators.

The interim analysis is described in the Statistical Analysis Plan section. The results of the interim analysis will be unblinded for the independent biostatistician performing the analysis and the members of the IDSMC. All other study personnel and investigators will remain blinded to the results of the interim analysis. The primary purpose of the interim analysis is for sample size re-estimation. An O’Brien-Fleming stopping rule [36] will be used at that time, but treated as a guideline, so there is minimal impact on the threshold for statistical significance for the final significance test of the primary outcome. The IDSMC can make recommendations related to stopping for efficacy, futility, and/or safety, but the Steering Committee will be responsible for making the final decision.

### Auditing

An independent third-party auditor facilitates site activation, verifies accuracy of data entered into the electronic case report form, and oversees GCP compliance on behalf of the sponsor. A detailed auditing plan describes the procedures and frequency of audits and the documents to be reviewed. Visits may be performed remotely as required by pandemic restrictions, or may take place on-site. In the event of a remote auditing visit, de-identified source documentation will be provided to the auditors. Audit reports will be provided to the sponsor after each visit. Visits will be performed after the first patient has been enrolled at a site, and regularly thereafter once a certain number of patients have subsequently been enrolled.

### Trial management

#### Methods centre and coordinating centres

**Fig. 4:**
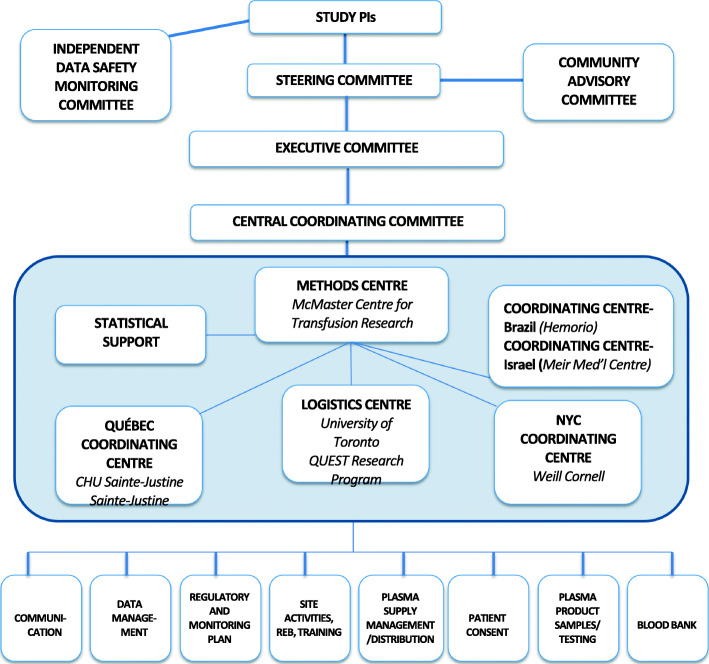
Organizational chart for the CONCOR-1 trial

The methods centre for the trial is the McMaster Centre for Transfusion Research, McMaster University. The methods centre will work directly with the blood suppliers to ensure the effective operations of all aspects of the trial. The methods centre is responsible for regulatory requirements, data management, and data analysis and oversees the working groups for CCP distribution and communications, as well as regulatory affairs, site activation, and blood banks outside Québec. The logistics centre (University of Toronto, QUEST research team) is responsible for developing study procedures, site blood bank and clinical team training modules, site support, and site recruitment outside Québec. The Québec coordinating centre (Ste-Justine, Université de Montréal) is responsible for site recruitment, training and activation, regulatory affairs, and blood banks in Québec, as well as serological analyses. The New York coordinating centre (Weill Cornell Medicine) will work with NYBC (supplier of CCP) and is responsible for regulatory requirements in the USA and for the coordination of the three New York hospital sites. Hemorio will act as the coordinating centre in Brazil and will oversee CCP supply in that country. Each coordinating centre includes physicians with transfusion medicine and/or immunological expertise, methodologists, experienced research coordinators, and research and administrative support staff (Fig. 4).

#### Trial oversight

The Steering Committee (SC) has the overall responsibility for the design, execution, analysis interpretation, and publication of results of the trial. The SC includes the broad range of clinical expertise from haematology, immunology, virology, critical care, research methodology, transfusion medicine required for the conduct of this large-scale RCT, blood supplier representatives, and a patient advisor.

SC members, affiliations, and areas of expertise are as follows:

- Dr. Donald Arnold; Department of Medicine, McMaster University; Hamilton, Ontario, Canada (haematology; transfusion medicine)
- Dr. Philippe Bégin; Department of Pediatrics, CHU Sainte-Justine; Department of Medicine, CHUM, Université de Montréal; Montréal, Québec, Canada (immunology)
- Dr. Jeannie Callum; Department of Laboratory Medicine and Molecular Diagnostics, Sunnybrook Health Sciences Centre; Department of Laboratory Medicine and Pathobiology, University of Toronto; Toronto, Ontario, Canada (transfusion medicine)
- Prof Nancy Heddle; Department of Medicine, McMaster University; Hamilton, Ontario, Canada (transfusion medicine, clinical trial methodology)
- Dr. Richard Cook; Department of Statistics and Actuarial Science, University of Waterloo; Waterloo, Ontario, Canada (statistics; clinical trial design and analysis)
- Dr. Michelle Zeller; Canadian Blood Services; Department of Medicine, McMaster University; Hamilton, Ontario, Canada (transfusion medicine)
- Dr. Alan Tinmouth; Department of Medicine, University of Ottawa; Ottawa Hospital Research Institute; Canadian Blood Services, Ottawa, Ontario, Canada (haematology, transfusion medicine)
- Dr. Dean Fergusson; Ottawa Hospital Research Institute; Department of Medicine, University of Ottawa; Canadian Blood Services (clinical trial methodology, knowledge translation, transfusion medicine)
- Dr. Melissa Cushing; Transfusion Medicine and Cellular Therapy, NewYork-Presbyterian; Department of Pathology and Laboratory Medicine, Weill Cornell Medicine; New York City and Ithaca, New York, USA (transfusion medicine)
- Dr. Michaël Chassé; Department of Medicine (Critical Care), University of Montreal Health Centre (CHUM); Department of Medicine, University of Montreal; Montréal, Québec, Canada (critical care, clinical trial methodology)
- Dr. Dana Devine; Canadian Blood Services; Department of Pathology and Laboratory Medicine, University of British Columbia; Vancouver, British Columbia, Canada (transfusion medicine)
- Dr. Nancy Robitaille; Héma-Québec; Department of Pediatrics, CHU Sainte-Justine, Université de Montréal; Montréal, Québec, Canada (transfusion medicine, haematology)
- Dr. Renée Bazin; Medical Affairs and Innovation, Héma-Québec, Québec City, Québec, Canada (transfusion medicine, immunology)
- Dr. Nadine Shehata; Departments of Medicine, Laboratory Medicine and Pathobiology, Institute of Health Policy Management and Evaluation, University of Toronto; Division of Hematology, Mount Sinai Hospital; Canadian Blood Services, Toronto; Toronto, Ontario, Canada (transfusion medicine)
- Dr. Damon Scales; Department of Critical Care Medicine, Sunnybrook Health Sciences Centre; Department of Medicine, University of Toronto; Toronto, Ontario, Canada (critical care)
- Dr. Allison McGeer; Department of Microbiology, Sinai Health System; Department of Laboratory Medicine and Pathobiology and Dalla Lana School of Public Health, University of Toronto; Toronto, Ontario, Canada (infectious disease)
- Dr. Alexis Turgeon; Department of Anesthesiology and Critical Care Medicine, Division of Critical Care Medicine, Faculty of Medicine, Université Laval; CHU de Québec – Université Laval Research Centre, Population Health and Optimal Health Practices Research Unit, Trauma - Emergency - Critical Care Medicine, Université Laval; Québec City, Québec, Canada (critical care)
- Dr. Lisa Schwartz; Department of Health Research Methods, Evidence & Impact, McMaster University; Hamilton, Ontario, Canada (bioethics)
- Dr. Ryan Zarychanski; Department of Internal Medicine, University of Manitoba; Winnipeg, Manitoba, Canada (haematology, critical care)
- Dr. Nick Daneman; Department of Medicine, Sunnybrook Health Sciences Centre, University of Toronto; Toronto, Ontario, Canada (infectious disease, health policy)
- Dr. Luiz Amorim; Hemorio, Rio de Janeiro, Brazil (transfusion medicine, haematology)
- Dr. Bruce Sachais; New York Blood Center Enterprises, New York, NY; Department of Laboratory Medicine and Pathobiology, Weill Cornell Medical College; New York City, New York, USA (transfusion and apheresis medicine) Mr. Richard Carl (patient representative)
- Mr. Kent Cadogan Loftsgard (community advisor, specialty support to Recruitment Communications)

#### Executive committee

The Executive Committee (EC) oversees the day to day operations of the trial including monitoring trial progress, approving inter-institutional agreements, data management, and quality assurance. Members include the following: Dr. Donald Arnold, Dr. Philippe Bégin, Dr. Jeannie Callum, Prof Nancy Heddle, Dr. Alan Tinmouth, Dr. Michelle Zeller, Dr. Marshall Glesby, Dr. Melissa Cushing, and Dr. Richard Cook. Ad hoc members are Dr. Ryan Zarychanski, Dr. Nancy Robitaille, Dr. Dana Devine, and Dr. Alexis Turgeon.

#### Independent data safety monitoring committee (IDSMC)

The IDSMC includes members from both Canada and the USA. These individuals have expertise in transfusion medicine, infectious disease, cardiology, clinical trials, methodology, epidemiology, and biostatistics. An independent biostatistician will provide regular reports to the IDSMC. The IDSMC will monitor the safety and protection of human subjects participating in the trial; the proper conduct of the trial; and the ongoing scientific integrity, validity, and clinical and scientific relevance. The IDSMC will provide recommendations about continuing, modifying, and/or stopping the trial based on considerations of treatment outcomes, patient safety, and trial futility as appropriate.

#### Communications

As the structure of the CONCOR-1 trial evolved, an organizational chart (Fig. 4) was created to optimize flow of information to all relevant groups. Any protocol amendment will first require approval by the relevant national regulatory bodies, then the methods centre will be responsible for communicating protocol amendments to the study sites (in the form of new versions of the protocol and a summary of changes document) and collecting copies of the necessary local approvals prior to implementation.

#### Access to data

Study data may be made available to other investigators upon request. Such requests must first be approved by the Steering Committee.

#### Dissemination plans

The principal investigators will be responsible for publication of the data (manuscripts, abstracts, and posters, etc.) and are committed to publish and disseminate the study results in a timely manner without excessive restriction, regardless of whether the initial study hypothesis is confirmed or not. Investigators will not publish any data without approval from the Steering Committee. Medical writers will not be used. Authorship eligibility will comply with the International Committee of Medical Journal Editors criteria. The CONCOR-1 protocol has already been shared with US and European coalitions to allow investigators to collaborate on a core protocol and to share data. Results will be disseminated to patients and the public through the CONCOR-1 website (https://concor1.ca/), the media, and to various community groups identified by the study’s Community Advisory Committee members. Site investigators will also play a role in distributing results to patients and colleagues.

#### Ancillary and post-trial care

There is no specific ancillary or post-trial care related to the trial; however, patients will continue to receive standard of care for their underlying condition as per clinical practice. Insurance to cover the cost of compensation for participants who suffer harm from trial participation is available for Canadian and American participants.

## Discussion

Initial discussions to establish a Canadian trial of CCP began in late March 2020. The first CCP unit was collected 1 month later on April 24, 2020. The first patient was randomized on May 14, 2020. Rapid and intense coordination included the assembly of the Steering Committee with the range of required expertise, and coordination with numerous hospital sites and the blood suppliers. The organizational structure for the trial evolved quickly to define the roles of the methods centre, coordinating centres, and trial-specific working groups to allow for broader distribution of the trial workload to achieve trial launch at a rapid pace.

Given the context of the pandemic, there was no opportunity for a pilot study; nevertheless, we utilized a large, experienced and diverse Steering Committee to ensure as many challenges as possible will be avoided though strategic planning. Access to plasma donors was enhanced through public awareness campaigns, partnerships with public health, and an increasing number of recovered patients. To maximize access to the trial and enhance recruitment, we engaged approximately 50 Canadian centres, 3 hospitals in New York City (Weill Cornell Medicine), and centres in Brazil with a contingency plan to add more sites if needed. With this extensive recruitment plan, we hope to achieve our recruitment target within approximately 18 months. The eligibility criteria and outcomes in CONCOR-1 are harmonized with CCP trials in other countries. We will make our protocol publicly available through consortia in the USA and to allow investigators to share data and ensure completeness if local outbreaks taper off before sites are able to enroll their target sample size.

### Modeling supply and demand in an evolving pandemic

The success of the trial depends on both the supply of CCP from individuals who have recovered from COVID-19 and the demand from eligible patients with acute COVID-19 infection. Factors affecting supply include the number of recovered patients with adequate SARS-CoV-2 antibody titres, willingness of donors to provide CCP, validation of SARS-CoV-2 antibody testing (ELISA and neutralization assays), and turnaround time for antibody testing. Factors affecting patient recruitment are the number of new admissions for COVID-19 infections at participating sites, severity of COVID-19 infections for admitted patients, and speed of activation of study sites. Many of these factors were not known when the protocol was designed, but the investigators and Steering Committee prioritized the development of a rigorous protocol that could be implemented quickly. We modeled supply of CCP and demand of COVID-19 patients in Quebec based on information available at the time of protocol development. We partnered with hospitals in New York City and Brazil to maximize recruitment. In addition, our endpoint is harmonized with other CCP trials internationally.

We chose to study hospitalized patients with respiratory compromise within the first 12 days of respiratory symptom onset as this patient population appeared to experience the greatest benefit from convalescent plasma [9]. As passive immunization may be more effective early in the course of illness before high enough levels of antibodies are formed, patients with onset of respiratory symptoms more than 12 days from potential randomization are not eligible for participation. For patients with more advanced disease (e.g. critically ill requiring invasive mechanical ventilation), the benefits of CCP appear to be less evident [14, 37].

The primary outcome is the composite of intubation or death at day 30. Intubation is a clinically relevant outcome because of the high proportion of patients who require intubation (up to 40%) [14], the association with intubation and mortality, and the desire to demonstrate a reduction in healthcare utilization. By introducing mortality via the composite outcome, we address situations in which intubation cannot be offered due to patient or physician preference or lack of availability of ventilators. The pattern of intubation practices during the pandemic and the availability of ventilators and intensive care beds were assessed when the protocol was designed through an informal survey of intensive care physicians. We recognized that patterns of practice are dynamic and could evolve over the epidemic. Due to the uncertainty around the 30% baseline estimate of the event rate, we included an adaptive sample size recalculation mid-way through the trial so that the sample size can be adjusted if appropriate.

### Equitable CCP distribution

As part of the CONCOR-1 trial, we have established a platform for the distribution of CCP to ensure equity of plasma availability to hospitals and patients throughout Canada and the NYC sites while balancing optimal patient recruitment. This platform allows research staff to reserve an ABO-compatible unit for an eligible patient and release it back into inventory if the patient is randomized to standard of care. We developed a distribution model where CBS provides CCP to hub hospitals that distribute to other proximal sites. This CCP distribution platform is shared with other CCP trials in Canada for critically ill patients (REMAP-CAP: NCT02735707) and pediatrics (CONCOR-Kids: NCT04377568). The CCP distribution working group consists of representatives from all Canadian CCP trials, the blood suppliers, hospital transfusion services, a bioethicist, an operations researcher who developed the software application and a full-time research coordinator who will monitor CCP inventory daily. For sites in Québec, New York, and Brazil, the blood suppliers will distribute the CCP directly to the participating sites. Agreements between HQ and CBS will facilitate sharing of CCP for less common blood groups (B and AB).

The protocol was designed to mitigate risk to research and clinical staff during the pandemic by including remote processes for consent, data collection, and monitoring that do not rely on in-person contact. Data collection will be done through a daily review of the health record and telephone contact with the clinical team as required, and data monitoring will be done by remote access to health records until on-site monitoring is permitted. No additional procedures are required for the trial except for the CCP transfusion and a screening ABO blood group, which may be routine for all COVID-19 patients in certain study sites. Both study samples (baseline and 48 h after randomization) for viral load and antibody levels can be collected at the time of a pre-planned blood draw or from sample discards from the clinical laboratories.

Co-enrolment in drug trials is permitted. This will require collaboration with other site investigators to harmonize screening and consent procedures and establish trial prioritization. The rate of co-enrolment will be prospectively monitored by the IDSMC and the SC and the effect of co-enrolment will be evaluated in subgroup analyses. We anticipate that the rate of co-enrolment will be less than 20% since CONCOR-1 will recruit from many community hospitals where other COVID-19 trials are not open.

### Serology

**Fig. 5:**
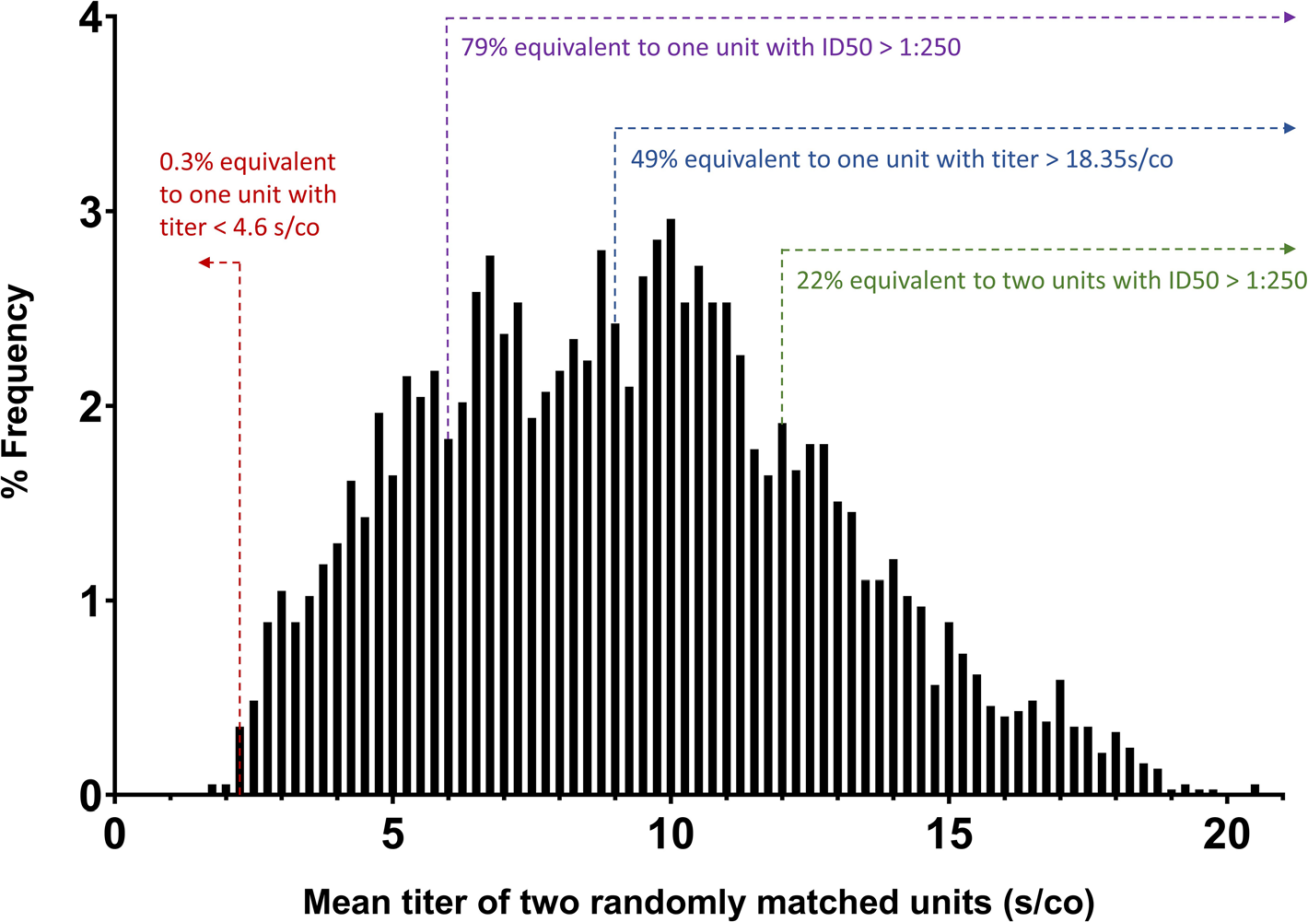
Expected distribution of transfused plasma potency. The distribution of mean antibody titre in transfused convalescent plasma units was projected by performing a simulation in which plasma units from different donors were paired randomly. The simulation is based on the titres of 1150 plasma units currently in inventory at one of the blood suppliers (Héma-Québec). Titers were originally established with an in-house ELISA but have been translated in equivalent d/co units from the ORTHO VITRIOS IgG assay

Antibody testing to qualify CCP products is done by ELISA, cyclic enhanced immunofluorescence assay, or viral neutralization assays. This strategy is aligned with FDA recommendations on the use of CCP [38] and accommodates the differences in the donor qualification requirements by the blood suppliers. As a secondary analysis, we will explore the relation between clinical outcomes and CCP anti-receptor-binding domain (RBD) and neutralizing antibody titres, which will be ultimately measured in all units. A previous non-randomized retrospective study reported that patient receiving a single unit of CCP with an anti-SARS-CoV2 titre greater than 18.35 s/co had lower mortality rate compared to those receiving one CCP unit with a titre lower than 4.6 s/co (Ortho VITROS IgG assay) (unpublished pre-print). Figure 5 shows how these dosages compare in terms of final amount of anti-SARS-CoV2 antibody transfused based on a simulation based of CCP collected to date for the trial at the HQ sites, and assuming transfusion of two units from randomly matched donors. Of note, two isolates of SARS-CoV-2 have been identified in Canada and the USA (wild type and D614G mutant) [39]. The D614G mutation involves the RBD, and there is debate as to whether this could affect infectivity or specificity of neutralization. Neutralization assays will therefore be performed for both isolates to allow comparison. These translational studies will inform future use of CCP.

### Community engagement

We established a Community Advisory Committee (CAC) to bring a diversity of perspectives based on relevant lived experiences and socio-demographic characteristics of COVID-19 patients, blood donors, and individuals with specific interests in the pandemic and its impact on the community (e.g. age, sex/gender, education, socioeconomic status, geographic location and race). Advisors from marginalized populations affected by the pandemic will help us understand and advocate for these groups such as residents of long-term care facilities or family members, individuals of colour, and Indigenous people [40, 41]. Some of the roles of this committee are (1) to maximize the relevance of the research for the community; (2) to advise on equitable distribution of CCP; (3) to enhance effective dissemination of study results; and (4) to provide feedback on analyses including awareness of the effects of social determinants [41, 42]. The CAC has seven members and plans to meet monthly.

In summary, the CONCOR-1 trial was designed rapidly as a collaborative effort by a team of experts in clinical trials in collaboration with three blood suppliers. This large randomized trial is adequately powered and sufficiently generalizable to address the efficacy and safety of CCP as potential treatment for COVID-19.

### Risks to the trial

A trial protocol designed to evaluate a new treatment during the ongoing pandemic is subject to risks. For one, enrolment is dependent on disease prevalence; second, the trial requires an adequate supply of plasma which had not been pre-planned; and third, estimates of event rate were based on available data at the outset of the pandemic and may be unstable. To mitigate these risks, we have activated the trial in a large number of centres throughout Canada including both academic and community hospitals, we have expanded into other countries, and we are pursuing methods to combine data with other similar trials without compromising trial integrity. CCP can be shared among the blood suppliers to supply the trial, and we have engaged with the blood suppliers to bolster recruitment of CCP donors. We have built-in a sample size re-estimation halfway through the trial.

#### Trial status

The trial has been approved by Health Canada, the FDA, and the National Research Ethics Commission (Comissão Nacional de Ética em Pesquisa) in Brazil. Research ethics board approvals have been received from all active sites and the blood suppliers. Trial recruitment started on May 8, 2020. There were 52 active sites (48 in Canada, 3 in New York City, 1 in Brazil; an Israeli site was planned but never activated). Over 2000 units of CCP were collected. On January 29, 2021, the IDSMC reviewed the data from the scheduled interim analysis (*n* = 614) and recommended that the trial be stopped for futility. On 29 January 2021, the trial was stopped after 940 patients were enrolled. The currently approved protocol version is 15 Jan 2021 v8.0.

## Supplementary Information

**Additional file 1.** Paper CRF package Version 4.0, 01 Oct 2020.

**Additional file 2.** Statistical Analysis Plan Version 2.0 19 Mar 2021.

**Additional file 3.** IDSMC Charter version 2.0, 03 Nov 2020.

**Additional file 4.** Clinical Trials Ontario Consent Form Template (English) Version 1.2, 13 Apr 2020 & Consent Form Template (French- for Quebec sites) Version 1.1, 12 Apr 2020.

## Data Availability

Study data may be made available to other investigators upon request. Such requests must first be approved by the Steering Committee. Requests should be sent to arnold@mcmaster.ca.

## Abbreviations

AE: Adverse event
CAC: Community Advisory Committee
CBS: Canadian Blood Services
CCP: COVID-19 convalescent plasma
CRF: Case report form
CTCAE: Common Terminology Criteria for Adverse Events
EC: Executive Committee
ECMO: Extracorporeal membrane oxygenation
eCRF: Electronic case report form
ELISA: Enzyme-linked immunosorbent assay
FDA: Food and Drug Administration
HQ: Héma-Québec
ICU: Intensive care unit
IDSMC: Independent Data Safety Monitoring Committee
MedDRA: Medical Dictionary for Regulatory Activities
MERS: Middle East respiratory syndrome
NYBC: New York Blood Center
OR: Odds ratio
RBD: Receptor-binding domain
RCT: Randomized controlled trial
REDCap: Research Electronic Data Capture
SAE: Serious adverse event
SARS: Severe acute respiratory syndrome
SC: Steering Committee
SUSAR: Suspected unexpected serious adverse reaction
TACO: Transfusion-associated circulatory overload
TRALI: Transfusion-related acute lung injury
