# Supplementary material for: Convalescent plasma for adults with acute COVID-19 respiratory illness (CONCOR-1): study protocol for an international, multicentre, randomized, open-label trial
Source: Trials. 2021 May 4;22:323. doi: 10.1186/s13063-021-05235-3 (PMC8094980; doi:10.1186/s13063-021-05235-3)
Supplement: Supplementary file 1 — Additional file 1. Paper CRF package Version 4.0, 01 Oct 2020. [file 13063_2021_5235_MOESM1_ESM.pdf]

CONCOR-1 Study:
Source Data Worksheets

Study #: _ _ _ _ - _ _ _

Name	Signature	Date

Name	Signature	Date

[CONCOR-1 CONValescent Plasma for Acute COVID-19 Patients]

A Randomized Open-Label Trial of CONvalescent Plasma for Hospitalized Adults With Acute COVID-19 Respiratory Illness (CONCOR-1)

Study #: _ _ _ _ - _ _ _

REDCap Section: SCREENING

DO NOT ENTER ANY DATA IN REDCAP UNTIL YOU ARE READY TO RANDOMIZE!

Informed Consent/Assent

<table>
<tr><td>1. Was the patient willing to give consent?</td><td>☐ Yes – Written Informed Consent</td><td colspan="2">☐ Yes – Telephone Consent</td></tr>
<tr><td colspan="2">2. Date and time of Informed Consent (dd.mm.yyyy; HH:MM):</td><td colspan="2">_ _ / _ _ / _ _ _ _   _ _ : _ _</td></tr>
<tr><td>3. Who was the consent obtained from?</td><td>☐ Patient</td><td>☐ Legally Authorized Representative</td><td>☐ Other ____________________</td></tr>
<tr><td>4. Was the participant less than 18?
☐ Yes   ☐ No</td><td>5. Protocol Version:
________________</td><td>6. Consent Version:
________________</td><td>7. Who obtained consent?
________________________</td></tr>
<tr><td colspan="4">REMINDER: Written consent may be required in the future. Check local requirements.</td></tr>
</table>

Eligibility

Screening number (from site screening log): ____________ Date of screening (dd/mm/yyyy): _ _ / _ _ / _ _ _ _

Date of Respiratory Symptom Onset (dd/mm/yyyy): _ _ / _ _ / _ _ _ _

Inclusion Criteria

INCLUSION CRITERIA

1. Age ≥ 16 years of age — Yes ☐ No ☐
2. Admitted to hospital with confirmed COVID-19 respiratory illness — Yes ☐ No ☐
3. Requires supplemental oxygen — Yes ☐ No ☐
4. 500 mL of ABO compatible plasma is available (please check "Project Bookmarks" for the inventory information) — Yes ☐ No ☐

EXCLUSION CRITERIA

1. Intubated or plan in place for intubation — Yes ☐ No ☐
2. Plasma is contraindicated (e.g., history of anaphylaxis from transfusion) — Yes ☐ No ☐
3. Decision in place for no active treatment — Yes ☐ No ☐
4. Onset of respiratory signs or symptoms > 12 days prior to randomization — Yes ☐ No ☐

This patient is: ☐ Eligible ☐ Ineligible

Please have your site investigator sign off on eligibility in REDCap prior to randomization

Randomization

**1.** Patient meets eligibility criteria?<br>☐ Yes ☐ No	**2.** Date of Randomization *(dd.mm.yyyy):*<br>_ _ / _ _ / _ _ _ _		
**3.** Number of days (at randomization) since symptom onset [*if > 12, patient is a SCREEN FAIL]: ____________	**4.** Age: ___________	**5.** Age group: ☐ <60 ☐ ≥ 60	
**RANDOMIZATION: Please confirm with the hub blood bank for the availability of 500 mL ABO compatible convalescent plasma before randomization**			
**6.** Randomization time (HH:MM): __________	**7**. Randomized to: ☐ Convalescent Plasma ☐ Standard of Care		**8.** Randomized by:<br>_______________

EQ-5D-5L (QoL Questionnaire) – Baseline *FILL THIS OUT AS YOUR SOURCE DOCUMENT

We are trying to find out what you think about your health. I will first ask you some simple questions about your health TODAY. I will then ask you to rate your health on a measuring scale. I will explain what to do as I go along but please interrupt me if you do not understand something or if things are not clear to you. Please also remember that there are no right or wrong answers. We are interested here only in your personal view.

First I am going to read out some questions. Each question has a choice of five answers. Please tell me which answer best describes your health TODAY. Do not choose more than one answer in each group of questions.

☐ The participant is not capable of responding

Date of survey (dd/mm/yyyy): _ _ / _ _ / _ _ _ _

Please tick ONE box that describes your health TODAY:

1. Mobility
    - You have no problems in walking about
    - You have slight problems in walking about
    - You have moderate problems in walking about
    - You have severe problems in walking about
    - You am unable to walk about

Please tick ONE box that describes your health TODAY:

2. Self-Care
    - You have no problems washing or dressing myself
    - You have slight problems washing or dressing myself
    - You have moderate problems washing or dressing myself
    - You have severe problems washing or dressing myself
    - You am unable to wash or dress myself

Please tick ONE box that describes your health TODAY:

3. Usual Activities (e.g., work, study, housework, family or leisure activities)
    - You have no problems doing my usual activities
    - You have slight problems doing my usual activities
    - You have moderate problems doing my usual activities
    - You have severe problems doing my usual activities
    - You am unable to do my usual activities

Please tick ONE box that describes your health TODAY:

4. Pain/Discomfort:
    - You have no pain or discomfort
    - You have slight pain or discomfort
    - You have moderate pain or discomfort
    - You have severe pain or discomfort
    - You have extreme pain or discomfort

Please tick ONE box that describes your health TODAY:

5. Anxiety/Depression:
    - You am not anxious or depressed
    - You am slightly anxious or depressed
    - You am moderately anxious or depressed
    - You am severely anxious or depressed
    - You am extremely anxious or depressed

REDCap Section: Baseline (Day 1)

CCP Administration

Date of 1st unit (DD/MM/YYYY) _ _ / _ _ / _ _ _ _<br><br>Start Time: _ _ : _ _ End Time: _ _ : _ _<br><br>Total transfusion volume administered (mL):<br>________	Unit Number:<br>_______________<br>Product Code:<br>______________<br>ABO<br>Group:_________________	○ 1 unit of 500cc<br>○ 1 unit of 250cc
Was the transfusion interrupted? ○ Yes ○ No<br>Total length of interruption (in minutes):<br>_______________<br>If interrupted, reason for interruption:<br>○ Adverse transfusion reaction (complete AE form)<br>○ Other reason<br>_________________________________	Was the transfusion restarted?<br>○ Yes ○ No ○ N/A	
Date of 2nd unit (DD/MM/YYYY) _ _ / _ _ / _ _ _ _<br>Start Time: _ _ : _ _ End Time: _ _ : _ _<br>○ N/A<br>Total transfusion volume administered (mL):<br>_________	Unit Number:<br>_______________<br>Product Code:<br>______________<br>ABO Group:<br>_________________	○ 1 unit of<br>250cc
Was the transfusion interrupted? ○ Yes ○ No<br>Total length of interruption (in minutes):<br>_______________<br>If interrupted, reason for interruption:<br>○ Adverse transfusion reaction (complete AE form)<br>○ Other reason<br>_________________________________	Was the transfusion restarted?<br>○ Yes ○ No ○ N/A	

Blood Sample Collection for Viral Load/Antibody testing (REDCap Section: Baseline Day 1 and Day 2)

Sample #1: Pre-transfusion

1. Was a sample collected for the 1st sample (within 48 hours prior to randomization): ☐ Yes ☐ No
   If not collected, reason? ___________________________
2. Date and time blood sample collected from patient (DD/MM/YYYY HH:MM): _ _ / _ _ / _ _ _ _ _ _ : _ _
3. Date and time research sample processed (DD/MM/YYYY HH:MM): _ _ / _ _ / _ _ _ _ _ _ _ : _ _
4. Sample ID number (please make sure you have entered this into REDCap within one week): ____________
5. a. Type of sample: ☐ Serum ☐ Plasma
   b. Type of anticoagulant: ☐ EDTA ☐ Citrate ☐ Lithium ☐Heparin ☐ NaF
6. Number of cryovials collected: ☐ 1 ☐ 2
7. Volume per tube:
   a. Tube 1: _______________
   b. Tube 2: _______________

Sample #2: Post-transfusion

1. Was a sample collected for the 2nd sample (closest to 48 hours but within 24 hrs – 5 days after randomization):
   ☐ Yes ☐ No
   If not collected, reason? ___________________________
2. Date and time blood sample collected from patient (DD/MM/YYYY HH:MM): _ _ / _ _ / _ _ _ _ __:__
3. Date and time research sample processed (DD/MM/YYYY HH:MM): _ _ / _ _ / _ _ _ _ __:__
4. Sample ID number (please make sure you have entered this into REDCap within one week): ____________
5. a. Type of sample: ☐ Serum ☐ Plasma
   b. Type of anticoagulant: ☐ EDTA ☐ Citrate ☐ Lithium ☐Heparin ☐ NaF
6. Number of cryovials collected: ☐ 1 ☐ 2
7. Volume per tube:
   a. Tube 1: _______________
   b. Tube 2: _______________

***if sample was not collected, record as a minor protocol deviation in your on-site log**

Hospitalization Assessment - Baseline (Day 1) to Day 30/Discharge (whichever comes first)

*Vital Signs (BASELINE ONLY) *use most recent value prior to time of randomization*

1. Blood Pressure Systolic (mmHg): _______________
2. Blood Pressure Diastolic (mmHg): ______________
3. Pulse (beats per minute): _______________________
4. Temperature (Celsius): _______________________

COVID Testing

1. Method of testing: ☐ Nasal Swab ☐ Throat Swab ☐ Sputum ☐ Bronchoalveolar lavage
   ☐ Nasopharyngeal Swab ☐ Not known ☐ Other ______________
2. Date of positive COVID-19 test (DD/MM/YYYY): _ _ / _ _ / _ _ _ _

Participant Health Information:

1. Sex: ☐ Male ☐ Female
2. If female, is the patient pregnant? ☐ Yes ☐ No
   a. If pregnant, estimated date of delivery (DD/MM/YYYY): _ _ / _ _ / _ _ _ _

   If patient is pregnant, please enter a reminder into the Calendar application for one week after estimated due date – "Call participant and complete pregnancy outcomes form"
3. Date and time of admission to hospital (DD/MM/YYYY HH:MM): _ _ / _ _ / _ _ _ _ _ _ : _ _
4. Participant location at time of enrollment: ☐ ER ☐ Ward ☐ ICU
5. Participant month/year of birth (MM/YYYY): _ _ / _ _ _ _
6. Ethnicity (check all that apply): ☐ American Indian ☐ Alaskan Native ☐ Asian
   ☐ White ☐ Hispanic or Latino ☐ Native Hawaiian/Other Pacific Islander
   ☐ Black or African American/Canadian ☐ Other: __________________ ☐ Not known
7. ABO Group: ☐ O ☐ A ☐ B ☐ AB
8. Height (cm): ________
9. Weight (kg): ________
10. Medical History:
    ☐ Diabetes ☐ Hypertension ☐ Ischemic Heart Disease ☐ Stroke ☐ Atrial Fibrillation
    ☐ Congestive Heart Failure ☐ Chronic Kidney Disease ☐ Cirrhosis/liver disease ☐ Asthma
    ☐ COPD ☐ Obstructive Sleep Apnea ☐ Active Cancer ☐ Dementia ☐ Coagulation Disorder
    ☐ History of Solid Organ Transplant ☐ Immunosuppression ☐ Other: _________________________
11. Smoking status: ☐ Never Smoker ☐ Former Smoker ☐ Current Smoker
    If former or current smoker, how many years did the participate smoke? _______
    If former or current smoker, how many cigarettes does the participate smoke daily? _______
    If former smoker, year ceased smoking: _______
12. Does the participant vape? ☐ Yes ☐ No
    If yes, how often does the participant vape? ☐ Daily ☐ Weekly ☐ Monthly
    How many times does the participant vape in the time frame specified above? _________
13. Does the participant smoke cannabis? ☐ Yes ☐ No
    If yes, how often does the participant smoke cannabis? ☐ Daily ☐ Weekly ☐ Monthly
    How many times does the participant smoke cannabis in the time frame specified above? __________

14. COVID-19 Symptoms at baseline:
☐ Fever ☐ Cough ☐ Shortness of breath ☐ Myalgia/Arthralgia ☐ Headache ☐ Anosmia (loss of taste/smell) ☐ Diarrhea ☐ Vomiting ☐ Fatigue ☐ Other
Other symptoms: ____
____
15. Date of initial onset of *any* symptoms (this may be different from the initial onset of *respiratory* symptoms) (dd/mm/yyyy) : _ _ / _ _ / _ _ _ _
16. Currently taking relevant medication for non COVID-19 reasons (last 7 days):
☐ ACE Inhibitor ☐ ACE receptor blocker (ARB) ☐ Non-steroidal anti-inflammatory drugs (NSAIDs)
☐ Colchicine ☐ Systemic corticosteroids ☐ Inhaled corticosteroids ☐ Immunomodulatory agents
☐ Anticoagulants [☐ Direct oral anticoagulant; ☐ Unfractionated Heparin; ☐ Low Molecular Weight Heparin; ☐ Warfarin]

17. All medication administered to treat COVID-19 before randomization:

**1.** Antibiotics<br>☐ Azithromycin (Zithromax, Z-pack)<br>☐ Other antibiotic, specify: _______________	**2.** Anti-inflammatory/immunomodulatory – check all below<br>☐ Tocilizumab ☐ Systemic corticosteroids<br>☐ Colchicine ☐ Inhaled corticosteroids<br>☐ Interferon ☐ Other anti-inflammatory: ____________
**3.** Anti-viral – check all below<br>☐ Chloroquine/hydroxychloroquine<br>☐ Lopinavir-ritonavir<br>☐ Oseltamivir ☐ Remdesivir<br>☐ Other antiviral: _______________________	**4.** Anticoagulants – check all below<br>☐ Direct oral anticoagulant<br>☐ Unfractionated Heparin<br>☐ Low Molecular Weight Heparin<br>☐ Warfarin<br>☐ Other anticoagulant: _______________________
**5.** Other COVID relevant medications:_________________	4b. If DOAC, UFH, LMWH, or other:<br>Dose: ☐ prophylactic ☐therapeutic

16a. Were any medications administered as part of another research study? ☐ Yes ☐ No
i. Name of medication(s): ____________________________ or
Study arm: ______________ or ☐UNKNOWN—blinded study
ii. Name of study or studies:____________________________

Pleaseenter all COVID-19 relevant medications the patient was on at baseline or started after randomization in the MEDICATION LOG

18. Was medical imaging done (CT Chest and/or Chest Xray closest to randomization)? ☐ Yes ☐ No

**Medical Image 1:**	Date (DD/MM/YYYY): _ _ / _ _ / _ _ _ _	**Medical Image 2:**	Date (DD/MM/YYYY): _ _ / _ _ / _ _ _ _
**1.** Type<br>☐ CT Chest<br>☐ Chest Xray	**2.** Result<br>☐ Normal<br>☐ Patchy Shadowing<br>☐ Infiltrates<br>☐ Other Abnormal	**1.** Type<br>☐ CT Chest<br>☐ Chest Xray	**2.** Result<br>☐ Normal<br>☐ Patchy Shadowing<br>☐ Infiltrates<br>☐ Other Abnormal

If 'other abnormal', specify: ____

REDCap Section: Baseline (Day 1) to Day 7, and Day 14

Laboratory and Blood Gas Assessments

Please take last value of the day, if test not done write 'NA'. ***If your site uses different units, please make the appropriate conversions.*** *Ensure you are entering in the units stated in the CRF.*
**Lab values for Day 1 should be the last value PRIOR to randomization for both the standard of care and control arms*

Date:	1*	2	3	4	5	6	7	14 (+/- 3 days)
RBC ($x10^{12}$/L)								
WBC ($x10^{9}$/L)								
HB (g/L)								
PLT ($x10^{9}$/L)								
Neut ($x10^{9}$/L)								
Lymph ($x10^{9}$/L)								
Mono ($x10^{9}$/L)								
Eosino ($x10^{9}$/L)								
Baso ($x10^{9}$/L)								
INR								
aPTT (seconds)								
Fibrinogen (g/L)								
D-dimer (ug/L)								
ALT (U/L)								
ALP (U/L)								
LDH (U/L)								
Bilirubin (umol/L)								
Creatinine (umol/L)								
HS Troponin (ng/L)								
Ferritin (g/L)								
Albumin (g/L)								
C-Reactive Protein (mg/L)								
Total Protein (g/L)								
Lactate (mmol/L)								
Total CO_2 (mmol/L)								

Study #: ____-___

REDCap Section: Baseline (Day 1) to Day 30/Discharge

Oxygen Requirements and Parameters - *If not available, write 'NA'*.
Please note that blood gases are only to be collected for Day 1-7 and Day 14 (+/- 3 days).
**Values for Day 1 should be the worst value of the day PRIOR to randomization for both the standard of care and control arms*

Date:	1*	2	3	4	5	6	7	8	9	10
**Oxygen requirement** HFNC – high flow nasal cannula NIPPV – non-invasive positive pressure ventilation	☐ Nasal Prongs ☐ Oxygen masks ☐ HFNC ☐ NIPPV ☐ None ☐ Intubated	☐ Nasal Prongs ☐ Oxygen masks ☐ HFNC ☐ NIPPV ☐ None ☐ Intubated	☐ Nasal Prongs ☐ Oxygen masks ☐ HFNC ☐ NIPPV ☐ None ☐ Intubated	☐ Nasal Prongs ☐ Oxygen masks ☐ HFNC ☐ NIPPV ☐ None ☐ Intubated	☐ Nasal Prongs ☐ Oxygen masks ☐ HFNC ☐ NIPPV ☐ None ☐ Intubated	☐ Nasal Prongs ☐ Oxygen masks ☐ HFNC ☐ NIPPV ☐ None ☐ Intubated	☐ Nasal Prongs ☐ Oxygen masks ☐ HFNC ☐ NIPPV ☐ None ☐ Intubated	☐ Nasal Prongs ☐ Oxygen masks ☐ HFNC ☐ NIPPV ☐ None ☐ Intubated	☐ Nasal Prongs ☐ Oxygen masks ☐ HFNC ☐ NIPPV ☐ None ☐ Intubated	☐ Nasal Prongs ☐ Oxygen masks ☐ HFNC ☐ NIPPV ☐ None ☐ Intubated
**Prone positioning:**	☐ Yes ☐ No ☐ Unknown	☐ Yes ☐ No ☐ Unknown	☐ Yes ☐ No ☐ Unknown	☐ Yes ☐ No ☐ Unknown	☐ Yes ☐ No ☐ Unknown	☐ Yes ☐ No ☐ Unknown	☐ Yes ☐ No ☐ Unknown	☐ Yes ☐ No ☐ Unknown	☐ Yes ☐ No ☐ Unknown	☐ Yes ☐ No ☐ Unknown
**SpO_2 (%)** (worst value at time of worst FiO_2)										
**FiO_2* (%)** (worst value of the day)										
**Type of blood gas:**	☐ ABG ☐ VBG ☐ CBG ☐ None	☐ ABG ☐ VBG ☐ CBG ☐ None	☐ ABG ☐ VBG ☐ CBG ☐ None	☐ ABG ☐ VBG ☐ CBG ☐ None	☐ ABG ☐ VBG ☐ CBG ☐ None	☐ ABG ☐ VBG ☐ CBG ☐ None	☐ ABG ☐ VBG ☐ CBG ☐ None			
**PaO_2 result [mmHg]** closest to time of worst FiO_2)										
**PCO_2 result [mmHg]** (from same blood gas record on PaO_2)										

***Please use the following table to convert L/min to %: https://www.intensive.org/epic2/Documents/Estimation%20of%20PO2%20and%20FiO2.pdf**

Study #: _ _ _ _ - _ _ _

Date:	11	12	13	14	15	16	17	18	19	20
**Oxygen requirement** HFNC – high flow nasal cannula NIPPV – non-invasive positive pressure ventilation	☐ Nasal Prongs ☐ Oxygen masks ☐ HFNC ☐ NIPPV ☐ Intubated ☐ None	☐ Nasal Prongs ☐ Oxygen masks ☐ HFNC ☐ NIPPV ☐ Intubated ☐ None	☐ Nasal Prongs ☐ Oxygen masks ☐ HFNC ☐ NIPPV ☐ Intubated ☐ Non	☐ Nasal Prongs ☐ Oxygen masks ☐ HFNC ☐ NIPPV ☐ Intubated ☐ None	☐ Nasal Prongs ☐ Oxygen masks ☐ HFNC ☐ NIPPV ☐ Intubated ☐ None	☐ Nasal Prongs ☐ Oxygen masks ☐ HFNC ☐ NIPPV ☐ Intubated ☐ None	☐ Nasal Prongs ☐ Oxygen masks ☐ HFNC ☐ NIPPV ☐ Intubated ☐ None	☐ Nasal Prongs ☐ Oxygen masks ☐ HFNC ☐ NIPPV ☐ Intubated ☐ None	☐ Nasal Prongs ☐ Oxygen masks ☐ HFNC ☐ NIPPV ☐ Intubated ☐ None	☐ Nasal Prongs ☐ Oxygen masks ☐ HFNC ☐ NIPPV ☐ Intubated ☐ None
**Prone positioning:**	☐ Yes ☐ No ☐ Unknown	☐ Yes ☐ No ☐ Unknown	☐ Yes ☐ No ☐ Unknown	☐ Yes ☐ No ☐ Unknown	☐ Yes ☐ No ☐ Unknown	☐ Yes ☐ No ☐ Unknown	☐ Yes ☐ No ☐ Unknown	☐ Yes ☐ No ☐ Unknown	☐ Yes ☐ No ☐ Unknown	☐ Yes ☐ No ☐ Unknown
**SpO_2 (%)** (worst value at time of worst FiO_2)										
**FiO_2* (%)** (worst value of the day)										
**Type of blood gas:**				☐ ABG ☐ VBG ☐ CBG ☐ None						
**PaO_2 result [mmHg]** (closest to time of worst FiO_2)										
**PCO_2 result [mmHg]** (from same blood gas record on PaO_2)										

***Please use the following table to convert L/min to %: https://www.intensive.org/epic2/Documents/Estimation%20of%20PO2%20and%20FiO2.pdf**

Date:	21	22	23	24	25	26	27	28	29	30
**Oxygen requirement**<br>HFNC – high flow nasal cannula<br>NIPPV – non-invasive positive pressure ventilation	☐ Nasal Prongs<br>☐ Oxygen masks<br>☐ HFNC<br>☐ NIPPV<br>☐ Intubated<br>☐ None	☐ Nasal Prongs<br>☐ Oxygen masks<br>☐ HFNC<br>☐ NIPPV<br>☐ Intubated<br>☐ None	☐ Nasal Prongs<br>☐ Oxygen masks<br>☐ HFNC<br>☐ NIPPV<br>☐ Intubated<br>☐ None	☐ Nasal Prongs<br>☐ Oxygen masks<br>☐ HFNC<br>☐ NIPPV<br>☐ Intubated<br>☐ None	☐ Nasal Prongs<br>☐ Oxygen masks<br>☐ HFNC<br>☐ NIPPV<br>☐ Intubated<br>☐ None	☐ Nasal Prongs<br>☐ Oxygen masks<br>☐ HFNC<br>☐ NIPPV<br>☐ Intubated<br>☐ None	☐ Nasal Prongs<br>☐ Oxygen masks<br>☐ HFNC<br>☐ NIPPV<br>☐ Intubated<br>☐ None	☐ Nasal Prongs<br>☐ Oxygen masks<br>☐ HFNC<br>☐ NIPPV<br>☐ Intubated<br>☐ None	☐ Nasal Prongs<br>☐ Oxygen masks<br>☐ HFNC<br>☐ NIPPV<br>☐ Intubated<br>☐ None	☐ Nasal Prongs<br>☐ Oxygen masks<br>☐ HFNC<br>☐ NIPPV<br>☐ Intubated<br>☐ None
**Prone positioning:**	☐ Yes<br>☐ No<br>☐ Unknown	☐ Yes<br>☐ No<br>☐ Unknown	☐ Yes<br>☐ No<br>☐ Unknown	☐ Yes<br>☐ No<br>☐ Unknown	☐ Yes<br>☐ No<br>☐ Unknown	☐ Yes<br>☐ No<br>☐ Unknown	☐ Yes<br>☐ No<br>☐ Unknown	☐ Yes<br>☐ No<br>☐ Unknown	☐ Yes<br>☐ No<br>☐ Unknown	☐ Yes<br>☐ No<br>☐ Unknown
**SpO_2 (%)**<br>(worst value at time of worst FiO_2)										
**FiO_2* (%)**<br>(worst value of the day)										

***Please use the following table to convert L/min to %: https://www.intensive.org/epic2/Documents/Estimation%20of%20PO2%20and%20FiO2.pdf**

Additional Study Outcomes of Interest – enter into REDCap Section: Day 30

Date:	1	2	3	4	5	6	7	8	9	10
ECMO	☐ Yes<br>☐ No	☐ Yes<br>☐ No	☐ Yes<br>☐ No	☐ Yes<br>☐ No	☐ Yes<br>☐ No	☐ Yes<br>☐ No	☐ Yes<br>☐ No	☐ Yes<br>☐ No	☐ Yes<br>☐ No	☐ Yes<br>☐ No
Renal Replacement	☐ Yes<br>☐ No	☐ Yes<br>☐ No	☐ Yes<br>☐ No	☐ Yes<br>☐ No	☐ Yes<br>☐ No	☐ Yes<br>☐ No	☐ Yes<br>☐ No	☐ Yes<br>☐ No	☐ Yes<br>☐ No	☐ Yes<br>☐ No
Myocarditis	☐ Yes<br>☐ No	☐ Yes<br>☐ No	☐ Yes<br>☐ No	☐ Yes<br>☐ No	☐ Yes<br>☐ No	☐ Yes<br>☐ No	☐ Yes<br>☐ No	☐ Yes<br>☐ No	☐ Yes<br>☐ No	☐ Yes<br>☐ No
Date:	11	12	13	14	15	16	17	18	19	20
ECMO	☐ Yes<br>☐ No	☐ Yes<br>☐ No	☐ Yes<br>☐ No	☐ Yes<br>☐ No	☐ Yes<br>☐ No	☐ Yes<br>☐ No	☐ Yes<br>☐ No	☐ Yes<br>☐ No	☐ Yes<br>☐ No	☐ Yes<br>☐ No
Renal Replacement	☐ Yes<br>☐ No	☐ Yes<br>☐ No	☐ Yes<br>☐ No	☐ Yes<br>☐ No	☐ Yes<br>☐ No	☐ Yes<br>☐ No	☐ Yes<br>☐ No	☐ Yes<br>☐ No	☐ Yes<br>☐ No	☐ Yes<br>☐ No
Myocarditis	☐ Yes<br>☐ No	☐ Yes<br>☐ No	☐ Yes<br>☐ No	☐ Yes<br>☐ No	☐ Yes<br>☐ No	☐ Yes<br>☐ No	☐ Yes<br>☐ No	☐ Yes<br>☐ No	☐ Yes<br>☐ No	☐ Yes<br>☐ No
Date:	21	22	23	24	25	26	27	28	29	30
ECMO	☐ Yes<br>☐ No	☐ Yes<br>☐ No	☐ Yes<br>☐ No	☐ Yes<br>☐ No	☐ Yes<br>☐ No	☐ Yes<br>☐ No	☐ Yes<br>☐ No	☐ Yes<br>☐ No	☐ Yes<br>☐ No	☐ Yes<br>☐ No
Renal Replacement	☐ Yes<br>☐ No	☐ Yes<br>☐ No	☐ Yes<br>☐ No	☐ Yes<br>☐ No	☐ Yes<br>☐ No	☐ Yes<br>☐ No	☐ Yes<br>☐ No	☐ Yes<br>☐ No	☐ Yes<br>☐ No	☐ Yes<br>☐ No
Myocarditis	☐ Yes<br>☐ No	☐ Yes<br>☐ No	☐ Yes<br>☐ No	☐ Yes<br>☐ No	☐ Yes<br>☐ No	☐ Yes<br>☐ No	☐ Yes<br>☐ No	☐ Yes<br>☐ No	☐ Yes<br>☐ No	☐ Yes<br>☐ No

REDCap Section: Day 30

Day 30 – Phone Call/Chart Review

1. Date of Day 30 assessment (DD/MM/YYYY): _ _ / _ _ / _ _ _ _
2. Were there any adverse events at Day 30 follow-up? If so, entered in AE log? ☐ Yes ☐ No
3. Were there any NEW COVID-19 medications at Day 30 follow-up? If so, entered in the medication log?
   ☐ Yes ☐ No
4. Was medical imaging done since randomization up to Day 30 follow-up? If so, filled out medical imaging form?
   ☐ Yes ☐ No
5. Was there any other blood products (blood components or protein plasma products) transfused since randomization to Day 30 follow-up? If so, filled out transfusion log? ☐ Yes ☐ No
6. Were there any major protocol deviations? If so, filled out in Major Deviation Form in REDCap? ☐ Yes ☐ No
7. Status of patient: ☐ Died ☐ Discharged ☐ Still hospitalized ☐ Withdrew consent ☐ Lost to follow-up
   a. If patient died, date of death (DD/MM/YYYY): _ _ / _ _ / _ _ _ _
   b. If patient was discharged:
      i. Date of discharge (DD/MM/YYYY): _ _ / _ _ /_ _ _ _
      ii. Discharged to:
         ☐ Home ☐ Transfer to another facility ☐ Palliative discharge ☐ Other ______________
      iii. Re-admitted for COVID-19? ☐ Yes ☐ No
         Date of re-admission (DD/MM/YYYY): _ _ / _ _ / _ _ _ _
         Was intensive care unit (ICU) level required? ☐ Yes ☐ No
            If yes, provide the length of stay (days): _______
         Date of discharge from re-admission (DD/MM/YYYY): _ _ / _ _ / _ _ _ _ ☐ N/A Still Admitted
      iv. Re-admitted for non COVID-19? ☐ Yes ☐ No
            If yes, reason: _____________________________
   c. If lost to follow-up, provide reason, date of last contact, other details: ________________________________
   ____
8. Was the patient ever admitted to the ICU? ☐ Yes ☐ No
   a. If yes, admission date (DD/MM/YYYY): _ _ / _ _ / _ _ _ _
   b. Length of stay (days):_______
   c. Patient still in ICU at day 30? ☐ Yes ☐ No
9. Was the patient ever intubated? ☐ Yes ☐ No
   a. Intubation date(s) and time(s) (DD/MM/YYYY HH:MM):
      i. _ _ / _ _ / _ _ _ _ _ _ : _ _ ii. _ _ / _ _ / _ _ _ _ _ _ : _ _ iii. _ _ / _ _ / _ _ _ _ _ _ : _ _
10. Was the patient ever extubated? ☐ Yes ☐ No
   a. Extubation date(s) and time (s) (DD/MM/YYYY HH:MM):
      i. _ _ / _ _ / _ _ _ _ _ _ : _ _ ii. _ _ / _ _ / _ _ _ _ _ _ : _ _ iii. _ _ / _ _ / _ _ _ _ _ _ : _ _

10. Was the patient co-enrolled in any other trial at any point during the study (including already enrolled at baseline)? ☐ Yes ☐ No
   a. Name of trial(s): ____
   b. Arm of trial(s): ____
   *If any medications were administered as part of the trial, please complete the medication form in the ‘as needed’ section

EQ-5D-5L (QoL Questionnaire) – at 30 days *USE THIS AS THE SOURCE DOCUMENT

We are trying to find out what you think about your health. I will first ask you some simple questions about your health TODAY. I will then ask you to rate your health on a measuring scale. I will explain what to do as I go along but please interrupt me if you do not understand something or if things are not clear to you. Please also remember that there are no right or wrong answers. We are interested here only in your personal view.

First I am going to read out some questions. Each question has a choice of five answers. Please tell me which answer best describes your health TODAY. Do not choose more than one answer in each group of questions.

☐ The participant is not capable of responding

Date of survey (dd/mm/yyyy): _ _ / _ _ / _ _ _ _

Please tick ONE box that describes your health TODAY:

1. Mobility
    - You have no problems in walking about
    - You have slight problems in walking about
    - You have moderate problems in walking about
    - You have severe problems in walking about
    - You am unable to walk about

Please tick ONE box that describes your health TODAY:

2. Self-Care
    - You have no problems washing or dressing myself
    - You have slight problems washing or dressing myself
    - You have moderate problems washing or dressing myself
    - You have severe problems washing or dressing myself
    - You am unable to wash or dress myself

Please tick ONE box that describes your health TODAY:

3. Usual Activities (e.g., work, study, housework, family or leisure activities)
    - You have no problems doing my usual activities
    - You have slight problems doing my usual activities
    - You have moderate problems doing my usual activities
    - You have severe problems doing my usual activities
    - You am unable to od my usual activities

Please tick ONE box that describes your health TODAY:

4. Pain/Discomfort:
    - You have no pain or discomfort
    - You have slight pain or discomfort
    - You have moderate pain or discomfort
    - You have severe pain or discomfort
    - You have extreme pain or discomfort

Please tick ONE box that describes your health TODAY:

5. Anxiety/Depression:
    - You am not anxious or depressed
    - You am slightly anxious or depressed
    - You am moderately anxious or depressed
    - You am severely anxious or depressed
    - You am extremely anxious or depressed

REDCap Section: Day 90

90 Day Status (for patients still in hospital at Day 30) – no call required, just vital status between Day 30–90 follow-up

1. Was the patient still hospitalized at Day 30 (If 'No', skip to 'Investigator Review')? ☐ Yes ☐ No
2. Date of Day 90 follow up (DD/MM/YYYY): _ _ / _ _ / _ _ _ _
3. What is the status at Day 90 for this participant: ☐ Died ☐ Discharged ☐ Hospitalized
   If participant died, date participant died (DD/MM/YYYY): _ _ /_ _ / _ _ _ _
   If participant was discharged, date of discharge (DD/MM/YYYY): _ _ / _ _ / _ _ _ _

Investigator Review

1. Date of review (DD/MM/YYYY): _ _ / _ _ / _ _ _ _
2. I have reviewed the records for this participant and confirm they are correct and complete: ☐ Yes ☐ No

Please have your investigator e-sign and lock the record in REDCap

REDCap Section: As Needed

COVID-19 Relevant Treatment Medication Log (use as many as needed)

**Medication Name:** ____	Medication start date (DD/MM/YYYY): _ _ / _ _ / _ _ _ _<br>Medication stop date (DD/MM/YYYY): _ _ / _ _ / _ _ _ _
**MEDICATION TYPE**	
**1.** Antibiotics<br>☐ Azithromycin<br>☐ Other antibiotic, specify: _______________	**2.** Anti-inflammatory/immunomodulatory<br>☐ Tocilizumab ☐ Systemic corticosteroids<br>☐ Colchicine ☐ Inhaled corticosteroids<br>☐ Interferon ☐ Other anti-inflammatory: ____________
**3.** Anti-viral<br>☐ Chloroquine/hydroxychloroquine<br>☐ Lopinavir-ritonavir<br>☐ Oseltamivir ☐ Remdesivir<br>☐ Other antiviral: ________________________	**4.** Anti-coagulants<br>☐ Direct oral anticoagulant<br>☐ Unfractionated Heparin<br>☐ Low Molecular Weight Heparin<br>☐ Warfarin<br>☐ Other: ________________________
**5.** Other COVID relevant medications:________________________________	**4b.** If DOAC, UFH, LMWH or other:<br>Dose: ☐Prophylactic ☐Therapeutic
Was this medication administered as part of a study? ☐ Yes ☐ No<br>i. Name of study:<br>ii. Arm of study: ☐Drug listed above or ☐UNKNOWN—blinded study	

**Medication Name:** ____	Medication start date (DD/MM/YYYY): _ _ / _ _ / _ _ _ _<br>Medication stop date (DD/MM/YYYY): _ _ / _ _ / _ _ _ _
**MEDICATION TYPE**	
**1.** Antibiotics<br>☐ Azithromycin<br>☐ Other antibiotic, specify: _______________	**2.** Anti-inflammatory/immunomodulatory<br>☐ Tocilizumab ☐ Systemic corticosteroids<br>☐ Colchicine ☐ Inhaled corticosteroids<br>☐ Interferon ☐ Other anti-inflammatory: ____________
**3.** Anti-viral<br>☐ Chloroquine/hydroxychloroquine<br>☐ Lopinavir-ritonavir<br>☐ Oseltamivir ☐ Remdesivir<br>☐ Other antiviral: ________________________	**4.** Anti-coagulants<br>☐ Direct oral anticoagulant<br>☐ Unfractionated Heparin<br>☐ Low Molecular Weight Heparin<br>☐ Warfarin<br>☐ Other: ________________________
**5.** Other COVID relevant medications:________________________________	**4b.** If DOAC, UFH, LMWH or other:<br>Dose: ☐Prophylactic ☐Therapeutic
Was this medication administered as part of a study? ☐ Yes ☐ No<br>i. Name of study:____________________________<br>ii. Arm of study: ☐ Drug listed above or ☐UNKNOWN—blinded study	

Pregnancy Outcomes

1. Date of follow up call (DD/MM/YYYY): _ _ / _ _ / _ _ _ _
2. Birth status: ☐ Live ☐ Stillborn ☐ Miscarriage ☐ Other:________________
3. Date of birth or termination/end of pregnancy (DD/MM/YYYY): _ _ / _ _ / _ _ _ _
4. Where there any serious maternal issues during pregnancy or delivery? ☐ Yes ☐ No
   a. If yes, describe the maternal issues:

5. Where there any fetal/newborn issues? ☐ Yes ☐ No
   a. If yes, describe the fetal/newborn issues:

*follow AE/SAE reporting guidelines in protocol in the event of any issues identified during follow-up call

Re-consent form

1. Date of re-consent: (DD/MM/YYYY): _ _ / _ _ / _ _ _ _
2. Reason for re-consent:
   - ☐ Written consent required (verbal consent initially obtained)
   - ☐ Consent of patient required (legal representative initially obtained)
   - ☐ Participant has reached the age of 18
   - ☐ New version date of consent
   - ☐ Other: ______________________________________
3. Method of consent: ☐ Verbal ☐ Written
4. Protocol Version: ___________________
5. Consent Version: ___________________
6. Person obtaining consent: ___________

Study #: _ _ _ _ - _ _ _

Non-CCP Transfusion Requirements – *complete up to Day 30/discharge*

#	Product	# Units/ Volume	Apheresis/ Non-Apheresis*	Date of collection (plasma only) (dd.mmm.yyyy)	Date of transfusion (dd.mmm.yyyy)
1					
2					
3					
4					
5					
6					
7					
8					
9					
10					
11					
12					
13					
14					
15					
16					
17					
18					
19					
20					
21					
22					
23					
24					
25					
26					
27					
28					
29					
30					

*applies to PLT or FP only

Medical Imaging Post-Randomization (use as many as needed)

Note: only imaging done for COVID-19 reasons needs to be captured (eg. imaging pre-/post- PICC line insertion does not need to be entered).

**Medical Image 1:**	Date (DD/MM/YYYY): _ _ / _ _ / _ _ _ _
**1.** Type<br>☐ CT Chest<br>☐ Chest Xray	**2.** Result<br>☐ Normal ☐ No result documented<br>☐ Patchy Shadowing<br>☐ Infiltrates<br>☐ Other Abnormal
**Medical Image 2:**	Date (DD/MM/YYYY): _ _ / _ _ / _ _ _ _
**1.** Type<br>☐ CT Chest<br>☐ Chest Xray	**2.** Result<br>☐ Normal ☐ No result documented<br>☐ Patchy Shadowing<br>☐ Infiltrates<br>☐ Other Abnormal
**Medical Image 3:**	Date (DD/MM/YYYY): _ _ / _ _ / _ _ _ _
**1.** Type<br>☐ CT Chest<br>☐ Chest Xray	**2.** Result<br>☐ Normal ☐ No result documented<br>☐ Patchy Shadowing<br>☐ Infiltrates<br>☐ Other Abnormal
**Medical Image 4:**	Date (DD/MM/YYYY): _ _ / _ _ / _ _ _ _
**1.** Type<br>☐ CT Chest<br>☐ Chest Xray	**2.** Result<br>☐ Normal ☐ No result documented<br>☐ Patchy Shadowing<br>☐ Infiltrates<br>☐ Other Abnormal
**Medical Image 5:**	Date (DD/MM/YYYY): _ _ / _ _ / _ _ _ _
**1.** Type<br>☐ CT Chest<br>☐ Chest Xray	**2.** Result<br>☐ Normal ☐ No result documented<br>☐ Patchy Shadowing<br>☐ Infiltrates<br>☐ Other Abnormal
**Medical Image 6:**	Date (DD/MM/YYYY): _ _ / _ _ / _ _ _ _
**1.** Type<br>☐ CT Chest<br>☐ Chest Xray	**2.** Result<br>☐ Normal ☐ No result documented<br>☐ Patchy Shadowing<br>☐ Infiltrates<br>☐ Other Abnormal
**Medical Image 7:**	Date (DD/MM/YYYY): _ _ / _ _ / _ _ _ _
**1.** Type<br>☐ CT Chest<br>☐ Chest Xray	**2.** Result<br>☐ Normal ☐ No result documented<br>☐ Patchy Shadowing<br>☐ Infiltrates<br>☐ Other Abnormal
**Medical Image 8:**	Date (DD/MM/YYYY): _ _ / _ _ / _ _ _ _
**1.** Type<br>☐ CT Chest<br>☐ Chest Xray	**2.** Result<br>☐ Normal ☐ No result documented<br>☐ Patchy Shadowing<br>☐ Infiltrates<br>☐ Other Abnormal

Blood Sample Collection for Viral Load/Antibody testing

Additional Sample

1. Was an additional sample collected? ☐ Yes ☐ No
2. Date and time blood sample collected from patient (DD/MM/YYYY HH:MM): _ _ / _ _ / _ _ _ _ _ _ : _ _
3. Date and time research sample processed (DD/MM/YYYY HH:MM): _ _ / _ _ / _ _ _ _ _ _ : _ _
4. Sample ID number (please make sure you have entered this into REDCap within one week): ____________
5. a. Type of sample: ☐ Serum ☐ Plasma

   b. Type of anticoagulant: ☐ EDTA ☐ Citrate ☐ Lithium ☐Heparin ☐ NaF
6. Number of cryovials collected: ☐ 1 ☐ 2
7. Volume per tube:
   - **a.** Tube 1: _______________
   - **b.** Tube 2: _______________

*Use this form if you collect any additional samples (eg. sample at 48 hours was from a tube with lithium or heparin as the anticoagulant and a sample in a preferred anticoagulant became available later)

Additional Sample

1. Was an additional sample collected? ☐ Yes ☐ No
2. Date and time blood sample collected from patient (DD/MM/YYYY HH:MM): _ _ / _ _ / _ _ _ _ _ _ : _ _
3. Date and time research sample processed (DD/MM/YYYY HH:MM): _ _ / _ _ / _ _ _ _ _ _ : _ _
4. Sample ID number (please make sure you have entered this into REDCap within one week): ____________
5. a. Type of sample: ☐ Serum ☐ Plasma

   b. Type of anticoagulant: ☐ EDTA ☐ Citrate ☐ Lithium ☐Heparin ☐ NaF
6. Number of cryovials collected: ☐ 1 ☐ 2
7. Volume per tube:
   - **a.** Tube 1: _______________
   - **b.** Tube 2: _______________

Adverse Event Source Forms – please refer to protocol for AE/SAE recording and data entry requirements, and reporting timelines

NON-CCP-RELATED AE LOG FOR PATIENT RANDOMIZED TO CCP

Adverse Event Term / Start Date	Relationship to CCP transfusion	Severity Grade (CTCAE v4.0)	Expected complication of COVID-19, critical care or existing condition?	Expedited SAE reporting to sponsor (<96h)	Investigator initials/date / Resolution at Day 30
	☐ Possible<br>☐ Unlikely<br>☐ Not related<br>If definitely or probably related, use transfusion-related AE log	☐ 1 } STOP<br>☐ 2<br>☐ 3<br>☐ 4<br>☐ 5	☐ UNEXPECTED<br>Expected complication of:<br>☐ COVID-19<br>☐ Critical care/illness<br>☐ Pre-existing condition **OR**<br>☐ Study outcome	☐ **Yes** (serious* + unexpected)<br>**No:**<br>☐ Not serious*<br>☐ Expected<br>☐ Study outcome	☐ Resolved ☐ Ongoing<br>☐ Resolved with sequelae<br>☐ Death
	☐ Possible<br>☐ Unlikely<br>☐ Not related	☐ 1 } STOP<br>☐ 2<br>☐ 3<br>☐ 4<br>☐ 5	☐ UNEXPECTED<br>Expected complication of:<br>☐ COVID-19<br>☐ Critical care/illness<br>☐ Pre-existing condition **OR**<br>☐ Study outcome	☐ **Yes** (serious* and unexpected)<br>**No:**<br>☐ Not serious*<br>☐ Expected<br>☐ Study outcome	☐ Resolved ☐ Ongoing<br>☐ Resolved with sequelae<br>☐ Death
	☐ Possible<br>☐ Unlikely<br>☐ Not related	☐ 1 } STOP<br>☐ 2<br>☐ 3<br>☐ 4<br>☐ 5	☐ UNEXPECTED<br>Expected complication of:<br>☐ COVID-19<br>☐ Critical care/illness<br>☐ Pre-existing condition **OR**<br>☐ Study outcome	☐ **Yes** (serious* and unexpected)<br>**No:**<br>☐ Not serious*<br>☐ Expected<br>☐ Study outcome	☐ Resolved ☐ Ongoing<br>☐ Resolved with sequelae<br>☐ Death
	☐ Possible<br>☐ Unlikely<br>☐ Not related	☐ 1 } STOP<br>☐ 2<br>☐ 3<br>☐ 4<br>☐ 5	☐ UNEXPECTED<br>Expected complication of:<br>☐ COVID-19<br>☐ Critical care/illness<br>☐ Pre-existing condition **OR**<br>☐ Study outcome	☐ **Yes** (serious* and unexpected)<br>**No:**<br>☐ Not serious*<br>☐ Expected<br>☐ Study outcome	☐ Resolved ☐ Ongoing<br>☐ Resolved with sequelae<br>☐ Death

*Serious = Results in death, is life-threatening, prolongs hospitalization or needs invasive procedure, leads to disability, congenital anomaly or birth defect, or is not immediately life-threatening but may jeopardize the subject or require intervention to prevent any of these events

Adverse Event Tracking Form V3.0 15 May 2020

CCP-RELATED AE LOG

Adverse Event Term	Relationship to CCP transfusion	Severity		Expected complication of plasma transfusion?	Expedited SAE reporting to sponsor (<24h)	Investigator initials/date
Start Date		CTCAE	ISBT			Resolution at Day 30
	☐ Definite<br>☐ Probable<br><br>If less than probably related, use regular AE log	☐ 1<br>☐ 2<br>☐ 3<br>☐ 4<br>☐ 5	☐ 1<br>☐ 2<br>☐ 3<br>☐ 4	☐ UNEXPECTED<br><br>☐ Expected	**Yes** : ☐ TRALI<br>☐ TACO<br>☐ serious* and unexpected<br>**No:** ☐ Expected<br>☐ Not serious*	
						☐ Resolved ☐ Ongoing<br>☐ Resolved with sequelae<br>☐ Death
	☐ Definite<br>☐ Probable	☐ 1<br>☐ 2<br>☐ 3<br>☐ 4<br>☐ 5	☐ 1<br>☐ 2<br>☐ 3<br>☐ 4	☐ UNEXPECTED<br><br>☐ Expected	**Yes** : ☐ TRALI<br>☐ TACO<br>☐ serious* and unexpected<br>**No:** ☐ Expected<br>☐ Not serious*	
						☐ Resolved ☐ Ongoing<br>☐ Resolved with sequelae<br>☐ Death
	☐ Definite<br>☐ Probable	☐ 1<br>☐ 2<br>☐ 3<br>☐ 4<br>☐ 5	☐ 1<br>☐ 2<br>☐ 3<br>☐ 4	☐ UNEXPECTED<br><br>☐ Expected	**Yes** : ☐ TRALI<br>☐ TACO<br>☐ serious* and unexpected<br>**No:** ☐ Expected<br>☐ Not serious*	
						☐ Resolved ☐ Ongoing<br>☐ Resolved with sequelae<br>☐ Death
	☐ Definite<br>☐ Probable	☐ 1<br>☐ 2<br>☐ 3<br>☐ 4<br>☐ 5	☐ 1<br>☐ 2<br>☐ 3<br>☐ 4	☐ UNEXPECTED<br><br>☐ Expected	**Yes** : ☐ TRALI<br>☐ TACO<br>☐ serious* and unexpected<br>**No:** ☐ Expected<br>☐ Not serious*	
						☐ Resolved ☐ Ongoing<br>☐ Resolved with sequelae<br>☐ Death

*Serious = Results in death, is life-threatening, prolongs hospitalization or needs invasive procedure, leads to disability, congenital anomaly or birth defect, or is not immediately life-threatening but may jeopardize the subject or require intervention to prevent any of these events

STANDARD OF CARE AE LOG

Adverse Event Term / Start Date	Severity Grade (CTCAE v4.0)	<u>Expected</u> complication of COVID-19, critical care or existing condition?	Expedited SAE reporting to sponsor (<96h)	Investigator initials/date / Resolution at Day 30
	☐ 1 ☐ 2 } STOP<br>☐ 3<br>☐ 4<br>☐ 5	☐ UNEXPECTED<br>Expected complication of:<br>☐ COVID-19<br>☐ Critical care/illness<br>☐ Pre-existing condition **OR**<br>☐ Study outcome	☐ **Yes** (serious* and unexpected)<br>**No:**<br>☐ Not serious*<br>☐ Expected<br>☐ Study outcome	
				☐ Resolved ☐ Ongoing<br>☐ Resolved with sequelae<br>☐ Death
	☐ 1 ☐ 2 } STOP<br>☐ 3<br>☐ 4<br>☐ 5	☐ UNEXPECTED<br>Expected complication of:<br>☐ COVID-19<br>☐ Critical care/illness<br>☐ Pre-existing condition **OR**<br>☐ Study outcome	☐ **Yes** (serious* and unexpected)<br>**No:**<br>☐ Not serious*<br>☐ Expected<br>☐ Study outcome	
				☐ Resolved ☐ Ongoing<br>☐ Resolved with sequelae<br>☐ Death
	☐ 1 ☐ 2 } STOP<br>☐ 3<br>☐ 4<br>☐ 5	☐ UNEXPECTED<br>Expected complication of:<br>☐ COVID-19<br>☐ Critical care/illness<br>☐ Pre-existing condition **OR**<br>☐ Study outcome	☐ **Yes** (serious* and unexpected)<br>**No:**<br>☐ Not serious*<br>☐ Expected<br>☐ Study outcome	
				☐ Resolved ☐ Ongoing<br>☐ Resolved with sequelae<br>☐ Death
	☐ 1 ☐ 2 } STOP<br>☐ 3<br>☐ 4<br>☐ 5	☐ UNEXPECTED<br>Expected complication of:<br>☐ COVID-19<br>☐ Critical care/illness<br>☐ Pre-existing condition **OR**<br>☐ Study outcome	☐ **Yes** (serious* and unexpected)<br>**No:**<br>☐ Not serious*<br>☐ Expected<br>☐ Study outcome	
				☐ Resolved ☐ Ongoing<br>☐ Resolved with sequelae<br>☐ Death

*Serious = Results in death, is life-threatening, prolongs hospitalization or needs invasive procedure, leads to disability, congenital anomaly or birth defect, or is not immediately life-threatening but may jeopardize the subject or require intervention to prevent any of these events

Study #: _ _ _ _ - _ _ _

Serious Adverse Event Reporting Form – *must also be entered into REDCap*

This form must be completed, entered, and uploaded into the EDC within 24 or 96 hours of becoming aware of any **reportable SAE.** Alternatively, the investigator may e-sign the SAE eCRF in REDCap. (See protocol and operations manual for expedited reporting timelines).

*Note this form may undergo updates outside of CRF updates; check your regulatory binder or OneDrive for most recent version.

Patient ID#:____________________

Type of Report: □Initial □Follow-up #:_____ SAE identifier:_______________________

Medical Term for Event	
**Reason Why Event Serious**	□Fatal<br>□Life-threatening<br>□Requires hospitalization (overnight or longer) or prolongation of existing hospitalization invasive procedure<br>□Results in persistent or significant disability or incapacity<br>□Results in congenital anomaly or birth defect<br>□Other medically important event
Description of the event.<br>*In case pt died, has this event contributed to the patient's death:*<br>□ *Yes* □ *No*	
Event Onset<br>(dd.mmm.yyyy)- (hh:mm)	
Outcome	□ Recovered, resolved<br>□ Not recovered/resolved (by Day 30) □ Recovered/resolved w/sequelae<br>□ Fatal □ Unknown
Event Stop<br>(dd.mmm.yyyy) – (hh:mm)	
Severity	□Grade 1 □ Grade 2 □ Grade 3 □Grade 4 □Grade 5
**Was patient randomized to CCP?** □Yes □No	
**Relation to CCP**	
Causality	□ Unrelated □ Unlikely □ Possible □ Probable □ Definite
Classification	□ Expected □ Unexpected □ NA
**Action Taken**	
(Check all that apply)	□ None □ Medication □Test performed<br>□ Procedure □ Other Specify: ____
Details: Provide details of medications (name, dose, and duration), tests, procedures or other actions.	

Reporter Name & Role: _____________________________________

PI/Co-I Signature: ____ Date:_________________________________

SAE Reporting Form 19 Aug 2020, Version 4.0 – English

Other Reportable Events

In the event of a TACO/TRALI, overdose, or newly identified pregnancy, complete the Other Reportable Events eCRF (there is no paper form).
The completed form can be printed from the EDC.
