# Supplementary material for: Convalescent plasma for adults with acute COVID-19 respiratory illness (CONCOR-1): study protocol for an international, multicentre, randomized, open-label trial
Source: Trials. 2021 May 4;22:323. doi: 10.1186/s13063-021-05235-3 (PMC8094980; doi:10.1186/s13063-021-05235-3)
Supplement: Supplementary file 2 — Additional file 2. Statistical Analysis Plan Version 2.0 19 Mar 2021. [file 13063_2021_5235_MOESM2_ESM.pdf]

Statistical Analysis Plan for

A Randomized Open-Label Trial of CONvalescent Plasma for Hospitalized Adults With Acute COVID-19 Respiratory Illness (CONCOR-1)

**Co-Lead Principal Investigators:**	Dr Donald Arnold<br>Dr Philippe Bégin<br>Dr Jeannie Callum
**Investigational product:**	COVID-19 Convalescent Plasma
**Protocol #:**	GRAAL-2020-01
**SAP for Protocol Version and Date:**	Version 8.0 15 Jan 2021

The study will be conducted according to the protocol and in compliance with Good Clinical Practice (GCP), with the Declaration of Helsinki and with other applicable regulatory requirements.

Contents

1. Study Design and Objectives 1
1.1. Summary of Design 1
1.2. Primary Study Objectives 1
1.3. Sample Size Justification 1
2. Responses 1
2.1. Primary Outcome 1
2.2. Secondary Outcomes 1
2.2.1. Time to intubation or death 1
2.2.2. Ventilator-free days 1
2.2.3. In-hospital death by Day 90 2
2.2.4. Time to in-hospital death 2
2.2.5. Death by Day 30 2
2.2.6. Length of stay in intensive care unit 2
2.2.7. Length of stay in hospital 2
2.2.8. Need for Extra-Corporeal Membrane Oxygenation (ECMO) 2
2.2.9. Need for renal replacement therapy 2
2.2.10. Myocarditis 2
2.2.11. Patient-reported outcomes (PROs) 3
2.2.12. Incremental cost per quality-adjusted life year (QALY) 3
2.3. Adverse Events 3
2.3.1. Adverse event reporting 3
2.3.2. Adverse events to Convalescent Plasma Transfusion 3
3. Analysis Sets 3
3.1. Intention to Treat Sample 3
3.2. Per Protocol Sample 4
3.3. Safety Sample 4
4. Statistical Analysis 4
4.1. Handling of Incomplete Data 4
4.2. Analysis of Primary Outcome 5
4.3. Analyses of Secondary Outcomes 5
4.3.1. Time to intubation or death 5

4.3.2. Ventilator-free days 5
4.3.3. In-hospital death by Day 90 5
4.3.4. Time to in-hospital death 6
4.3.5. Death by Day 30 6
4.3.6. Length of stay in intensive care unit 6
4.3.7. Length of stay in hospital 6
4.3.8. Need for Extra-Corporeal Membrane Oxygenation (ECMO) 7
4.3.9. Need for renal replacement therapy 7
4.3.10. Myocarditis 7
4.3.11. Patient-reported outcomes (PROs) 7
4.3.12. Incremental cost per quality-adjusted life year (QALY) 7
4.3.13. CPP transfusion-associated adverse events at Day 30 7
4.3.12. Grade 3, 4, and 5 adverse events at Day 30 8
4.3.13. Grade 3, 4 and 5 serious adverse events at Day 30 8
4.4. Sub-Group Analyses 8
4.5. Safety Reports for the IDSMC 9
4.6. Exploratory Analysis of the Role of anti-SARS-CoV-2 antibodies 9
4.6.1. Neutralizing antibodies 9
4.6.2. Viral load 10
4.7. Schedule for Primary Interim Analysis 10
4.8. The Lan-DeMets Error Spending Function 11
4.9. Interim Calculation of Conditional Power for Futility 11
4.10. Sample Size Re-Estimation 12
4.11. Conventions Regarding Blinding 12
5. References 13

1. Study Design and Objectives

1.1.Summary of Design

This is a multi-centre open-label randomized controlled trial in which recruited patients will be randomized in a 2:1 ratio to receive either 500 mL of COVID-19 convalescent plasma (CCP) comprised of a 500 mL unit from one donor or two 250 mL units from one or two donors collected by apheresis, or standard of care. The plan is to randomize 1200 individuals to obtain an expected 800 patients in the CCP arm and 400 patients in the arm receiving standard of care.

1.2.Primary Study Objectives

The primary objective is to determine the effect of COVID-19 convalescent plasma (CCP) on the risk of intubation or death at Day 30 in adult patients hospitalized for COVID-19 respiratory illness.

1.3.Sample Size Justification

Assuming a 30% risk of the composite outcome of intubation or death by Day 30 among hospitalized patients under standard of care, a sample size of 1200 individuals (800 in the CCP arm, and 400 in the standard of care arm) will provide 80% power ($\beta = 0.20$) to detect a relative risk reduction of 25% with CCP therapy using a 2-tailed test at level $\alpha = 0.05$. We assume that no patient will be lost to follow-up.

2. Responses

2.1.Primary Outcome

The primary outcome is intubation or death at Day 30 (the date of randomization being Day 1).

2.2.Secondary Outcomes

2.2.1.Time to intubation or death

The number of days from randomization to the first event of intubation or in-hospital death will be recorded. The censoring time for individuals not receiving intubation or dying will be the minimum of i. the number of days from randomization to discharge, and ii. Day 30. The 30-day limit is specified to align with the time horizon of the primary outcome.

2.2.2.Ventilator-free days

The number of ventilator-free days between randomization and Day 30 will be computed as the number of days during this 30-day period of time during which they were alive and not requiring ventilation. This total number of days could be made up days prior to ventilation and the number of days after discontinuation of ventilation if ventilation is required. Patients will be contacted at Day 30 by telephone to determine whether there were any ICU admissions since discharge from the hospital they were in at the time of recruitment. Patients discharged from hospital will be assumed to be off-ventilator post-discharge unless otherwise indicated.

2.2.3.In-hospital death by Day 90

This response will be a binary indicator of whether a patient died in-hospital within 90 days of randomization.

2.2.4.Time to in-hospital death

The number of days from randomization to in-hospital death will be recorded. The censoring time for individuals not dying in hospital will be the minimum of i. the number of days from randomization to discharge, and ii. to Day 90. The 90-day limit is specified to be large enough that it is highly unlikely to be the factor leading to censoring, but is small enough that it will mitigate the need to follow individuals who are hospitalized for an unusually long time until they are discharged.

2.2.5.Death by Day 30

This response is a simple binary indicator indicating whether the patient has died between randomization and Day 30.

2.2.6.Length of stay in intensive care unit

The number of days spent in the intensive care unit (ICU) over the 30-day period following randomization will be recorded. This total number of ICU days could be made up days from multiple admissions within the 30-day period from randomization if they are discharged and re-admitted a second time within this 30-day period. Patients will be contacted at Day 30 by telephone to determine whether there were any hospital admissions since discharge from the hospital they were in at the time of recruitment. Patients discharged from hospital will be assumed not to be in an ICU elsewhere, unless otherwise indicated.

2.2.7.Length of stay in hospital

The number of days from randomization to the first event of death or discharge will be recorded as the length of stay in hospital. This time will be right-censored at 90 days to avoid the need to follow individuals who are hospitalized for a particularly long time until discharge; it is expected that most hospitalizations will not last beyond 90 days.

2.2.8.Need for Extra-Corporeal Membrane Oxygenation (ECMO)

This response will be a binary indicator indicating whether the patient required extra-corporeal membrane oxygenation during the 30-day period following randomization.

2.2.9.Need for renal replacement therapy

This response will be the binary variable indicating that the patient required renal replacement therapy during the 30-day period following randomization.

2.2.10. Myocarditis

This binary response will indicate whether the patient developed myocarditis (as defined by the medical team) between randomization and Day 30.

2.2.11.Patient-reported outcomes (PROs)

PRO will be measured using a widely used generic instrument called EQ-5D-5L. It measures health-related quality of life in five dimensions, namely, mobility, self-care, usual activities, pain/discomfort, and anxiety/depression. Patients can report five level impairment, reflecting no, slight, moderate, severe, and extreme problems in each dimension. The EQ-5D-5L will be administered at baseline and Day 30.

2.2.12.Incremental cost per quality-adjusted life year (QALY)

A cost effectiveness analysis alongside the trial will be conducted. The summary measure for the economic evaluation is incremental cost per QALY comparing CCP with standard care over the 30-day trial period.

2.3.Adverse Events

2.3.1.Adverse event reporting

The severity of each AE will be assessed by the Investigator and graded based on the Common Terminology Criteria for Adverse Events (CTCAE) version 4.0. Adverse events graded as 1 or 2 require no further documentation, unless they are related or probably related to CCP. Adverse events graded as 3, 4 and 5 must be reported on the eCRF within 7 day and are categorized as expected or unexpected. Serious adverse events (SAEs) deemed to be unexpected and related to or probably related to the investigational products must be reported to the sponsor within 24 hours. All other SAEs must be reported to the sponsor within 96 hours (Protocol section 9).

2.3.2.Adverse events to Convalescent Plasma Transfusion

Adverse events to convalescent plasma will be captured and graded using two approaches:

1. CCP-related AEs will be graded using the CTCAE criteria as specified in 2.3.1
2. CCP-related AEs will also be categorized using the ISBT criteria for acute transfusion reactions including name of the reaction, and severity (Grade 1-4)

3. Analysis Sets

Given the simple nature of the outcomes it is anticipated that outcomes will be possible to collect for most individuals.

3.1. Intention to Treat Sample

The intention to treat (ITT) sample will be comprised of all patients who were randomized irrespective of whether they received the randomized intervention. That is, individuals randomized to the CCP arm who did not receive CCP would have their outcomes attributed to the CCP arm. Given the simple nature of the primary outcome we anticipate a very low rate of missing primary outcome data.

The primary analysis will be based on individuals with available outcome data and this is expected to be all patients given the simple nature of the primary outcome. For some secondary outcomes if an outcome is unknown for some individuals, analyses will be first

carried out for those with complete data (i.e. in a complete case analysis) based on a modified intention to treat sample (i.e. the intention to treat sample excluding those individuals with unknown outcome). Multiple imputation will be carried out for a sensitivity analysis in which outcome data will be generated for those where it is missing in order to approximate a true intention to treat analysis. For details of the multiple imputation procedure see Section 4.1.

3.2.Per Protocol Sample

The per protocol sample will be comprised of eligible individuals who are randomized and are treated according to the protocol. In this trial, patients will be excluded from the per protocol analysis if any of the following protocol violations occur:
- Received less than two units of CCP*
- Transfusion of second unit of CCP completed > 24 hours after randomization*
- Patient randomized to SOC but receives CCP
- Patient was randomized despite being ineligible for the trial

*special consideration will be given for instances where 1 x 500 mL unit was given

3.3.Safety Sample

The safety sample will be comprised of all randomized individuals and so will coincide with the intention to treat sample.

4. Statistical Analysis

In what follows, the analysis plans are described for the sample representing the intention to treat population. Similar analyses will be carried out for the modified intention to treat and per protocol samples.

4.1.Handling of Incomplete Data

Efforts to minimize loss-to-follow up will be considerable and we are expected to have complete data on individuals for the primary and secondary outcomes. Given the simple nature of the primary outcome it is not expected that this response will be missing. If an individual withdraws from the study early, their time to event outcomes will be censored at the time of withdrawal. For binary outcomes where data are missing, a first analysis will be based on a complete case analysis using individuals who provide information on the outcomes; this is not strictly an intention to treat analysis but rather is based on a modified intention to treat sample. There is a chance that the complete case analysis will yield biased estimates of treatment effect (Kenward and Carpenter, 2007); therefore, while there is controversy about the appropriateness of imputing outcome data in clinical trials, multiple imputation (Little and Rubin, 2002) will be used as a sensitivity analysis to enable a genuine intention to treat sample. This will involve imputing missing data via imputation models fitted to the data from individuals providing complete information. Covariates to be included in the imputation model include age, sex, key co-morbidities (e.g. diabetes, cardiovascular disease, kidney disease), treatment and the time of withdrawal from the study**.** Standard multiple imputation will be adopted using *m* =

50 imputed datasets with the results combined across samples and variance estimates computed based on Rubin's rules (Rubin, 1996).

4.2.Analysis of Primary Outcome

If we let p_e and p_s denote the probability of the composite outcome (intubation or death at Day 30) for the experimental (CCP) and standard of care arms respectively, then the null and alternative hypotheses are H_0: $p_e = p_s$ and H_1: $p_e \neq p_s$, respectively. The probability of intubation or death between randomization and Day 30 will be estimated for each treatment group by the proportion of individuals randomized to each group who required ventilation or died by Day 30. The primary analysis will be based on a 2-sided Wald test of the null hypothesis that probability of intubation or death is the same among individuals receiving CCP or standard of care. The percent relative risk reduction, defined by $100(1 - p_e/p_s)$, will be estimated along with a 95% confidence interval.

4.3.Analyses of Secondary Outcomes

4.3.1.Time to intubation or death

The time to intubation for mechanical ventilation will be analyzed using a cause-specific Cox regression model with censoring occurring at the time of discharge (Kalbfleisch and Prentice, 2011). That is, individuals who are intubation-free and alive at the time of discharge will have their follow-up censored at the time of hospital discharge. The regression coefficient and associated standard error from the cause-specific Cox regression model for intubation or in-hospital death will be used to compute an estimate of the cause-specific hazard ratio along with a 95% confidence interval and a p-value.

To accompany this analysis, estimates of the cumulative incidence functions for the composite event of intubation or death will plotted for each arm

4.3.2.Ventilator-free days

The total number of ventilator-free days will be computed for each individual between randomization and Day 30. For each treatment group, the mean ventilator-free days will be computed by the sample average, and an associated robust bootstrap standard error will be obtained by resampling with replacement from the respective arm B=200 times. The difference in the sample means will be computed, along with a 95% confidence interval obtained based on the robust standard errors. A Wald test of the null hypothesis that there is no difference in the mean ventilator-free days between groups will be carried out using a robust variance obtained from the individual bootstrap standard errors.

4.3.3.In-hospital death by Day 90

The probability of in-hospital death within 90 days of randomization will be estimated for each treatment group by the proportion of individuals randomized to each group who died in hospital within the first 90 days of study. The primary analysis will be based on a 2-sided Wald test of the null hypothesis that probability of death by 90 days is the same among individuals

receiving CCP or standard care. The percent relative risk reduction will be estimated along with a 95% confidence interval and a p-value of the null hypothesis will be computed.

4.3.4.Time to in-hospital death

The time to in-hospital death will be analyzed using a cause-specific Cox regression model to deal with the competing risks of discharge from the hospital in which the individual was randomized; for subjects hospitalized for more than 90 days their time of in-hospital death will be censored at 90 days. That is, individuals who did not die in the hospital they were admitted to at the time of randomization will have their follow-up censored at the time of discharge from this hospital. The regression coefficient and associated standard error from the cause-specific Cox regression model for death in the original hospital will be used to compute an estimate of the cause-specific hazard ratio along with a 95% confidence interval and a p-value. To accompany this analysis estimates of the cumulative incidence functions will be plotted for in-hospital death and discharge for each treatment group.

4.3.5.Death by Day 30

The probability of death between randomization and Day 30 will be estimated for each treatment group by the proportion of individuals randomized who died between randomization and Day 30. The test will be based on a 2-sided Wald test of the null hypothesis that the probability of death by Day 30 is the same among individuals receiving CCP or standard of care. The percent relative risk reduction will be estimated along with a 95% confidence interval and a p-value of the null hypothesis will be computed.

4.3.6.Length of stay in intensive care unit

The total number of days in the intensive care unit (ICU) will be computed for each individual over the 30-day period from the time of randomization. For each treatment group, the mean number of ICU days will be computed by the sample average, and an associated robust bootstrap standard error will be obtained by resampling with replacement from the respective arm B=200 times. The difference in the sample means will be computed, along with a 95% confidence interval obtained based on the robust standard errors. A Wald test of the null hypothesis that there is no difference in the mean number of ICU days between groups will be carried out using a robust variance obtained from the individual bootstrap standard errors.

4.3.7.Length of stay in hospital

The length of stay in hospital over 90 days will be computed as the time from randomization to death or discharge disregarding the reason for the end of the hospitalization. A Cox regression model will then be fitted with this time as the response with an administrative censoring time set a 90 days from the day of randomization. The regression coefficient and associated standard error from the Cox regression model will be used to compute an estimate of the hazard ratio, along with a 95% confidence interval and a p-value. Kaplan-Meier estimates will be computed and plotted for the distribution of the time to the end of hospitalization for each arm of the study.

4.3.8.Need for Extra-Corporeal Membrane Oxygenation (ECMO)
The probability of requiring ECMO between randomization and Day 30 will be estimated for each treatment group by the proportion of individuals randomized to each group requiring ECMO between randomization and Day 30. The primary analysis will be based on a 2-sided Wald test of the null hypothesis that the probability of requiring ECMO is the same among individuals receiving CCP or standard of care. The percent relative risk reduction will be estimated along with a 95% confidence interval.

4.3.9.Need for renal replacement therapy
The probability of requiring renal replacement therapy between randomization and Day 30 will be estimated for each treatment group by the proportion of individuals randomized to each group requiring renal replacement therapy between randomization and Day 30. The primary analysis will be based on a 2-sided Wald test of the null hypothesis that the probability of requiring renal replacement therapy is the same among individuals receiving CCP or standard of care. The percent relative risk reduction will be estimated along with a 95% confidence interval.

4.3.10.Myocarditis
The probability of developing myocarditis over the first 30 days will be estimated in a similar fashion to the way that the need for ECMO and renal replacement therapy are estimated and tests of effects will likewise be reported in a similar fashion.

4.3.11.Patient-reported outcomes (PROs)
The original responses from each patient will be converted into a single utility index score anchored at 0 for dead and 1 for full health using the scoring algorithm based on Canadian society preferences. Utility changes from baseline to Day 30 were estimated by analysis of covariance adjusting for baseline utilities and compared between the two arms.

4.3.12.Incremental cost per quality-adjusted life year (QALY)
In addition to the cost of CCP, we will estimate the cost associated with the stay in ICU, hospitalization, use of ventilator and ECMO, diagnostics and procedures, and medications. The difference in cost between CCP and standard care will be divided by the difference in QALY calculated using the EQ-5D-5L to derive the incremental cost per QALY for CCP. 95% confidence interval for the incremental ratio will be assessed using a non-parametric bootstrapping with replacement approach with B=1000 times.

4.3.13. CPP transfusion-associated adverse events at Day 30
Adverse events judged to be definitely or probably related to CPP will be analyzed two ways: 1) using the Common Terminology Criteria for Adverse events (CTCAE; Grading 1-5)); and using the ISBT definitions for acute transfusions reactions (Grades 1-4). (Protocol section 4.3.13),

1. CTCAE Grading: The proportion of individuals experiencing CPP transfusion-associated adverse events (CTCAE Grades 1-5) will be reported overall by type, and severity. The total number of CPP transfusion-associated adverse

events will also be tabulated overall by type, and severity which will accommodate the possibilities of multiple adverse events per individual.

2. ISBT Grading: The proportion of individuals experiencing CPP transfusion-associated adverse events (ISBT Grades 1-4) will be reported overall by type, and severity. The total number of CPP transfusion-associated adverse events will also be tabulated overall by type, and severity which will accommodate the possibilities of multiple adverse events per individual.

4.3.12. Grade 3, 4, and 5 adverse events at Day 30

See protocol section 9 for additional details regarding AE definitions.

The proportion of individuals experiencing Grade 3, 4 or 5 (as per CTCAE criteria) adverse events will be analyzed overall and by treatment arm by type, severity grade, and expected/unexpectedness. The total number of Grade 3, 4 or 5 adverse events will also be tabulated overall and by treatment arm by type, severity grade and expected/unexpected which will accommodate the possibilities of multiple events per individual.

4.3.13. Grade 3, 4 and 5 serious adverse events at Day 30

Serious adverse events (unexpected and meeting criteria for expedited reporting to the sponsor, Hamilton Health Sciences) will be analyzed as summarized below. TRALI and TACO adverse events, while not technically considered SAEs, require expedited reporting to the sponsor; hence they will be also be included.

The number and proportion of individuals experiencing a serious adverse event will be reported by event type, overall and by treatment arm. A second analysis will be by event which will accommodate the possibilities of multiple events per individual.

In the interim and final analyses, the relative risk reduction for death (CCP versus standard of care) will be computed along with a 95% confidence interval. The relative risk reduction (CCP versus standard of care) will also be computed for SAEs (regardless of outcome) along with a 95% confidence interval.

The cumulative incidence of serious adverse event will also be plotted by treatment group.

4.4.Sub-Group Analyses

Subgroup analyses will be carried out to assess whether the effect of treatment varies across subgroups defined according to the following criteria:

- Patients enrolled vs. not enrolled/treated in other therapeutic clinical trials

- >= 60 years versus less than 60 years of age
- Sex
- Ethnicity
  - ➢ White/Black/Hispanic/Asian/Other

- Presence of medical co-morbidities
    - ➢ diabetes (yes/no)
    - ➢ cardiac (yes/no)
    - ➢ respiratory (yes/no)
- Smoking History
    - ➢ Ever smoker/current smoker/never
    - ➢ 15 pack year history of smoking versus not
- Obesity (BMI >= 30 vs <30)
- Onset of symptoms > 12 days before randomization (including non-respiratory symptoms) versus not
- Timing of administration of CCP from diagnosis of COVID-19 (<=72 hours vs >72 hours)
- Severity of illness of donors (hospitalized vs not)
- ABO blood type of recipients (A vs other, B vs other, O vs other, AB vs other)
- Detectable vs non-detectable viral plasma viral load at baseline
- Anti-SARS-CoV-2 seropositivity at baseline
- Oxygen status at baseline (≤ 2L vs > 2L)

These subgroup analyses will be carried out by estimating the treatment effect within sub-groups and providing 95% confidence intervals and p-values.

In addition the effect of plasma from one donor will be compared to the effect of plasma from two donors.

Any additional exploratory sub-group analyses carried out will be based on regression models involving treatment, the subgroup variable and the respective interaction. The findings from such post-hoc analyses will be reported as hypothesis generating if the test of the null hypothesis that the interaction term is zero gives a statistically significant finding of heterogeneity of the effect.

4.5.Safety Reports for the IDSMC

Monthly reports on logistical issues, donor recruitment, and patient accrual overall and by study site will be provided to the IDSMC. Information on antibody test results will also be provided as available. These reports will also give tabulations of i. adverse events, ii. serious adverse events, and iii. CPP transfusion-related adverse events. If the IDSMC requests more or less frequent reports on this data, they will be provided. The reports will be created by an independent statistician who is unblinded to the treatment assignment.

4.6.Exploratory Analysis of the Role of anti-SARS-CoV-2 antibodies

4.6.1.Neutralizing antibodies

Donor samples will be tested for the presence of total anti-SARS-CoV-2 antibodies and neutralizing antibodies for all CCP units transfused. Moreover, there will be samples taken from

study participants to assess antibody titre at the time of randomization and 48 hours later. The results of the donor antibody testing will be used to study the effect of neutralizing antibodies on clinical outcomes. The results of antibody testing of participants will enable investigation of the effect of CCP transfusion on participant antibody levels.

The total and neutralizing antibody titres for the donor samples will be recorded. In order to assess the effect-modifying role of these titres on the outcome, we will consider regression models for the primary outcome with covariates based on treatment arm, titres and interactions terms. For the primary binary outcome, a logistic regression model will be fitted involving the treatment indicator (equaling 1 for those in the CCP arm), an “interaction term” defined as the product of the treatment indicator and the log transformed and centered total antibody titre for the plasma transfused, and a second “interaction term” defined as the product of the treatment indicator and the log transformed and centered neutralizing antibody titre for the plasma transfused. The main effect of treatment will then correspond to the effect of CCP for a unit with the geometric mean total antibody titre and the geometric mean neutralizing antibody titre. The coefficient of the first “interaction term” will reflect how this CCP effect is moderated according to the total antibody titre for the plasma sample transfused, while the second “interaction term” will reflect how the CCP effect is moderated by the neutralizing antibody titre of the plasma sample transfused; the phrase “interaction term” is in quotation marks because the main effects for these titre based variables are not included in the linear predictor.

Similar analyses to those for the total and neutralizing antibodies will be carried out incorporating information of the patients own total and neutralizing antibody profiles prior to the plasma transfusion to explore how the benefit of CCP may vary depending on the antibody profile of the recipient. These analyses will be carried out at the interim analysis for the IDSMC and at the time of study completion.

For a fuller description of the handling of the laboratory data and analyses please see *Statistical Analysis Plan for A Randomized Open-Label Trial of CONvalescent Plasma for Hospitalized Adults with Acute COVID-19 Respiratory Illness: Supplement Regarding Antibody Analyses*

4.6.2.Viral load

A sample from study participants will be collected at the time of randomization (up to 48 hours before randomization) and 48 hours later (up to 5 days later) to measure RNA levels of SARS-CoV-2. This information will enable an analysis of the effect of CCP on viral load.

4.7.Schedule for Primary Interim Analysis

A single interim analysis is planned when information on intubation or death at Day 30 is available for 50% of the target sample (600 patients: 400 in the CCP arm and 200 in the arm receiving standard care). An O'Brien-Fleming stopping rule will be employed with a threshold for formal statistical significance at the interim analysis of 0.0054 and the final significance level of 0.0492. The Independent Data Safety Monitoring Committee (IDSMC) may recommend early

termination or modification of the protocol only when there is clear and substantial evidence of a treatment difference. More details about the interim analyses are described below as well as a separate guidance document for the IDSMC.

4.8.The Lan-DeMets Error Spending Function

In case any additional interim analyses are requested by the IDSMC, the flexible Lan-DeMets (Lan and DeMets, 1983) type I error spending function analog of the O'Brien-Fleming boundaries will be used to monitor the primary endpoint as a guide for the IDSMC for an overall two-sided type I error rate of 0.05 based on a two-sided test. This is defined as follows. If we let n denote the sample size according to the calculation for the planned study and n_o denote the number of individuals at an interim analysis, then let $t = n_o/n$. This “O'Brien-Fleming" error spending function has the form

$$\alpha(t) = 2 - 2\Phi(Z_{\alpha/2}/\sqrt{t})$$

where $Z_{\alpha/2} = 1.96$ reflects the critical value corresponding to the analysis that would be done for an analogous trial with a fixed sample size and a two-sided test (Lans and DeMets, 1983). For the interim analysis planned when 50% of the sample will be available $t = 1/2$ and we obtain

$$\alpha(1/2) = 2(1 - \Phi(Z_{\alpha/2}/\sqrt{0.5})) = 0.005573$$

as the threshold for significance and stopping at the interim analysis. This constitutes a conservative stopping rule which we will adopt to preserve much of the type I error for the analysis at the conclusion of the study, to minimize the bias in estimates of the treatment effect from sequential trials, and to gear the trial towards one that will be large enough to answer the several important secondary questions we aim to address including the effect of neutralizing antibodies.

4.9.Interim Calculation of Conditional Power for Futility

Conditional power will be presented as an additional guide to the IDSMC for the primary outcome based on a test for the difference in two binomial proportions (Jennison and Turnbull, 2000). Conditional power allows computation of the probability of obtaining a statistically significant result by the end of the trial given the data accumulated thus far, incorporating a hypothesized treatment effect (e.g., the treatment effect assumed for sample size determination) thereafter. If the conditional power is less than 20% under the original trial assumptions, consideration could be given to stop the trial. An unblinded independent statistician will prepare the reports for IDSMC to review when making their recommendations.

The interim analysis report will be created by an independent statistician who will have access to the randomization code.

4.10.Sample Size Re-Estimation

There is uncertainty about the intubation and mortality rate that will be observed in this study due to possibly evolving profile of COVID-19 patients admitted to hospital over the course of the pandemic. To address this, a blinded sample size re-estimation will take place when the primary outcome is available for 50% of the target sample, which coincides with the time of the interim analysis report. Sample size will be adjusted if the estimated event rate is different from that originally specified to the extent that the study is not expected to achieve the desired nominal power for the test of the primary outcome. Friede and Kieser (2004) give the following formula for total sample size re-estimation: We let $n = n_e + n_s$ denote the total sample size where n_e and n_s denote the number of individuals in the experimental (CCP) and standard of care arms respectively, and let q denote the randomization ratio where $n_s = \theta n_e = 0.5 n_e$. We let d be the absolute risk difference we wish to detect (which was specified in the original sample size calculation). Then if we observe a pooled composite event rate *p*, then

$$n = n_e + n_s = \left(2 + \theta + \frac{1}{\theta}\right)\frac{\left(Z_{\alpha/2} + Z_\beta\right)^2}{\delta^2} p(1-p),$$

gives the revised total sample size.

4.11.Conventions Regarding Blinding

It is generally recognized that blinding of the investigators to the arm-specific data is desirable during the course of a clinical trial. The monthly safety reports being prepared for the IDSMC will display data on trial conduct aggregated across centers and specific to each center, and will give unblinded safety data in order to equip them to assess safety.

At the point of the interim analysis being conducted when 50% of the target sample size can provide primary outcome data, again the data will be presented with the randomized treatment arm clearly specified in order to equip the IDSMC with the information needed to interpret the results of the interim analyses.

A blinded version of the monthly Progress and Safety Report will be created for the Executive Committee in which the data will only be presented in such a way that the treatment arm cannot be identified; this will be achieved by using generic labels A and B for the two arms, not reporting the sample sizes for these groups, and reporting simply the percentages of individuals experiencing particular events, averages, and standard deviations rather than standard errors for continuous variables. For some features data will only be presented in aggregate form.

The sample size re-estimation will be conducted by a blinded investigator (R. Cook) based on data aggregated across the two arms as described in Sections 4.9. The rationale is that this calculation is the responsibility of the investigators and the investigators ideally remain blinded to the interim outcomes.

As the independent statistician preparing the interim analysis report is unblinded and the IDSMC members are unblinded, they will be equipped to assess the suitability of the revised target sample size.

5. References

Friede T and Kieser M (2004). Sample size recalculation for binary data in internal pilot study designs. *Pharmaceutical Statistics*, 3: 269-279.

Herdman M, Gudex C, Lloyd A, et al (2011). Development and preliminary testing of the new five-level version of EQ-5D (EQ-5D-5L). *Quality of Life Research,* 20(10):1727-1736.

Jennison C and Turnbull BW (2000). *Group Sequential Methods with Applications to Clinical Trials*. Chapman and Hall, Boca Raton.

Kalbfleisch JD and Prentice RL (2011). *The Statistical Analysis of Failure Time Data (Vol. 360)*. John Wiley and Sons.

Kenward MG and Carpenter J (2007). Multiple imputation: current perspectives. *Statistical Methods in Medical Research*, 16(3), 199-218.

Lan K and DeMets D (1983). Discrete sequential boundaries for clinical trials. *Biometrika*, 70: 659-663.

Little RJA and Rubin DB (2002). *Statistical Analysis with Missing Data, 2nd edition*. Wiley

O'Brien P and Fleming T (1979). *Multiple Testing Procedure for Clinical Trials*. Biometrics, 35: 549-556.

Rubin DB. (1996). Multiple imputation after 18+ years. *Journal of the American Statistical Association*, 91: 473-490.

Statistical Analysis Plan for
A Randomized Open-Label Trial of CONvalescent Plasma for Hospitalized Adults With Acute COVID-19 Respiratory Illness: Supplement regarding Antibody Analyses
(CONCOR-1)

**Co-Lead Principal Investigators:**	Dr. Donald Arnold<br>Dr. Philippe Bégin<br>Dr. Jeannie Callum
**Investigational product:**	COVID-19 Convalescent Plasma
**Protocol #:**	GRAAL-2020-01
**Auxiliary SAP for Protocol :**	Version v8.0 15 Jan 2021

The study will be conducted according to the protocol and in compliance with Good Clinical Practice (GCP), with the Declaration of Helsinki and with other applicable regulatory requirements.

This document contains confidential information.
Do not copy or distribute without written permission from the Sponsor.

CONFIDENTIAL

Contents

1 Laboratory Markers To Be Examined as Possible Effect Modifiers 1

1.1 Anti-RBD total Ig as Measured by ELISA 1

1.2 Anti-SPIKE IgG as Measured by Flow Cytometry 1

1.3 Plaque Reduction Neutralization Titer 50% (PRNT50) 1

1.4 Antibody-Dependent Cell Cytotoxicity (ADCC) 1

1.5 Combining Antibody Data from Two Plasma Units 2

1.5.1 Dealing with Replicate Measurements on a Given Plasma Unit 2

1.5.2 Combination of Test Results from Different Plasma Units 2

1.6 Log Transformation Before Analysis 2

1.7 Handling Missing Data 2

1.7.1 Missing Unit Volumes 2

1.7.2 Missing Markers for Particle Providers 2

1.7.3 Results missing for markers when measurements are expected 3

2 Statistical Analyses Involving Laboratory Data 3

2.1 Regression Analyses Investigating Role of Markers in the CCP Arm 3

2.2 Regression Analyses Exploring the Effect-Modifying Role of Each Marker 4

2.3 Multivariate Regression Analyses of Effect-Modifying Roles of Markers 4

2.4 Incorporating the Patient Marker Values 5

2.4.1 Exploring the Effect of Patient Marker Values 5

2.4.2 Incorporating the Patient Marker Values - Effect Modifying Roles 5

3 References 5

1 Laboratory Markers To Be Examined as Possible Effect Modifiers

Here we give a clear description of the markers under consideration that are measured in the convalescent plasma transfused in the CCP arm of CONCOR-1.

1.1 Anti-RBD total Ig as Measured by ELISA

This marker is reported as the optic density (O.D.), which is a measure directly proportional to the amount of antibody. This is recorded into the REDCap database directly.

1.2 Anti-SPIKE IgG as Measured by Flow Cytometry

This marker is reported as the mean fluorescence intensity (MFI), which is directly proportional to the amount of antibody. This is recorded in the REDCap database directly.

1.3 Plaque Reduction Neutralization Titer 50% (PRNT50)

This marker is reported as the highest titer at which the plasma still inhibits at least 50% of viral entry in cells in a plaque reduction assay. Titers are determined by doubling the dilution at each stage, leading to discrete values ranging from 1:20 to $\geq$1:640 (or $\geq$ 1:1280 for some plasma samples tested at the beginning of the pandemic). Before uploading into REDCap:

- Left-censored values will be dealt with via mid-point imputation so if 50% of the viral entry in cells is not inhibited at the first dilution, then the value is taken as 1 : 10 where 10 is halfway between 0 and 20.
- Right-censored values where changed as follows:
  - If it was determined that 50% of the cells where inhibited at at the highest dilution of 640 (i.e. we know it is $\geq$ 1:640), the value was taken to be 1:640
  - If it was determined that 50% of the cells where inhibited at at the highest dilution of 1280 (i.e. we know it is $\geq$ 1:1280), the value was taken to be 1:1280
- Ultimately the dilution rate was inverted to express titers in terms of the number of dilutions so a titer of 1:40 was expressed as 40 dilutions

1.4 Antibody-Dependent Cell Cytotoxicity (ADCC)

This marker is reported as the proportion of the target cells that were killed by NK cells in presence of the antibody. Before uploading in REDCap the ADCC proportion X was re-expressed as the ADCC ratio to give $Y = X/(1 - X)$ (e.g. a value of 66% was expressed as 2 corresponding to a 66.66:33.33 = 2:1 ratio).

1.5 Combining Antibody Data from Two Plasma Units

1.5.1 Dealing with Replicate Measurements on a Given Plasma Unit

When multiple samples from the same donor plasma have been tested, the mean result was uploaded in REDCap. For the ADCC marker the averaging will be carried out on the scale of the proportion and then the ratio will be computed based on this average proportion.

1.5.2 Combination of Test Results from Different Plasma Units

All test values are entered into REDCap are measures of concentrations expressed on an absolute scale. Test results from the two different plasma units transfused to an individual will be combined as follows. A weighted sum of the two concentrations will be computed with weights based on the volume of the respective units. That is, we will compute an estiamte of the "total antibody dose" for the pooled sample as

```
Total Dose = (Vol. 1 ÃŮ Conc. 1 + Vol. 2 ÃŮ Conc. 2)
```

Example: Consider two units and the anti-RBD ELISA results with the first 235 ml having an O.D. of 0.25, and the second unit of 240 ml having an OD of 1.06. The overall OD is computed as

$$\text{Total Dose} = 235 \times 0.25 + 240 \times 1.06 = 313.15$$

This patient would be considered to have received the equivalent total dose of antibodies corresponding to a 500 ml plasma transfusion with an anti-RBD antibodies at $313.15/500 = 0.63$ optical density.

1.6 Log Transformation Before Analysis

Final marker values representing concentrations will be log-transformed ($\log_{10}$) before being entered in statistical analysis assessing their pharmacological effect. So with a concentration of 0.63 we will use $\log_{10}(0.63) = -0.20$.

1.7 Handling Missing Data

1.7.1 Missing Unit Volumes

When the volume of a plasma unit is missing, the median volume of convalescent plasma units from the same blood provider will be imputed.

1.7.2 Missing Markers for Particle Providers

When marker values are not available for a plasma provider, indivdiuals receiving plasma from that provider will be excluded from the related analysis.

1.7.3 Results missing for markers when measurements are expected

When a marker value was expected but it is not available, multiple imputation will be used to impute the missing value based on a linear model in which imputation is carried out based on a regression model for the missing marker given the values of the available markers. When multiple markers are missing then imputation will be carried out using multiple imputation based on chained equations (White et al. 2011) with 10 imputed samples. The imputation models will be based on the joint distributions of all four markers under consideration.

2 Statistical Analyses Involving Laboratory Data

As a preliminary analysis we will tabulate the availability of marker data by site, blood provider, and according to the type of the marker. A careful description of the units used to measure the markers will be carried out with any change of units carried out as required. Upon standardization of the scales the summary statistics will be computed for each marker by site, by provider, and overall. All of the analyses that follow will control for the provider as a factor variable to ensure that comparisons are made within providers. This variable is suppressed in the model statements that follow for convenience.

2.1 Regression Analyses Investigating Role of Markers in the CCP Arm

Preliminary analyses will be directed at modeling the relations between the laboratory profile of CCP and the probability of experiencing the primary outcome. This will be done initially in a univariate fashion and then via multiple regression with several markers. Let $Y_i = 1$ if individual i is intubated or dies within 30 days and let M_{ij} be the jth marker of interest, $j = 1, \ldots, J$, $i = 1, \ldots, n$. Let $\pi(M_{ij}) = P(Y_i = 1 | M_{ij})$ and

$$\log\left(\frac{\pi(M_{ij})}{1-\pi(M_{ij})}\right) = \alpha_0 + \alpha_1 M_{ij} \,,$$

be considered in the univariate analysis of marker j, $j = 1, \ldots, J$, and

$$\log\left(\frac{\pi(M_{i})}{1-\pi(M_{i})}\right) = \alpha_0 + \alpha_1 M_{i1} \cdots + \cdots \alpha_J M_{iJ}$$

be considered in the multivariate analysis where $M_i = (M_{i1}, \ldots, M_{iJ})'$ is the vector of markers. Tests for interactions will also be carried out to assess whether marker effects varying according to the value of other markers. Finally generalized additive models will be considered to give graphical plots of the relations between the marker values and the odds of response (Hastie and Tibshirani, 1990).

2.2 Regression Analyses Exploring the Effect-Modifying Role of Each Marker

The results of the antibody titre will give an indication of the amount of antibodies in the plasma transfused thereby enabling an investigation of the effect the amount of neutralizing antibodies has on the effect of CCP versus standard care. For each marker the value will be centered by subtracting the mean value among the plasma units transfused. For the primary binary response, a logistic regression model (McCullaugh and Nelder, 1989) will be fitted involving the treatment indicator (equalling 1 for those in the CCP arm) and an interaction between the treatment indicator and the centered marker. The coefficient of the treatment indicator will reflect the effect of CCP transfusion at the mean level of the marker, while the coefficient of the interaction term will reflect how this effect is modified according to the marker value in the transfused plasma. The details are given in what follows.

Let $Y_i = 1$ if individual i is intubated or dies within 30 days and let Z_i be their covariate vector, $i = 1, \ldots, n$. Let $\pi(Z_i) = P(Y_i = 1|Z_i)$ where $Z_i' = (1, X_i, X_i M_i)$ where X_i indicates randomization to the CCP arm and M_i is the marker level in the CCP.

$$\log\left(\frac{\pi(Z_i)}{1-\pi(Z_i)}\right) = \beta_0 + \beta_1 X_i + \beta_2 X_i M_i\,.$$

In order to relax the assumption of a linear effect-modifying role of the marker, generalized additive models (Hastie and Tibshirani, 1990) will be fitted for the effect of $X_i M_i$ with the results to give insight into critical threshold levels of the markers.

2.3 Multivariate Regression Analyses of Effect-Modifying Roles of Markers

Following the exploration of the effect-modifying roles of the J individual marker values, multivariate models will be fitted to investigate whether there are critical compbinations of levels across the markers which identify CCP which is particularly effective in reducing the odds of the primary response. For this we let M_{ij} denote the jth marker, $j = 1, \ldots, J$ and $Z_i = (X_i, M_{i1}, \ldots, M_{iJ})'$. Moreover we consider

$$\log\left(\frac{\pi(Z_i)}{1-\pi(Z_i)}\right) = \beta_0 + \beta_1 X_i + \beta_2 X_i M_{i1} \cdots + \cdots \beta_{J+1} X_i M_{iJ}\,.$$

and test the significance of the interaction terms $\beta_2, \ldots, \beta_{J+1}$. Again generalized additive models will be explored to provide graphical displays conveying the nature of the effect-modifying role of the marker.

2.4 Incorporating the Patient Marker Values

2.4.1 Exploring the Effect of Patient Marker Values

Here we consider patients in the CCP arm along but let M_{ij}^P denote the marker value for the jth marker measured in the patient, assumed to be centered. Let $Z_{ij} = (M_{ij}, M_{ij}^P)'$ for a particular marker under study. Here we consider interactions between the marker value in the transfused plasma and the marker value in the patient in models given by

$$\log\left(\frac{\pi(Z_{ij})}{1-\pi(Z_{ij})}\right) = \theta_0 + \theta_1 M_{ij} + \theta_2 M_{ij}^P + \theta_3 M_{ij} M_{ij}^P \,,$$

$j = 1, \ldots, J$.

2.4.2 Incorporating the Patient Marker Values - Effect Modifying Roles

Let M_{ij}^P denote the marker value for the jth marker measured in the patient, as opposed to the unit transfused, $j = 1, \ldots, J$. Additional analyses will introduce the patient markers denoted M_{ij}^P into the analyses, which are assumed to be centered values. Let $Z_{ij} = (X_i, M_{ij}, M_{ij}^P)'$ for a particular marker under study. Here we consider interactions between the marker value in the transfused plasma and the marker value in the patient in models given by

$$\log\left(\frac{\pi(Z_{ij})}{1-\pi(Z_{ij})}\right) = \gamma_0 + \gamma_1 X_i + \gamma_2 X_i M_{ij} + \gamma_3 X_i M_{ij}^P + \gamma_4 X_i M_{ij} M_{ij}^P \,.$$

Again multivariate models involving several markers can be considered if the above marker-specific analyses yield evidence that more than one marker may be critical in determining the effect of the convalescent plasma.

3 References

Hastie TJ (1992). *Generalized Additive Models.* Chapter 7, *Statistical Models in S*, Eds. JM Chambers and TJ Hastie, Wadsworth & Brooks/Cole.

Hastie T and Tibshirani R (1990). *Generalized Additive Models.* London: Chapman and Hall.

McCullaugh P and Nelder J (1989). *Generalized Linear Models.* London: Chapman and Hall.

Venables WN and Ripley BD (2002). *Modern Applied Statistics with S.* New York: Springer.

White IR, Royston P, and Wood AM (2011). Multiple imputation using chained equations: issues and guidance for practice. *Statistics in Medicine*, 30(4), 377-399.
