# Supplementary material for: Convalescent plasma for adults with acute COVID-19 respiratory illness (CONCOR-1): study protocol for an international, multicentre, randomized, open-label trial
Source: Trials. 2021 May 4;22:323. doi: 10.1186/s13063-021-05235-3 (PMC8094980; doi:10.1186/s13063-021-05235-3)
Supplement: Supplementary file 3 — Additional file 3. IDSMC Charter version 2.0, 03 Nov 2020. [file 13063_2021_5235_MOESM3_ESM.pdf]

Charter

Independent Data Safety Monitoring Committee (IDSMC) for CONCOR-1

CONvalescent Plasma for **CO**VID-19 **R**esearch Trial (**CONCOR-1 trial)**

Multicentre, randomized, open-label, superiority trial of COVID-19 CP vs. standard of care**.**

Version 2.0

November 3, 2020

1.0 Introduction

This Charter is for the Independent Data Safety Monitoring Committee (IDSMC) for the CONCOR-1 clinical trial. The primary objective of this study is to determine the impact of the early transfusion of COVID-19 convalescent plasma to hospitalized COVID-19 patients on the risk of intubation or death at 30 days, compared to the local standard of care. The trial is an investigator-initiated trial with independent funding and in kind contribution from both Canadian Blood Services and Hema-Quebec.

This Charter is a living document that may be revised by the IDSMC as required to facilitate their role in providing trial guidance, oversight, and protecting human subjects from avoidable risk. Such revisions will be subject to approval of the trial co-principal investigators (co-PI).

2.0 Role of the Independent Data Safety Monitoring Committee (IDSMC)

2.1 General Role and Responsibilities

The role of the IDSMC is to act in an independent, advisory capacity to the co-principal investigators and sponsor, providing guidance to help ensure:

- The protection of human subjects participating in the study;
- The proper conduct of the trial; and
- The ongoing scientific validity, integrity, and clinical and scientific relevance of the study.

The IDSMC will provide recommendations about continuing, modifying, and/or stopping the study based on considerations of treatment outcomes, patient safety, and trial futility as appropriate. In addition, the IDSMC may make observations or recommendations to the co-principal investigators and sponsor about, but not limited to, the following:

- Patient outcomes;
- Definitions of and responses to adverse events and patterns in adverse events;
- Benefit/risk ratio of procedures and participant burden;
- Amendments to the study protocol and consent forms;
- Participant safety.

Please see Section 6.0 for information regarding the communication of recommendations from the IDSMC.

The IDSMC functions independently and should not be approached by investigators unless responding to questions.

The role of the IDSMC will be completed once enrollment has been completed and all data has been reviewed.

2.2 Role of IDSMC in Initiation of Patient Enrollment

Prior to the initiation of patient enrollment, the IDSMC will review the study protocol, the trial design, and the analysis plan in the study protocol. Any formal amendments to the approved clinical protocol during the conduct of the study will also be reviewed by the IDSMC.

2.3 Role of IDSMC in Reviewing and Monitoring Adverse Events (AEs)

Adverse event data, including information regarding adverse events (AEs) and serious adverse events (SAEs), will be reported by investigators, as detailed in the protocol. Information regarding adverse events that meet the definition of Suspected Unexpected Serious Adverse Reactions (SUSAR) will be forwarded to the IDSMC chair within 7 days of receipt by the co-Principal Investigators. The IDSMC will review the reports and relay any comments, concerns, or recommendations based on safety data to the Sponsor within 14 days of receipt.

Tabulations of SAEs will be reviewed by the IDSMC at each meeting that occurs after the initiation of patient enrollment. Review of the safety data available will take place after 20 patients have been enrolled, and then regularly as required. The review will include data on study progress, enrollment, deaths, grade 3 and 4 SAEs including all transfusion reactions.

2.4 Role of IDSMC during conduct of study and Interim Analysis

As the study is ongoing, the IDSMC will review data related to study progress, as well as the efficacy and safety of the study. This data monitoring serves the purpose of an ongoing assessment of recruitment problems as well as the compatibility of the accumulating data with the assumptions made at study start. This will occur on a regular basis and will include a review of efficacy and safety data (blinded or unblinded as per IDSMC preference), and will give advice on the continuation, modification, or termination of the study. In addition, if novel scientific data on the efficacy or safety of convalescent plasma is published during the conduct of the trial, the IDSMC will review the reported medical evidence and provide recommendations to the co-principal investigators and the sponsor regarding implications for the trial conduct. The IDSMC will also receive data regarding antibody titres from donor plasma samples as this data becomes available.

The CONCOR-1 trial design incorporates an interim analysis when the primary outcome data (intubation or in-hospital death) are available for 50% of the intended sample size. This will facilitate a detailed review of safety data and will involve a formal assessment of whether there is sufficient evidence that the study should be stopped early to conclude superiority of convalescent plasma compared to standard of care. Also at the interim analysis a one-time sample size re-estimation calculation will take place. Upon reviewing the results of the interim analysis and the revised sample size requirements, the IDSMC will consider the risk-benefit balance for participants. If the IDSMC determines that the risk-benefit balance is no longer acceptable, the IDSMC will recommend either: (1) immediate steps to be taken to restore an

appropriate risk-benefit balance for participants; or (2) that the trial be terminated for potential harm. At the time of the first sample size re-estimation the IDSMC may consider any of the following recommendations:

- To continue the trial as planned until 1200 patients have completed the study,
- To stop the trial for demonstrated superiority at the interim analysis,
- To stop the trial at the interim analysis if the revised sample size (from blinded sample size re-estimation) makes the resultant study infeasible,
- To continue the trial with a modified sample size.

The IDSMC deliberations will be guided by the trial design as defined in the study protocol and statistical analysis plan, although the IDSMC may make recommendations that deviate from the trial design if necessary to protect patient safety, or based on considerations of treatment efficacy, harm, or trial futility. IDSMC recommendations will be communicated to the sponsor and co-principal investigators.

3.0 Membership

The IDSMC will consist of a minimum of six voting members with, collectively, expertise in the field of transfusion medicine, critical care medicine, methodology/epidemiology, infectious diseases and cardiology. The initial membership is given in **Appendix 1**. In the event that an IDSMC member is unable to complete their duties, a suitable replacement will be identified by the IDSMC Chair in cooperation with the PI. The IDSMC may add an additional member if they determine it is necessary to have additional expertise in a particular area. An independent and unblinded statistician will prepare materials for review by the IDSMC.

A quorum will require two IDSMC members (voting or non-voting) in addition to the chair. In an extraordinary circumstance in which the chair is unable to participate in IDSMC deliberations, and an urgent IDSMC meeting is required to ensure research subject safety, an acting IDSMC chair may be selected by the IDSMC members from the pool of existing IDSMC members.

4.0 Conflict of interest

All IDSMC members must be free of substantive conflicts of interest, including financial, scientific, or personal conflicts of interest, with respect to the clinical study, the co-principal and co-investigators, and Canadian Blood Services/HemaQuebec. All potentially relevant conflicts of interest must be disclosed by potential IDSMC members prior to appointment. Whether a potential conflict of interest is, in fact, substantive and disqualifying will be determined by consensus of the co-Principal Investigators and the IDSMC chair. In particular, no IDSMC member should have a financial or non-financial interest in the outcome of the study. Examples of such conflicts of interest include:

- Being an employee of the Sponsor or having financial relations with the Sponsor (or its affiliates) outside the scope of scientific consulting;
- Being an investigator in the trials overseen by this Charter;
- Planned authorship in publications of study results from the study overseen by this Charter; and
- Serving on another IDSMC (or similar committee) for clinical trials with the same intervention, but a different Sponsor.

It is the responsibility of the Chair to request new conflicts of interest be declared at each meeting. If any are identified, they should then be reviewed with the PIs.

5.0 IDSMC Meetings

5.1 Calling of Meetings

A meeting of the IDSMC may be called at any time by the chair of the IDSMC or the study co-Principal Investigators. If the IDSMC chair and others disagree regarding the need for an IDSMC meeting, the opinion of the IDSMC chair will prevail.

The purpose of the first meeting will be to review and discuss this Charter, to provide an overview of the trial protocol(s) and activities and to determine the frequency of IDSMC meetings. Subsequent meetings will be conducted as specified in section 2.3.

5.2 Meeting Formats

IDSMC meetings will be held by telephone or video conference. IDSMC sessions may be either *open* or *closed* and, in general, each IDSMC meeting will include both an open and a closed session. During an open session, the co-Principal Investigators or co-investigators and/or other interested parties may be present. During open sessions, only non-confidential information that does not threaten the integrity or feasibility of the study will be discussed, such as general information regarding patient enrollment, amendments, and modifications to the protocol, and external information that may influence the conduct of the study.

Closed IDSMC sessions may only include full voting members of the IDSMC, the independent statistician preparing the interim reports, and personnel whose presence is explicitly determined to be required by a majority vote of the IDSMC. All matters and information, affecting the safety, ethics, and scientific validity and integrity of the study may be discussed during closed sessions. All formal recommendations considered by the IDSMC will be discussed during closed sessions.

Voting on recommendations will follow Roberts' Rules of Order (Robert's Rules of Order Newly Revised (10th Edition) by Henry M. Robert III, William J. Evans (Editor), Daniel H. Honemann

(Editor), Thomas J. Balch (Editor), Sarah Corbin Robert, Henry M. Robert III, General Henry M. Robert).

At the conclusion of the closed session, if any, participants in the open session will be re-convened so that the IDSMC chair may provide a summary of the IDSMC's recommendations, if applicable, providing an opportunity for study investigators or Sponsor to obtain clarification regarding the recommendations.

5.3 Minutes

The minutes of open and closed sessions will be prepared by the IDSMC chair or his designee. The minutes of open sessions including recommendations, if applicable, may be distributed freely (e.g., to other investigators, Sponsor representatives), as deemed appropriate by the Sponsor. The minutes of closed sessions may only be distributed to personnel present at those sessions, until after the formal termination of the study. Once the study is formally terminated and all statistical analyses have been completed, the minutes of closed sessions will be released by the IDSMC chair.

6.0 IDSMC Communications and Recommendations

Within 15 days of each IDSMC meeting, the IDSMC chair will communicate the results of the meeting to the co-Principal Investigators and Sponsor. IDSMC communication(s) and/or recommendation(s) will be transmitted in written format to the co-Principal Investigators and the Sponsor who will review the information and, as appropriate, forward it to other personnel. The actual transmission of IDSMC communications or recommendations may occur by electronic mail or by facsimile. The rationale for an IDSMC recommendation may or may not be given, consistent with maintaining the scientific integrity of the study.

If, in the opinion of the IDSMC, rapid communication of information or recommendations from the IDSMC to trial investigators is required to ensure the safety of study participants or the integrity of the trials, then the IDSMC chair will communicate this to the co-Principal Investigators and/or other interested parties during the final open session of a IDSMC meeting. Preferably then, but at the latest within 5 working days, the IDSMC chair will prepare a recommendation for communication to trial investigators, which, after mutual agreement with the Principal Investigator and/or other interested parties will be forwarded to all participating investigators. It is expected that trial investigators will not communicate with IDSMC members about the study directly, except when making presentations or responding to questions at IDSMC meetings or during conference calls.

If the IDSMC does not identify any safety or other protocol-related concerns during a meeting then, within 15 days after receiving the written summary of the IDSMC meeting the co-Principal Investigators and/or Sponsor will prepare a statement or Summary Report for distribution to the clinical centers that will state that:

- A review of patient outcomes, adverse events, and information relating to study performance (e.g., data timeliness, completeness, and quality) across all centers took place on a given date; and
- The IDSMC recommended that the study continue without modification of the protocol or informed consent.

If concerns are identified, the Summary Report will include the IDSMC's recommendation, instructions from the co-Principal Investigators and/or other interested parties, if any, and the remaining Summary Report content will be modified appropriately.

7.0 Contractual Considerations

Members of the IDSMC, including its chair, will not be compensated for their time spent on IDSMC activities.

8.0 Confidentiality

The IDSMC members agree to keep completely confidential and not make accessible to third parties any confidential information, business secrets and other proprietary information furnished by the other party pursuant to the establishment of the IDSMC. This provision shall be in force during and after the termination of the participation in the IDSMC. The terms and conditions are more particularly set out in a Confidentiality Agreement already signed by each IDSMC member. The Confidentiality Agreement is a binding part of this Agreement. Confidential Information means all information relating to study with the Sponsor disclosed by or on behalf of the Sponsor, whether disclosed in writing, verbally or by any other means and regardless of the date it was disclosed.

9.0 Signatures of IDSMC Members

By signing this present document, I declare to have no conflict of interest, as outlined in Section 4.0, and to adhere to the procedures of this Charter.

Keyvan Karkouti, MD (Chair)	___________________ Place/Date	___________________ Signature
Robert Fowler, MD	___________________ Place/Date	___________________ Signature
Meghan Delaney, MD	___________________ Place/Date	___________________ Signature

George Tomlinson, PhD	___________________<br>Place/Date	___________________<br>Signature
Darryl Davis, MD	___________________<br>Place/Date	___________________<br>Signature
Boris Juelg, MD	___________________<br>Place/Date	___________________<br>Signature

Independent (non-voting) statistician

Na Li, PhD	___________________<br>Place/Date	___________________<br>Signature

10.0 Approval by the co-Principal Investigators

Donnie Arnold, MD	___________________<br>Place/Date	___________________<br>Signature
Philippe Bégin, MD, PhD	___________________<br>Place/Date	___________________<br>Signature
Jeannie Callum, MD	___________________<br>Place/Date	___________________<br>Signature

Appendix 1: IDSMC Membership Contact Information

IDSMC Members Contact Information and Addresses

Keyvan Karkouti (Chair)
Toronto General Hospital
3 Eaton North- 200 Elizabeth Street
Toronto, Ontario, Canada
Keyvan.karkouti@uhn.ca

Robert Fowler
Sunnybrook Health Sciences Centre
2075 Bayview Ave, Rm D478
Toronto, Ontario, Canada
Rob.fowler@sunnybrook.ca

Meghan Delaney (Chair designate if Chair unable to serve duties due to illness)
Children's National,
Pathology and Laboratory Medicine
111 Michigan Avenue NW
Washington, DC
20010
mdelaney2@childrensnational.org

Boris Juelg
Massachusetts General Hospital
400T-7-759
55 Fruit Street
Boston, Massachusetts, USA
02114
bjulg@mgh.harvard.edu

George Tomlinson
Toronto General Hospital
10 Eaton North Rm 235- 200 Elizabeth Street
Toronto, Ontario, Canada
George.tomlinson@utoronto.ca

Darryl Davis
University of Ottawa Heart Institute
40 Ruskin Street
Ottawa, Ontario, Canada
dadavis@toh.ca

Na Li
McMaster Centre for Transfusion Research
McMaster University
HSC 3H50
1280 Main St W
Hamilton, ON L8S 4K1
Lin18@mcmaster.ca
