# Supplementary material for: Convalescent plasma for adults with acute COVID-19 respiratory illness (CONCOR-1): study protocol for an international, multicentre, randomized, open-label trial
Source: Trials. 2021 May 4;22:323. doi: 10.1186/s13063-021-05235-3 (PMC8094980; doi:10.1186/s13063-021-05235-3)
Supplement: Supplementary file 4 — Additional file 4. Clinical Trials Ontario Consent Form Template (English) Version 1.2, 13 Apr 2020 & Consent Form Template (French- for Quebec sites) Version 1.1, 12 Apr 2020. [file 13063_2021_5235_MOESM4_ESM.pdf]

Informed Consent Form for Participation in a Research Study

**Study Title**: **CONvalescent Plasma for COVID-19 Research Trial (CONCOR-1)**

**Study Doctors**: Dr. ______; Department of _________ (Phone:__________)
Dr. ______; Department of _________ (Phone:__________)
insert name, department and telephone or pager number

**Sponsor:** Dr. Donald Arnold; McMaster University Medical Centre; Hamilton Health Sciences Centre

**Funders:** Canadian Blood Services Program Support Award- McMaster University
Canadian Blood Services Program Support Award – University of Toronto
Sunnybrook Foundation
Fonds de recherche du Québec – Santé [Québec Research Fund – Health]

INTRODUCTION

As a Substitute Decision Maker, you are being asked to provide informed consent on behalf of a person who is unable to provide consent for him/herself. If the participant gains the capacity to consent for him/herself, your consent for them will end. Throughout this form, "you" means the person you are representing.

You are being invited to participate in a clinical trial (a type of study that involves research). You are invited to participate in this trial because you are admitted to the hospital with COVID-19 related illness and are requiring oxygen. This consent form provides you with information to help you make an informed choice. Please read this document carefully and ask any questions you may have. All your questions should be answered to your satisfaction before you decide whether to participate in this research study. Please take your time in making your decision. You may find it helpful to discuss it with your friends and family.

Taking part in this study is voluntary. You have the option to not participate at all or you may choose to leave the study at any time. Whatever you choose, it will not affect the usual medical care that you receive outside the study.

IS THERE A CONFLICT OF INTEREST?

Describe any conflict of interest that exists or may appear to exist as it relates to any of the investigators, study staff or member of their immediate family. A conflict of interest exists if there is a potential benefit to the investigator(s), study staff or member of their immediate family beyond the professional benefit from academic achievement or presentation of the results. Examples include, but are not limited to, speaker's fees, travel assistance, consultant fees, honoraria, gifts, and intellectual property rights such as patents. A declaration of conflict of interest should include the identity of the person with the conflict of interest, the type of incentive or inducement, and its source. See examples below.

Canadian Blood Services (CBS) and Héma-Québec (HQ) are the two blood suppliers in Canada. These corporations will be working with our research team to bring convalescent plasma to hospitals under a clinical trials application (CTA) through Health Canada. Neither CBS nor HQ have say in how the study is run at the hospitals and do not have access to any patient information. The investigators and members of funding/supporting organizations have an interest in completing this study. Their interests should not influence your decision to participate in this study. You should not feel pressured to join this study.

WHAT IS THE BACKGROUND INFORMATION FOR THIS STUDY?
Each participating site must ensure that the standard or usual treatment described in below matches the standard of care at that site. Site specific differences must be reflected in the Centre Initial Application and the site-specific consent form.

There is no known cure for COVID-19 but the standard or usual treatment right now is supportive care such as supplemental oxygen. This study is being run Canada-wide as a national effort to determine if there is benefit to patients in hospital with COVID-19-related illness to receiving an experimental treatment called convalescent plasma.

Plasma is the liquid part of the blood that contains proteins and antibodies. Antibodies are formed when the body encounters infection and helps to fight off the infection. Convalescent plasma is plasma that has been collected from people who have recovered from an infection and now have evidence of high numbers of antibodies, but no longer have the infection. Convalescent plasma has been given to patients in the past with other types of Coronaviruses. Other countries are starting to use convalescent plasma to treat patients with COVID-19 and while initial results look promising, the numbers of patients treated are very small.

The convalescent plasma used in this study will be provided by Canadian Blood Services or Héma-Québec, the government agencies that collect, process, and supply all blood products in Canada. Health Canada, the regulatory body that oversees the use of investigational products and drugs in Canada has allowed convalescent plasma to be used in this study.

WHY IS THIS STUDY BEING DONE?
The purpose of this study is to compare the treatment of patients admitted with COVID-19 related illness with convalescent plasma compared to standard of care. This pan-Canadian clinical trial has the potential to improve patient outcomes and reduce the burden on health care resources including reducing the need for ICU beds and ventilators.

WHAT OTHER CHOICES ARE THERE?
You do not have to take part in this study in order to receive treatment or care. If you choose not to participate in this study, you will continue to receive standard of care to support you during your hospital admission. You may also be enrolled in other studies.

HOW MANY PEOPLE WILL TAKE PART IN THIS STUDY?
This study is being conducted at approximately 40 hospitals in Canada. It will eventually include approximately 1200 patients. The study should take about 18 months to complete.

WHAT WILL HAPPEN DURING THIS STUDY?/ WHAT IS THE STUDY INTERVENTION?
If you choose to participate, you will be randomly assigned (like flipping a coin) to receive convalescent plasma in addition to standard of care or no convalescent plasma. There is a 2/3 chance that you will receive convalescent plasma, and a 1/3 chance that you will not. Neither you, your doctor, or the study staff can influence this assignment. You may also be enrolled in other studies. There is a chance that you choose to participate in the study and we do not have a compatible unit available for your blood type. If

this happens, we will re-approach you if a compatible unit becomes available and you are still eligible for the study.

**Procedure:**
If you are randomized to receive convalescent plasma, you will receive an intravenous transfusion of approximately 500 mL of yellow-appearing fluid over approximately 4 hours. A nurse will monitor your vital signs during and after the transfusion. If you are randomized to standard of care that means the clinical team provides supportive management that is directed by patient-symptoms, severity and local practice.

You are still allowed to participate in this trial if you are pregnant. There are no known reproductive risks associated with participating in this study. While few pregnant women have reported to present with complications related to COVID-19, data from current COVID-19 hospital wards show that they have a similar risk of disease progression as elderly patients. The same risk/benefit considerations apply to pregnant and younger patients as compared to the elderly .Pregnant patients will not be excluded from the study if they meet eligibility criteria. If you are or think you may be pregnant, you are advised to inform a research staff member.

As part of your routine hospital care, you may have laboratory and radiological tests performed. You will not undergo any additional procedure as part of this study, unless you require a blood test to determine compatibility with the blood product. If you do require this blood sample, it is equivalent to less than half a tablespoon. We will be collecting this information from your medical chart along with your general well-being during the follow-up period. No other aspect of your care will be modified.

You will be told of any new findings that may affect your willingness to continue to participate in this study in a timely manner.

<u>WHAT ARE THE RESPONSIBILITIES OF STUDY PARTICIPANTS/ HOW LONG WILL PARTICIPANTS BE IN THE STUDY?</u>
If you choose to participate in this study, you will be giving research staff permission to contact you for follow up 30 and 90 days from your involvement in the study. This will take no more than 5-10 minutes and is typically done over the phone. If you are pregnant and/or become pregnant during the trial, we will contact you after your delivery to check on you and your baby.

<u>CAN PARTICIPANTS CHOOSE TO LEAVE THE STUDY?</u>
If you have any concerns, please do not hesitate to discuss them with us. Withdrawal from the study is possible at any time, which will not have any impact on the quality of your care. If you decide to withdraw from the study, no additional information will be collected. The study information collected prior to your withdrawal will still be used to help answer the research question. Please let your study doctor know.

<u>CAN PARTICIPATION IN THIS STUDY END EARLY?</u>
The study doctor may stop your participation in the study early, if:
- You are unable to tolerate the study intervention

If you are removed early from this study, the study team will still ask permission to collect data from your chart and continue to follow you for the 90-day duration of the study.

WHAT ARE THE RISKS OR HARMS OF PARTICIPATING IN THIS STUDY?

Plasma is a blood product that is collected from another person. Convalescent plasma has the same risk of any plasma transfusion:

1. Allergic or hypersensitivity reaction. This can include redness of skin, itchiness, hives, wheezing, tightness of chest and low blood pressure. In rare cases it can lead to anaphylaxis (a severe allergic reaction) or shock (<1 in 20,000).
2. Transfusion-related acute lung injury – a rare syndrome that causes sudden respiratory distress within 6 hours of a transfusion (<1 in 10,000)
3. Transfusion-related circulatory overload- is a transfusion reaction that can occur due to a rapid transfusion of a large volume of blood products (<1%).
4. Infection from blood products. Whenever medical products are derived from human blood or plasma, there is a possibility of transmitting infective agents, including viruses and bacteria. The blood suppliers (Canadian Blood Services and Héma-Québec) have very strict protocols that they follow to prevent this from happening (<1 in 1 million).

There is also a possibility of risks that we do not know about and have not been recorded in medical literature or seen in study participants to date. You will be very closely monitored during your time at the hospital and any complications will be quickly identified and managed.

WHAT ARE THE BENEFITS OF PARTICIPATING IN THIS STUDY?

Convalescent plasma is an experimental treatment. It may help you to fight the COVID-19 virus but we do not know yet if there is benefit. Information learned from this study will help further medical and scientific knowledge.
I understand that my information and samples will be used for research purposes only and this research may also have commercial uses that benefit society. You will not receive compensation for these developments.

HOW WILL PARTICIPANT INFORMATION BE KEPT CONFIDENTIAL?

**Note:** If there will be disclosure of personal identifiers, i.e., disclosed on any research-related information/documents including samples or scans, or as part of the unique identifier, these disclosures must be justified in the REB application and approved. Please ensure that you are aware of institutional and REB policies with respect to the disclosure of personal identifiers.

Protecting and maintaining confidentiality of your medical information is very important to us. We will remove all personal identifying information such as name, address, date of birth etc. from all documents and information collected and you will be given a unique study number.

The study doctor and his/her study team will look at your personal health information and collect only the information they need for the study.

Personal health information (PHI) is any information that could identify you and includes your:

- name,
- date of birth,
- Phone number, email
- New or existing medical records, which includes types, dates and results of medical tests or procedures.

The study doctor will keep any personal health information about you in a secure and confidential location for a minimum of 25 years as required by research standards. A list linking your study number with your name will be kept by the study doctor in a secure place, separate from your study file.

Representatives of Clinical Trials Ontario, a not-for-profit organization, may see study data that is sent to the research ethics board of record for this study. Your name, address, email, or other information that may directly identify you will not be used.

In case any adverse events are believed to be related to this research study, PHI will be removed, and the appropriate data will also be sent to relevant regulatory agencies, Canadian Blood Services or Héma Québec (suppliers of the plasma)

The study team or the people/groups listed below will be allowed to look at your records.
The following people or organizations may come to the hospital to look at the study records and at your personal health information to check that the information collected for the study is correct and to make sure the study is following proper laws and guidelines:

- Representatives of Sunnybrook Health Sciences Centre Research Ethics Board the research ethics board of record who oversee the ethical conduct of research studies at the hospital
- Study Sponsor (Dr. Donald Arnold) or one of his representatives from Hamilton Health Sciences
- External data monitors and or auditors to ensure quality and accuracy
- Government regulatory authorities (e.g. Health Canada)
- This institution and affiliated sites, to oversee the conduct of research at this location

**Study Information that Does Not Identify You**
Information about you will have a code and will not show your name or address, or any information that directly identifies you. All information collected during this study, including your PHI, will be kept confidential and will not be shared with anyone outside the study unless required by law. You will not be named in any reports, publications, or presentations that may come from this study.

After all your personal identifiers have been removed, the research related data collected will be stored on a secured research electronic data capture (REDCap) web-server. This method of data storage meets all Canadian privacy laws.

Studies involving humans sometimes collect information on race and ethnicity as well as other characteristics of individuals because these characteristics may influence how people respond to different interventions. Providing information on your race or ethnic origin is voluntary.

If you prefer, we may communicate via email to forward the informed consent form for you to read and sign, especially in instances when the patient has left the hospital prior to signing the consent form or if the SDM does not live close by. Communication via e-mail is not absolutely secure. We do not recommend that you communicate sensitive personal information via e-mail.

If the results of this study are published, your identity will remain confidential. It is expected that the information collected during this study will be used in analyses and will be published/presented to the scientific community at meetings and in journals. This information may also be used as part of a submission to regulatory authorities around the world to support the approval of the study intervention.

Even though the likelihood that someone may identify you from the study data is very small, it can never be completely eliminated.

A copy of the consent form that you sign to enter the study will be included in your health record/hospital chart.

WILL FAMILY DOCTORS/HEALTH CARE PROVIDERS KNOW WHO IS PARTICIPATING IN THIS STUDY?
Your family doctor/health care provider will not be informed that you are taking part in the study. You can choose to let your family doctor/health care provider know, if you like.

WILL INFORMATION ABOUT THIS STUDY BE AVAILABLE ONLINE?
A description of this clinical trial will be available on http://www.clinicaltrials.gov. This website will not include information that can identify you. The site will include a summary of the study and eventually the results. You can search this site at any time.

WHAT IS THE COST TO PARTICIPANTS?
Participation in this study will not involve any additional costs to you or your private health care insurance.

ARE STUDY PARTICIPANTS PAID TO BE IN THIS STUDY?
Each participating site must ensure that the information below matches the compensation/reimbursement provided at that site. Site specific differences must be reflected in the Centre Initial Application and the site-specific consent form.
You will receive no payment or incur any costs upon agreeing to remain in this study.

WHAT ARE THE RIGHTS OF PARTICIPANTS IN A RESEARCH STUDY?
If you were harmed as a direct result of taking part in this study, all necessary medical treatment will be made available to you at no cost.

By signing this form you do not give up any of your legal rights against the investigators, sponsor or involved institutions for compensation, nor does this form relieve the investigators, sponsor or involved institutions of their legal and professional responsibilities.

You have the right to receive all information that could help you make a decision about participating in this study. You also have the right to ask questions about this study and your rights as a research participant, and to have them answered to your satisfaction, before you make any decision. You also have the right to ask questions and to receive answers throughout this study.

You will be given a copy of this signed and dated consent form.

WHOM DO PARTICIPANTS CONTACT FOR QUESTIONS?

If you have questions about taking part in this study, or if you suffer a research-related injury, you can talk to your study doctor, or the doctor who is in charge of the study at this institution. That person is:

______________________________ ___________________________

Name Telephone

If you have questions about your rights as a participant or about ethical issues related to this study, you can talk to someone who is not involved in the study at all. That person is:

Sunnybrook Research Ethics Board 416 480 6100 x 88144 ___________________________

Name Telephone

SIGNATURES

Study Title: **CONvalescent Plasma for COVID-19 Research Trial (CONCOR-1)**

Name of Participant: ______________________________________

Participant/Substitute decision-maker

By signing this form, I confirm that:

- This research study has been fully explained to me and all of my questions answered to my satisfaction
- I understand the requirements of participating in this research study
- I have been informed of the risks and benefits, if any, of participating in this research study
- I have been informed of any alternatives to participating in this research study
- I have been informed of the rights of research participants
- I have read each page of this form
- I authorize access to my personal health information, medical record and research study data as explained in this form
- I have agreed, or agree to allow the person I am responsible for, to participate in this research study This informed consent document may be placed in my medical records

Future Research

- I also give my permission for any left-over samples to be stored for REB approved future COVID-19 related research testing at Hamilton Health Sciences or local institution. I understand that no additional time or hospital visits are required and that the samples will be kept for 15 years.

- ☐ Yes ☐ No

| ____________________________ | __________________________ | ________________ |
|---|---|---|
| Name of Participant/SDM (print) | Signature | Date |

**ASSISTANCE DECLARATION**

[The Assistance Declaration provides a mechanism for potential participants who are unable to read the informed consent form (i.e. illiterate, blind or for who English is their second language) to participate in research studies.]

Was the participant assisted during the consent process? ☐ Yes ☐ No

☐ The consent form was read to the participant/substitute decision-maker, and the person signing below attests that the study was accurately explained to, and apparently understood by, the participant/substitute decision-maker.

☐ The person signing below acted as a translator for the participant/substitute decision-maker during the consent process. He/she attests that they have accurately translated the information for the participant/substitute decision-maker, and believe that that participant/substitute decision-maker has understood the information translated.

| ________________________ | _____________________ | _____________________ |
|---|---|---|
| Name of Person Assisting (print) | Signature | Date |

Person obtaining consent

By signing this form, I confirm that:

- This study and its purpose has been explained to the participant named above and all questions asked by the participant have been answered.
- I will give a copy of this signed and dated document to the participant

| | | |
|---|---|---|
| ______________________________ | ________________________ | _______________________ |
| Name of Person obtaining consent (print) | Signature | Date |

APPROUVÉ PAR LE COMITÉ D'ÉTHIQUE
16 AVRIL 2020
MP-21-2020-2863
CHU SAINTE-JUSTINE

CHU Sainte-Justine
Le centre hospitalier universitaire mère-enfant

Université de Montréal

**INFORMATION SUR LA RECHERCHE ET FORMULAIRE DE CONSENTEMENT**

**Titre de l'étude :** Essai clinique multicentrique, adaptatif, randomisé, ouvert et contrôlé sur la sécurité et l'efficacité du plasma de convalescence pour le traitement de la COVID-19 chez les patients hospitalisés

**Promoteurs de l'étude :** Dr Philippe Bégin, Centre Hospitalier Universitaire Sainte-Justine
Dr Donal Arnold, Université McMaster

**Fournisseurs du produit** : Héma-Québec
La Société Canadienne du Sang

**Sources de financement:** Centre Hospitalier Universitaire du CHU Sainte-Justine
Fonds de Recherche du Québec en Santé

**Investigatrice principale à Sainte-Justine:** Geneviève Du Pont-Thibodeau

Dans ce document, "vous" réfère à "vous" ou "vous/votre adolescent" ou encore « la personne adulte pour qui vous consentez ».

## POURQUOI ÊTES-VOUS INVITÉ À PARTICIPER À CE PROJET DE RECHERCHE ?

Vous avez été invité à participer à ce projet de recherche qui vise à déterminer l'efficacité d'une transfusion de plasma provenant de patients guéris d'une COVID-19 (plasma convalescent) pour traiter les patients hospitalisés pour COVID-19.

Les essais cliniques incluent uniquement des participants volontaires. Avant que vous décidiez si vous voulez participer ou non à cette étude, il est important que vous compreniez l'objectif de la recherche, ce que l'étude implique, quels sont les bénéfices, risques et inconforts potentiels qui y sont associés, et comment vos renseignements seront utilisés.

Compte-tenu des règles d'isolement en vigueur en raison de la pandémie de COVID-19, les informations au sujet de ce projet vous seront présentées oralement par téléphone. Certains termes peuvent ne pas vous être familiers. N'hésitez donc pas à poser des questions s'il y a quoi que ce soit que vous ne comprenez pas ou si vous souhaitez plus d'informations.

**Pour les patients de 16 ou 17 ans**, nous encourageons les parents à discuter de l'étude avec leur adolescent et de prendre la décision conjointement avec celui-ci. Vous pouvez aussi

discuter de l'étude avec les membres de votre famille, vos amis, et votre propre médecin si vous le souhaitez.

**Si vous consentez pour un proche adulte qui est incapable de consentir en ce moment à cause de la maladie**, nous vous encourageons à lui parler de l'étude dès que cela devient possible. Sachez que votre proche sera informé et pourra donner son consentement avec l'équipe de recherche si cela devient possible.

**Si vous consentez pour un majeur inapte**, veuillez discuter de l'étude à un niveau de sa capacité à comprendre.

Etant donné le contexte de la pandémie, si vous décidez de prendre part à l'étude, votre consentement verbal sera obtenu. Une copie de ce formulaire de consentement sera déposée dans votre dossier médical et vous devrez le signer au moment de votre congé de l'hôpital ou lorsque vous ne serez plus en isolement.

**EN QUOI CONSISTE L'ÉTUDE ?**

Tel que mentionné plus tôt, le projet de recherche vise à tester l'efficacité et la sécurité de la transfusion de plasmas convalescents pour traiter les patients hospitalisés pour COVID-19.

Le plasma est la partie liquide du sang, sans les globules blancs et les globules rouges, qui contient les anticorps. Héma-Québec recueille actuellement des dons de plasma chez des patients guéris depuis quatorze jours de la COVID-19. C'est le moment à partir duquel le corps produit le plus d'anticorps contre le virus. Le traitement consiste à transfuser ces plasmas chez les personnes infectées pour que celles-ci n'ayant pas encore fabriqué ces anticorps soit protégées par ceux du donneur.

Il s'agissait d'une approche courante à l'époque des grandes épidémies de rougeole ou de grippe, avant le développement de vaccins. Ce traitement demeure expérimental puisque son efficacité n'a pas été démontrée pour la COVID-19, cependant Santé Canada en a approuvé l'utilisation dans cette étude.

**COMBIEN DE PERSONNES PARTICIPERONT AU PROJET DE RECHERCHE ?**

Nous visons le recrutement de plus de 800 patients qui seront recrutés dans près de 40 centres à travers le Canada.

**COMBIEN DE TEMPS DURERA LA PARTICIPATION À CE PROJET DE RECHERCHE ?**

Les patients de l'étude seront suivis pour une durée de 4 semaines. Les patients qui obtiennent leur congé durant cette période seront suivis par appels téléphoniques.

**COMMENT SE DEROULERA LE PROJET DE RECHERCHE ?**

- **<u>Éligibilité:</u>**

La première étape est de confirmer votre éligibilité pour participer à l'étude et s'assurer que vous n'avez pas de contre-indication à recevoir le plasma convalescent.

- **Randomisation:**
  Après l'obtention de votre consentement et la confirmation de votre éligibilité, vous serez assigné au hasard dans un des deux groupes de traitement (comme en tirant à pile ou face).

  Vous serez alors assigné soit au groupe contrôle qui reçoit les traitements habituels ou au groupe expérimental qui recevra une transfusion de plasma convalescent en plus des traitements habituels. Vous avez 2 chances sur 3 d'être dans le groupe qui recevra le plasma convalescent.

- **Transfusion de plasma convalescent:**
  Les participants dans le groupe expérimental recevront une transfusion d'environ 500mL (1/2 litre) en deux sacs de plasma convalescent. La dose sera adaptée pour les enfants pesant moins de 50kg (10mL/kg).

  La transfusion est administrée par voie intraveineuse (dans une veine) en respectant les règles d'administration de produits sanguins en vigueur. Votre état sera surveillé tout au long de la transfusion par du personnel médical et/ou de recherche.

- **Accès à votre dossier médical**
  L'équipe de recherche consultera votre dossier médical pour obtenir les informations pertinentes à cette recherche.

- **Questionnaire de qualité de vie**
  Avant la randomisation et à la fin de l'étude 30 jours plus tard. Nous vous ferons passer verbalement un questionnaire de la qualité de vie qui consiste en 5 questions à choix multiples.

- **Analyses de laboratoire**
  Pour éviter des prises de sang supplémentaires, des analyses de laboratoire seront réalisées à 2 reprises, avant et après la transfusion, en utilisant les restants des échantillons déjà prélevés dans le cadre de vos soins. Ces derniers serviront à mesurer la quantité de virus dans le sang et les anticorps contre ce dernier.

- **Suivi:**
  Durant votre hospitalisation, votre état de santé sera documenté dans un formulaire de recherche pour une durée maximale de 30 jours. Si vous avez eu votre congé de l'hôpital, un suivi sera effectué par téléphone pour documenter votre état de santé.

## QUELS SONT LES RISQUES POSSIBLES ?

**Quels sont les risques associés au plasma convalescent?**

La transfusion de plasma convalescent s'associe au même risque que toute transfusion sanguine. Ceci peut inclure une douleur au site de perfusion, une sensation de froid, des tremblements ou des frissons, de la fièvre, des démangeaisons ou des éruptions cutanées, une difficulté à respirer ou des réactions allergiques plus graves (anaphylaxie). Le risque de décès suite à une transfusion de plasma est de 1 sur 450,000. Il n'y a pas de risques spécifiques reliés à votre groupe d'âge.

Il existe également un risque de transmission d'infections par le sang. Des procédures de dépistage et le traitement des plasmas réalisés par Héma-Québec permettent de réduire ce risque.

Chez certains patients, il est possible que l'administration du volume de liquide (plasma) contribue à déstabiliser votre état si vous êtes déjà atteint d'une insuffisance cardiaque ou rénale. Dans ces cas, il est possible d'allonger la durée de la perfusion et d'espacer l'administration des 2 sacs de plasma.

Puisqu'il s'agit d'une approche expérimentale, il est également possible que le traitement ne fonctionne pas ou qu'il aggrave l'inflammation causée par l'infection.

Enfin, nous ne savons pas à l'heure actuelle si ce traitement pourrait avoir un impact sur la quantité d'anticorps qui seront produits naturellement par votre corps contre le virus de la COVID-19 suite à l'infection.

Si vous deviez subir quelque préjudice que ce soit par suite de l'administration du plasma convalescent à l'étude ou de toute procédure reliée à ce projet de recherche, vous recevrez tous les soins et services requis par votre état de santé.

**Y a-t-il des risques si je suis enceinte ou si j'allaite durant l'étude ?**

L'impact du traitement convalescent sur la grossesse et l'allaitement n'a jamais été étudié. La transfusion de plasma régulier est fréquemment utilisé en grossesse pour prévenir les saignements. Parmi les 19 cas rapportés à ce jour de patients avec COVID-19 sévère traités par transfusion de plasma convalescent, il y avait une patiente enceinte qui aurait bien répondu au traitement. En théorie, les anticorps transfusés devraient passer la barrière du placenta et protéger également le fœtus, (assumant que le traitement s'avérait efficace). Certains anticorps du donneur pourrait également passer dans le lait maternel pendant la première semaine suivant la transfusion. Si vous êtes ou devenez enceinte pendant l'étude un suivi spécifique sera réalisé au-delà des 30 jours de l'étude pour documenter l'issu de la grossesse.

**<u>Existe-t-il des risques non connus ?</u>**
Puisqu'il s'agit d'un traitement expérimental, il pourrait comporter d'autres risques que nous n'avons pas prévus ou que nous ne connaissons pas.

**VAIS-JE BÉNÉFICIER D'AVANTAGES EN PARTICIPANT À L'ETUDE ?**
Nous ne savons pas l'heure actuelle si la transfusion de plasma convalescent est efficace dans le traitement de la COVID-19, ni quelle est la dose optimale de plasma. En participant à l'étude, vous contribuerez à optimiser le traitement des patients qui seront atteints de COVID-19, dans le futur.

**EXISTE-T-IL D'AUTRES OPTIONS DE TRAITEMENT ?**
À l'heure actuelle, il n'existe pas de traitement spécifique contre le virus de la COVID-19. Par contre, d'autres médicaments ou traitements font actuellement l'objet de projets de recherche.

**DOIS-JE M'ATTENDRE A DES DEPENSES ET VAIS-JE RECEVOIR DES PAIEMENTS ?**
Aucune dépense ne devrait être encourue pour participer à l'étude. Vous recevrez les soins lors de votre hospitalisation et la participation ne demande pas de déplacements par la suite.

Vous ne recevrez pas de compensation financière pour votre participation à l'étude.

**COMMENT LA CONFIDENTIALITE EST-ELLE ASSUREE ?**
Durant votre participation à ce projet de recherche, le médecin responsable de ce projet ainsi que les membres de son personnel de recherche recueilleront, dans un dossier de recherche, les renseignements vous concernant et nécessaires pour répondre aux objectifs scientifiques de ce projet de recherche.

Seules les informations nécessaires aux objectifs de l'étude seront collectées. Le dossier d'étude inclura des données médicales, dont votre état de santé actuel et antérieur, votre mode de vie et les résultats des tests, examens et soins que vous recevrez durant le projet d'étude.

Tous les renseignements recueillis demeureront confidentiels dans les limites prévues par la loi. Vous serez uniquement identifié par un numéro de code. Le code permettant de vous identifier sera conservé par le médecin de l'étude de votre établissement. Pour assurer votre sécurité, une copie de ce formulaire d'information et de consentement, ainsi que le formulaire d'administration du plasma convalescent seront versés dans votre dossier médical. Ainsi, toute personne ou compagnie à qui vous donnerez accès à votre dossier médical aura accès à cette information.

Les données de l'étude seront conservées pendant au moins 25 ans par le médecin responsable de ce projet de recherche.

Les données de recherche pourront être publiées ou faire l'objet de discussions scientifiques, mais il ne sera pas possible de vous identifier.

Les données codées de l'étude seront partagées avec Héma-Québec, la Société Canadienne du Sang ainsi qu'avec l'équipe de recherche transfusionnelle de l'Université McMaster (promoteur de l'étude jumelle réalisée simultanément dans les autres provinces). Les données pourraient être partagées avec des chercheurs d'autres pays qui cherchent aussi un traitement pour la COVID-19. Cependant, aucune information d'identification personnelle ne sera partagée. Le promoteur et l'ensemble de ses partenaires respecteront les règles de confidentialités en vigueur au Québec et au Canada.

À des fins de surveillance, de contrôle, de protection, de sécurité, votre dossier de recherche ainsi que vos dossiers médicaux pourront être consultés par une personne mandatée par les autorités canadiennes de réglementation telles que Santé Canada, ainsi que par les représentants du promoteur de l'étude, de l'institution ou du comité éthique de la recherche. Toutes ces personnes et organismes adhèrent aux politiques de confidentialité.

Vous avez le droit de consulter votre dossier de recherche pour vérifier les renseignements recueillis et les faire rectifier au besoin.

**ETES-VOUS LIBRE DE PARTICIPER ET DE VOUS RETIRER?**

Votre participation à ce projet de recherche est volontaire. Vous êtes donc libre de refuser d'y participer. Vous pouvez également vous retirer de ce projet à n'importe quel moment, sans avoir à donner de raisons, en informant l'équipe de recherche. Par contre, les données déjà collectées seront conservées dans la base de données.

Votre décision de ne pas participer à ce projet de recherche ou de vous en retirer n'aura aucune conséquence sur la qualité des soins et des services auxquels vous avez droit ou sur votre relation avec les équipes qui les dispensent.

Le médecin responsable de ce projet de recherche, le comité d'éthique de la recherche, l'organisme subventionnaire ou le commanditaire peuvent mettre fin à votre participation, sans votre consentement. Cela peut se produire si de nouvelles découvertes ou informations indiquent que votre participation au projet n'est plus dans votre intérêt ou encore s'il existe des raisons administratives d'abandonner le projet.

Si vous vous retirez ou êtes retiré du projet, l'information déjà recueillie dans le cadre de ce projet sera néanmoins conservée, analysée ou utilisée pour assurer l'intégrité du projet.

Toute nouvelle connaissance acquise durant le déroulement du projet qui pourrait avoir un impact sur votre décision de continuer à participer à ce projet vous sera communiquée rapidement.

**QUI DOIS-JE APPELER SI J'AI DES QUESTIONS OU DES PROBLEMES ?**

Si vous avez des questions concernant le projet de recherche ou si vous rencontrez des problèmes vous semblant liés à votre participation au projet de recherche, vous pouvez appeler le chercheur principal du projet de votre hôpital :

CHU Sainte-Justine: Caroline Quach-Thanh (Chercheuse principale) : (514) 345-4931, poste 3827

Pour plus d'informations concernant le projet de recherche, merci de contacter : Marie-Christine Auclairresponsable de l'équipe de projet au CHU Sainte-Justine: (514) 345-4931, poste : 3827.

Si vous souhaitez plus d'informations concernant vos droits par rapport à votre participation à la recherche, vous pouvez contacter le représentant des sujets du bureau du commissaire aux plaintes et à la qualité (Ombudsman)au CHU Sainte-Justine: 514-345-4749.

**OU PUIS-JE OBTENIR PLUS D'INFORMATIONS ?**

Concernant les essais cliniques (en anglais seulement) : une description de cet essai clinique est disponible, selon la loi canadienne, à l'adresse suivante : http://www.clinicalTrials.gov. Ce site ne contient aucune information pouvant vous identifier. Il fournit un résumé des résultats du projet de recherche, une fois ceux-ci disponibles. Vous pouvez consulter le site internet à tout moment.

**COMITE D'ETHIQUE DE LA RECHERCHE**

Le comité d'éthique de la recherche du CHU Sainte-Justine a approuvé le projet et assurera le suivi du projet, pour les établissements du réseau de la santé et des services sociaux du Québec participants.

## ASSENTIMENT ET CONSENTEMENT

**Titre** : Essai clinique multicentrique, adaptatif, randomisé, ouvert et contrôlé sur la sécurité et l'efficacité du plasma de convalescence pour le traitement de la COVID-19 chez les patients hospitalisés.

______________________________

Nom du participant *(Lettres moulées)*

**Consentement verbal**

Acceptez-vous de participer, que le majeur que vous représentez participe ou que votre adolescent participe à ce projet de recherche ?

Oui □ Non □

Autorisez-vous l'équipe de recherche à consulter votre dossier médical, celui du majeur que je représente, ou le dossier médical de votre adolescent, pour obtenir les informations pertinentes à ce projet ?

Oui □ Non □

Consentement verbal donné par : ___________________________________

Lien avec le participant :___________________________________

Consentement verbal obtenu par : ___________________________________ Signature : ________________

Date : ________________

J'atteste avoir assisté à la discussion sur l'étude en date du _________

J'atteste que le patient a donné un consentement verbal en date du___________

Témoin : ___________________________ Signature : _________________________Date : ________________

**Consentement écrit**

On m'a expliqué la nature et le déroulement du projet de recherche. J'ai pris connaissance du formulaire de consentement et on m'en a remis ou transmis par voie électronique un exemplaire. J'ai eu l'occasion de poser des questions auxquelles on a répondu. Je confirme avoir donné mon consentement verbal en date du : ______________.

J'ai autorisé l'équipe de recherche à consulter mon dossier médical, celui du majeur que je représente, ou le dossier médical de mon enfant, pour obtenir les informations pertinentes à ce projet.

En acceptant de participer à ce projet de recherche, vous ne renoncez à aucun de vos droits et vous ne libérez pas le médecin responsable de ce projet de recherche, le commanditaire et l'établissement de leur responsabilité civile et professionnelle.

| ___________________________ | _____________________________ | ______________ |
|---|---|---|
| Nom du participant de plus de 18 ans,<br>Du parent ou tuteur ou du représentant<br>Légal *(Lettres moulées)* | Signature | Date |

**Assentiment de l'adolescent (moins de 18 ans) ou du majeur inapte capable de comprendre la nature de l'étud**

____________________________ ____________________________ _____________

Nom du participant *(Lettres moulées)* Signature Date

Ou

Assentiment verbal de l'enfant non en mesure de signer mais en mesure de comprendre la nature de l'étude obtenu par :___________________________________

J'ai expliqué au participant et/ou à son parent/tuteur et/ou à son représentant légal tous les aspects pertinents de la recherche et j'ai répondu aux questions qu'ils m'ont posées. Je leur ai indiqué que la participation au projet de recherche est libre et volontaire et que la participation peut être cessée en tout temps.

__________________________________ _____________________________ _________

Nom de la personne ayant obtenu le consentement *(Lettres moulées)* Signature Date

## ADDENDUM AU FORMULAIRE DE CONSENTEMENT

Participant atteignant la majorité (18 ans) pendant sa participation au projet de recherche

**Titre** : Essai clinique multicentrique, adaptatif, randomisé, ouvert et contrôlé sur la sécurité et l'efficacité du plasma de convalescence pour le traitement de la COVID-19 chez les patients hospitalisés.

J'ai revu aujourd'hui le consentement que mes parents avaient signé au moment de mon entrée dans ce projet de recherche et une copie de ce consentement signé m'a également été remise aujourd'hui ou me sera remise lorsque je ne serai plus en isolation.

**Consentement verbal**

Acceptez-vous de continuer votre participation à ce projet de recherche ?

Oui □ Non □

Autorisez-vous l'équipe de recherche à consulter votre dossier médical pour obtenir les informations pertinentes à ce projet ?

Oui □ Non □

Consentement verbal donné par : ________________________________

Consentement verbal obtenu par : ________________________________ Date : ________________

**Consentement écrit**

J'accepte de continuer ma participation à ce projet de recherche.

Je comprends que ma participation est libre et volontaire et que je peux me retirer en tout temps de ce projet de recherche.

J'autorise l'équipe de recherche à consulter mon dossier médical pour obtenir les informations pertinentes à ce projet.

______________________________________________________________________

| Nom du participant *(Lettres moulées)* | Signature | Date |
|---|---|---|

______________________________________________________________________

| Nom de la personne ayant obtenu le consentement *(Lettres moulées)* | Signature | Date |
|---|---|---|

**ADDENDUM AU FORMULAIRE DE CONSENTEMENT**

Participant redevenu apte au consentement pendant sa participation au projet de recherche

**Titre** : Essai clinique multicentrique, adaptatif, randomisé, ouvert et contrôlé sur la sécurité et l'efficacité du plasma de convalescence pour le traitement de la COVID-19 chez les patients hospitalisés.

J'ai revu aujourd'hui le consentement que mon représentant légal avait signé au moment de mon entrée dans ce projet de recherche et une copie de ce consentement signé m'a également été remise aujourd'hui ou me sera remise lorsque je ne serai plus en isolation.

**Consentement verbal**

Acceptez-vous de continuer votre participation à ce projet de recherche ?

Oui □ Non □

Autorisez-vous l'équipe de recherche à consulter votre dossier médical pour obtenir les informations pertinentes à ce projet ?

Oui □ Non □

Consentement verbal donné par : ________________________________

Consentement verbal obtenu par : ________________________________ Date : ________________

**Consentement écrit**

J'accepte de continuer ma participation à ce projet de recherche.

Je comprends que ma participation est libre et volontaire et que je peux me retirer en tout temps de ce projet de recherche.

J'autorise l'équipe de recherche à consulter mon dossier médical pour obtenir les informations pertinentes à ce projet.

______________________________________________________________________

| | | |
|---|---|---|
| Nom du participant<br>*(Lettres moulées)* | Signature | Date |

______________________________________________________________________

| | | |
|---|---|---|
| Nom de la personne ayant obtenu le consentement<br>*(Lettres moulées)* | Signature | Date |
